# The nociceptin receptor promotes autophagy through NF-kB signaling and is transcriptionally regulated by E2F1 in HCC

**DOI:** 10.1038/s41420-022-00978-7

**Published:** 2022-04-05

**Authors:** Xiaoshuang Zhou, Dongtai Chen, Yan Yan, Qiang Li, Wei Xing, Yanling Liu, Yonghua Chen, Dongyin Wang, Yunfei Yuan, Jingdun Xie, Weian Zeng, Jiahao Pan

**Affiliations:** 1 Department of Anesthesiology, Sun Yat-Sen University Cancer Center, State Key Laboratory of Oncology in South China, Collaborative Innovation Center for Cancer Medicine, 510060 Guangzhou, China; 2 Department of Anesthesiology, Huizhou Municipal Central Hospital, 516001 Huizhou, China; 3 Department of Anesthesiology & Operating Theatre, Sun Yat-sen University Cancer Center, State Key Laboratory of Oncology in South China, Collaborative Innovation Center for Cancer Medicine, 510060 Guangzhou, Guangdong China; 4 Department of Anesthesiology, Peking University Shenzhen Hospital, 518000 Shenzhen, China; 5 Department of Hepatobiliary Oncology, Sun Yat-Sen University Cancer Center, State Key Laboratory of Oncology in South China, Collaborative Innovation Center for Cancer Medicine, 510060 Guangzhou, China

**Keywords:** Cancer epigenetics, Cancer therapy

## Abstract

Opioids and their receptors are involved in cancer progression. However, the roles of the nociceptin receptor (NOP) and its antagonist (JTC801) in hepatocellular carcinoma (HCC) are poorly understood. The prognostic value of NOP expression was evaluated using tissue microarray and immunohistochemical staining analyses in a human HCC cohort. The biological role and mechanism of NOP in HCC tumor growth were determined in vitro and in vivo. We found that NOP was associated with the clinicopathological features and survival outcomes of HCC patients. NOP overexpression promoted HCC growth in vitro and in vivo. Mechanistically, NOP activated NF-kB signaling to promote autophagy, which inhibited apoptosis, in HCC cells. An inhibitor of autophagy, 3-MA, and an inhibitor of NF-kB, JSH-23, attenuated the function of NOP in HCC. E2F1 was identified as a transcription factor of NOP. The oncogenic role of NOP was positively regulated by E2F1. Furthermore, JTC801, a selective antagonist of NOP, abolished the function of NOP by inhibiting NF-kB signaling and autophagy. Our study demonstrates that NOP is an oncogene in HCC. We provide a potential therapeutic candidate and prognostic predictor for HCC. JTC801 could become a potential drug for HCC therapy.

## Introduction

Hepatocellular carcinoma (HCC) is the fourth leading cause of cancer death [1]. Related factors, such as chronic hepatitis B or C infection, exposure to aflatoxins, alcohol abuse, and nonalcoholic steatohepatitis, have been acknowledged as risk factors for HCC development and progression [2]. However, the definite molecular mechanism of HCC should be further investigated.

The nociceptin receptor (NOP), also called the nociceptin/orphanin FQ (N/OFQ) receptor or kappa-type 3 opioid receptor, is a member of the 7 transmembrane-spanning G protein-coupled receptor (GPCR) family and functions as a receptor for the endogenous opioid-related neuropeptide nociceptin/orphanin FQ [3]. The NOP receptor system plays a significant role in the regulation of pain. The spinal NOP receptor system attenuates injury-induced hyperalgesia by directly inhibiting projection neurons in the spinal cord that sends nociceptive signals to the brain, not by inhibiting presynaptic terminals of DRG neurons in the superficial lamina [4]. NOP was also found to contribute to the anti-hypersensitivity effect of cebranopadol in a rat model of arthritic pain [5]. Furthermore, systemic activation of NOP receptors produced bidirectional changes in pain sensitivity.

The NOP receptor system is a target for alleviation of cancer-induced bone pain in rats [6]. Abundant evidence indicates that GPCRs are involved in a variety of biological processes across cancer types, including invasion, migration, and vascular remodeling [7]. NOP expression has been reported to be significantly higher in cancer patients and postoperative patients than in healthy controls [8].

A previous study has reported that NOP is frequently amplified in metastatic conjunctival melanomas [9], suggesting its potential oncogenic role in cancer. NOP is also overexpressed in non-small-cell lung cancer and predicts a poor prognosis [10]. However, the role of NOP in HCC is poorly understood.

*N*-(4-amino-2-methylquinolin-6-yl)-2-(4-ethylphenoxymethyl) benzamide monohydrochloride (JTC801) is a high-affinity and selective antagonist of NOP and belongs to the GPCR family [11]. JTC801 has been found to reverse pain and anxiety symptoms in a rat model of posttraumatic stress disorder [12]. We also previously found that JTC801 alleviates mechanical allodynia in paclitaxel-induced neuropathic pain through the PI3K/Akt pathway [13]. It has been reported that JTC801 induces pH-dependent cell death (alkaliptosis) in cancer cells by downregulating the expression of carbohydrate antigen 9 [14]. Zheng et al. elucidated the antitumor effects of JTC801 on human osteosarcoma cells [15]. JTC801 also inhibits the proliferation and metastasis of HCC cells and ovarian cancer cells in a PI3K/PKB-dependent manner [16, 17]. However, the exact biological roles and mechanisms of JTC801 in HCC remain undetermined.

The role of NOP in HCC remains poorly understood. In this study, we screened for differentially expressed pain-related genes and identified NOP as a potential oncogene in HCC. We aimed to investigate the clinicopathological significance of NOP expression in HCC patients. Furthermore, we explored the effect of NOP on HCC in vivo and in vitro and investigated its underlying molecular mechanisms. The effects of the NOP agonist nociceptin and the NOP antagonist JTC801 were also examined by proliferation assays in vitro and with subcutaneous xenograft HCC models in vivo.

## Results

### NOP is a prognostic factor in HCC

**Fig. 1:**
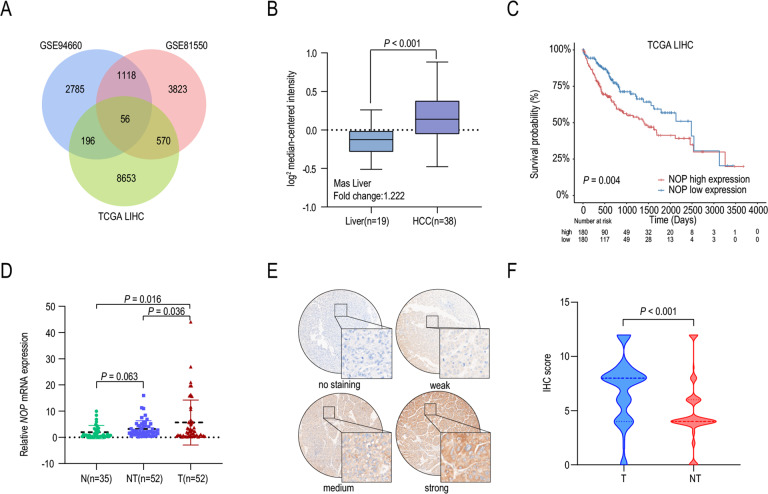
NOP is upregulated and predicts poor prognosis in HCC. **A** Venn diagram of the differentially expressed genes in three HCC datasets. **B** Boxplots of NOP mRNA expression in HCC (*n* = 38) and adjacent tissue (*n* = 19) in the Mas Liver dataset. **C** Kaplan–Meier analysis based on NOP expression in the TCGA dataset (*n* = 360). **D** Boxplots of NOP mRNA expression in normal tissue (N) (*n* = 35), HCC (T) (*n* = 52), and adjacent tissue (NT) (*n* = 52) in the SYSUCC cohort. **E** IHC staining showing strong, moderate, and weak positive expression and negative expression of NOP in HCC. Scale bar: ×4 = 100 μm; ×40 = 10 μm. **F** Boxplots of the IHC score of NOP in HCC (*n* = 288) and adjacent tissue (*n* = 288).

Differential expression analysis was performed in three HCC datasets (GSE94660, GSE81550, and TCGA-LIHC) to identify the key genes associated with the progression of HCC, and 56 overlapping genes were identified (Fig. 1A). We found that NOP was more highly expressed in HCC than in normal liver tissue in the Mas Liver dataset (Fig. 1B). Survival analysis in TCGA datasets showed that patients with higher NOP expression exhibited worse survival (Fig. 1C). We also analyzed NOP expression in the SYSUCC cohort. NOP mRNA expression in tumor tissue was significantly higher than that in normal and adjacent tissues (Fig. 1D). Immunohistochemical (IHC) staining revealed that the protein level of NOP in HCC tissue was also higher than that in adjacent tissue and that NOP was localized in the cytoplasm in HCC tissue (Fig. 1E, F).

**Fig. 2:**
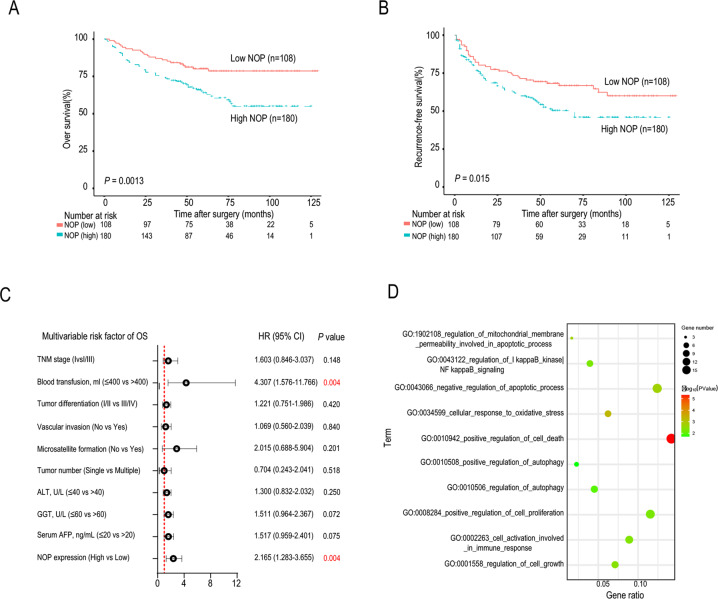
NOP predicts poor prognosis in HCC. **A**, **B** Kaplan–Meier analysis based on NOP expression in the SYSUCC cohort. **C** Forest plot showing prognostic factors associated with overall survival (*P* = 0.004). **D** Bubble plot of Gene Ontology analysis results for differentially expressed genes identified by RNA-seq.

Higher NOP expression was associated with worse overall survival and recurrence-free survival in the SYSUCC cohort (Fig. 2A, B). NOP expression was associated with microsatellite formation, tumor number, tumor size, tumor encapsulation, and TNM stage (Table 1). Univariate and multivariate analyses suggested that NOP is an independent prognostic factor of overall survival in HCC (Fig. 2C and Table 2).

**Table 1:** Correlation of NOP expression with the clinicopathologic features of 288 patients with HCC.

Characteristics	Cases	NOP protein		*P*
		Low expression	High expression	
Sex				
Female	36	13	23	1.000
Male	252	95	157	
Age (years)				
13–50	142	55	87	0.716
51–77	146	53	93	
Hepatitis B surface antigen				
Negative	38	16	22	0.591
Positive	250	92	158	
Child–Pugh classification^a^				
A	284	107	177	1.000
B	3	1	2	
Serum AFP (μg/L)				
≤20	124	48	76	0.714
>20	164	60	104	
Microsatellite formation				
No	246	100	146	**0.009**
Yes	42	8	34	
Tumor number				
Single	233	97	136	**0.003**
Multiple	55	11	44	
Tumor size (cm)				
≤5	147	64	83	**0.038**
>5	141	44	97	
Tumor encapsulation				
No/incomplete	216	89	127	**0.025**
Yes	72	19	53	
Vascular invasion				
No	235	86	149	0.532
Yes	53	22	31	
Liver cirrhosis				
No	75	31	44	0.488
Yes	213	77	136	
Differentiation grade				
I/II	217	79	138	0.572
III/IV	71	29	42	
TNM stage				
I	177	77	100	**0.009**
II/III	111	31	80	

Bold values indicate *P* < 0.05.

^a^No patient with Child–Pugh class C was found.

**Table 2:** Univariate analysis of factors associated with OS and RFS in HCC patients (*n* = 288).

Variables	Overall survival			Recurrence-free survival		
	HR	95% CI	*P*^a^	HR	95% CI	*P*^a^
Gender (female vs male)	0.670	0.324–1.385	0.279	0.576	0.301–1.102	0.096
Age (≤50 vs >50)	1.189	0.783–1.806	0.417	0.897	0.626–1.288	0.557
HBsAg (positive vs negative)	0.854	0.474–1.538	0.599	1.566	0.842–2.914	0.156
Child–Pugh classification (A vs B)	0.179	0.044–0.730	0.016	0.609	0.085–4.369	0.622
Serum AFP, ng/ml (≤20 vs >20)	1.662	1.070–2.580	0.024*	0.965	0.672–1.386	0.847
GGT, U/l (≤60 vs >60)	1.925	1.269–2.920	0.002*	1.707	1.189–2.451	0.004*
ICGR15 ≤10% vs >10%)	0.970	0.502–1.874	0.928	1.501	0.896–2.513	0.123
AST, U/l (≤40 vs >40)	1.380	0.906–2.101	0.134	1.646	1.144–2.367	0.007*
ALT, U/l (≤40 vs >40)	1.720	1.133–2.610	0.011*	1.657	1.154–2.380	0.006*
Tumor number (single vs multiple)	2.104	1.331–3.325	0.001*	1.826	1.191–2.799	0.006*
Tumor size, cm (≤5 vs >5)	1.419	0.935–2.155	0.100	1.583	1.100–2.276	0.013*
Microsatellite formation (no vs yes)	2.474	1.526–4.010	<0.001	2.845	1.826–4.433	<0.001*
Tumor capsule (no vs yes)	1.039	0.642–1.682	0.876	1.159	0.769–1.747	0.480
Vascular invasion (no vs yes)	1.988	1.209–3.269	0.007*	1.253	0.772–2.033	0.361
Intraoperative blood loss, ml (≤400 vs >400)	0.952	0.573–1.581	0.848	1.316	0.873–1.983	0.190
blood transfusion, mL (≤400 vs >400)	3.079	1.248–7.599	0.015*	0.743	0.184–3.010	0.678
Resection margin, cm ≤1 vs >1)	0.639	0.388–1.052	0.078	0.769	0.513–1.152	0.202
Cirrhosis (absent vs present)	1.282	0.772–2.130	0.337	1.256	0.817–1.930	0.299
Tumor differentiation (I/II vs III/IV)	1.778	1.145–2.760	0.010*	1.465	0.980–2.192	0.063
TNM stage (I vs I/III)	2.514	1.652–3.824	<0.001*	1.715	1.189–2.472	0.004*
NOP expression (high vs low)	2.160	1.333–3.501	0.002*	1.607	1.088–2.375	0.017*

*Statistical significance was set to *P* < 0.05.

^a^Cox proportional hazards regression model.

### NOP influences the proliferation and apoptosis of HCC cells through autophagy

qPCR and western blot analyses showed that NOP was highly expressed in Hep3B and Huh7 cells and expressed at low levels in MHCC-LM3 and PLC-8024 cells (Supplementary Fig. 1A). Therefore, we overexpressed NOP in MHCC-LM3 and PLC-8024 cells (Supplementary Fig. 1B). NOP was knocked down in Hep3B and Huh7 cells (Supplementary Fig. 1C). To explore the underlying mechanisms of the role of NOP in HCC, we conducted RNA sequencing to identify genes modified by NOP overexpression using PLC-8024-vector and PLC-8024-NOP cells. Pathway analysis showed that apoptosis, the NF-kB signaling pathway, and autophagy were the top three canonical pathways (Fig. 2D).

**Fig. 3:**
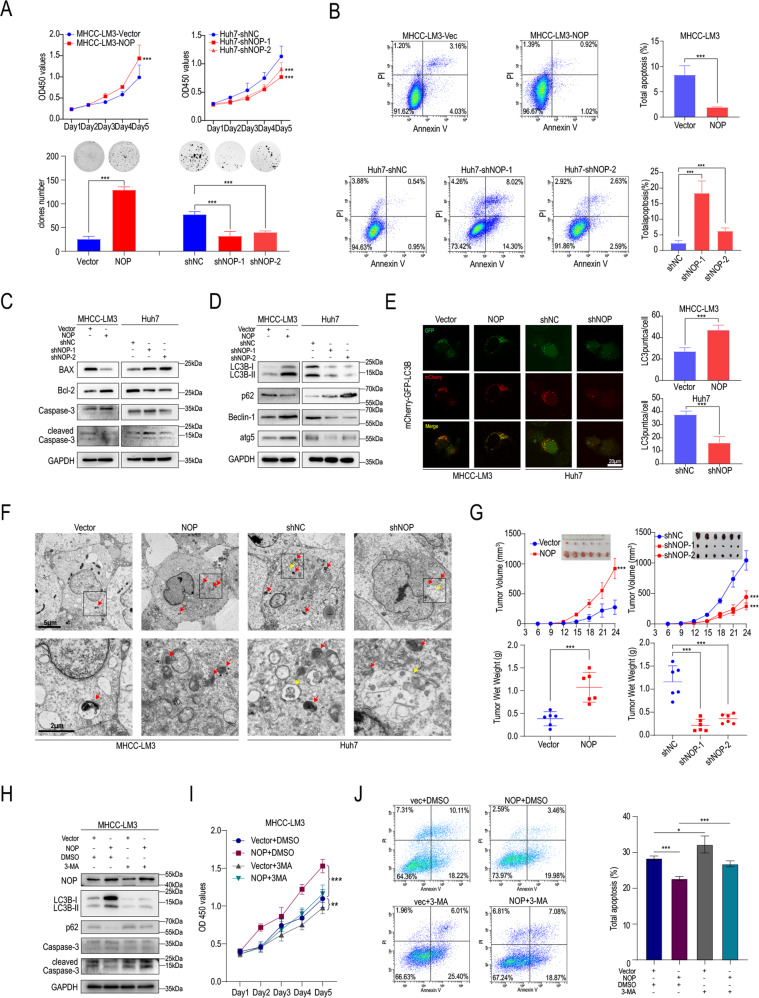
NOP influences the proliferation and apoptosis of HCC cells through autophagy. **A** CCK-8 assay and colony formation assay showing the proliferation of MHCC-LM3-vector, MHCC-LM3-NOP, Huh7-shNC, Huh7-shNOP-1, and Huh7-shNOP-2 cells. **B** Apoptosis rates in MHCC-LM3-vector, MHCC-LM3-NOP, Huh7-shNC, Huh7-shNOP-1, and Huh7-shNOP-2 HCC cells. **C** IB analysis of the indicated proteins associated with apoptosis in MHCC-LM3 and Huh7 cells. **D** IB analysis of the indicated proteins associated with autophagy in MHCC-LM3 and Huh7 cells. **E** IF staining showing the GFP-mCherry-LC3B fusion protein in MHCC-LM3-vector, MHCC-LM3-NOP, Huh7-shNC, and Huh7-shNOP cells (GFP: green, mCherry: red; merged: yellow). Scale bars = 20 μm. **F** Electron microscopy showing the autolysosome (red arrow) and autophagosome (yellow arrow) numbers in MHCC-LM3-vector, MHCC-LM3-NOP, Huh7-shNC, and Huh7-shNOP cells. Scale bars = 2 μm. **G** Volumes and weights of xenograft tumors formed from MHCC-LM3-vector, MHCC-LM3-NOP, Huh7-shNC, Huh7-shNOP-1, and Huh7-shNOP-2 cells. **H** IB analysis of the indicated proteins associated with apoptosis and autophagy in MHCC-LM3 cells treated with 3-MA. **I** CCK-8 assay showing the proliferation of MHCC-LM3-vector and MHCC-LM3-NOP HCC cells treated with 3-MA or DMSO. **J** Apoptosis rates in MHCC-LM3-vector and MHCC-LM3-NOP HCC cells treated with 3-MA or DMSO. ***P < 0.001, **P < 0.01, *P < 0.05.

Then, we sought to determine whether NOP can influence the development and progression of HCC. Figure. 3A shows that NOP overexpression significantly promoted while knockdown of NOP significantly inhibited the proliferation of HCC cells (Fig. 3A and Supplementary Fig. 2A). Furthermore, NOP inhibited apoptosis in HCC cells, and knockdown of NOP promoted apoptosis (Fig. 3B and Supplementary Fig. 2B). Consistent with the RNA-seq results, western blotting confirmed that NOP overexpression upregulated Bcl-2 expression and decreased the protein levels of BAX and cleaved Caspase-3. In contrast, NOP knockdown increased BAX and cleaved caspase 3 levels and downregulated BCL-2 expression (Fig. 3C). Then, we investigated the effect of NOP on autophagy. As shown in Fig. 3D and Supplementary Fig. 2C, NOP overexpression upregulated the expression of LC3B, Beclin1, and atg5 and downregulated the expression of P62. NOP knockdown upregulated p62 expression and downregulated LC3B, Beclin1, and atg5 expression. Consistent with the RNA-seq results, we found that NOP promoted autophagy in HCC cells. LC3B can be used to identify autophagosomes (GFP-positive and mCherry-positive; appear yellow in merged images) and autolysosomes (GFP-negative and mCherry-positive; appear red in merged images), and both yellow punctate fluorescence and red punctate fluorescence increase when autophagy is activated. Analysis of the distribution of the GFP-mCherry-LC3B fusion protein in NOP-overexpressing HCC cells revealed increases in both red and yellow fluorescence, which also caused increases in both red and yellow punctate fluorescence, indicating that autophagic flux had increased. As expected, the opposite effect was observed in Huh7-NOP-sh cells (Fig. 3E and Supplementary Fig. 2D). Electron microscopy was employed to observe the numbers of autolysosomes (red arrow) and autophagosomes (yellow arrow). NOP overexpression increased the numbers of autolysosomes and autophagosomes, while fewer autolysosomes and autophagosomes were observed in NOP-knockdown cells (Fig. 3F and Supplementary Fig. 2E). Next, we investigated the functional role of NOP in vivo. The results showed that tumor growth was promoted in NOP-overexpressing MHCC-LM3 cells and suppressed in NOP-knockdown Huh7 cells compared with the control cells (Fig. 3G). Next, we sought to determine whether NOP promotes HCC progression by promoting autophagy. 3-MA, an autophagy inhibitor, rescued Caspase-3 cleavage in MHCC-LM3 cells (Fig. 3H). 3-MA also reversed the effects of NOP overexpression on proliferation and apoptosis (Fig. 3I, J).

### NOP promotes autophagy by activating the NF-kB signaling pathway

**Fig. 4:**
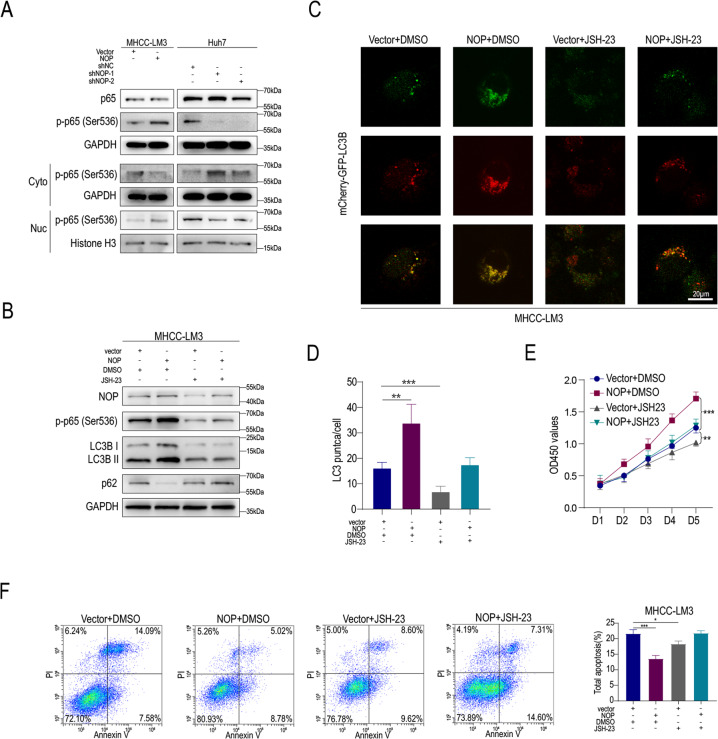
NOP promotes autophagy by activating the NF-kB signaling pathway. **A** IB analysis of the indicated proteins associated with the NF-kB pathway in MHCC-LM3 and Huh7 cells. **B** IB analysis of the indicated proteins associated with the NF-kB pathway in MHCC-LM3 and Huh7 cells treated with JSH-23 or DMSO. **C**, **D** IF staining showing LM3 expression in MHCC-LM3-vector and MHCC-LM3-NOP HCC cells treated with JSH-23 or DMSO. Scale bars = 20 μm. **E** CCK-8 assay showing the proliferation of MHCC-LM3-vector and MHCC-LM3-NOP HCC cells treated with JSH-23 or DMSO. **F** Apoptosis rates in MHCC-LM3-vector and MHCC-LM3-NOP HCC cells treated with JSH-23 or DMSO. ***P < 0.001, **P < 0.01, *P < 0.05.

As shown in Fig. 4A, NOP overexpression increased the levels of total and nuclear p-p65 but decreased the level of cytoplasmic p-p65. NOP knockdown decreased the total p-p65 level and increased the cytoplasmic p-p65 level. Furthermore, JSH-23, an inhibitor of the NF-kB pathway, decreased LM3 expression in NOP-overexpressing cells (Fig. 4B). JSH-23 also downregulated p62 expression in MHCC-LM3 cells (Fig. 4B). Immunofluorescence (IF) staining showed the distribution of the GFP-mCherry-LC3B fusion protein in MHCC-LM3 HCC cells. NOP expression was associated with increased red and yellow fluorescence, which also caused increases in both red and yellow punctate fluorescence. In addition, JSH-23 treatment reversed the effects of NOP on autophagy, and treated cells presented decreased red and yellow fluorescence (Fig. 4C). Proliferation was inhibited and apoptosis was promoted in NOP-overexpressing HCC cells treated with JSH-23 (Fig. 4D, E). These results indicate that NOP promotes autophagy by activating the NF-kB signaling pathway and that inhibition of the NF-kB pathway reverses the function of NOP in HCC.

### E2F1 is a transcription factor of NOP

**Fig. 5:**
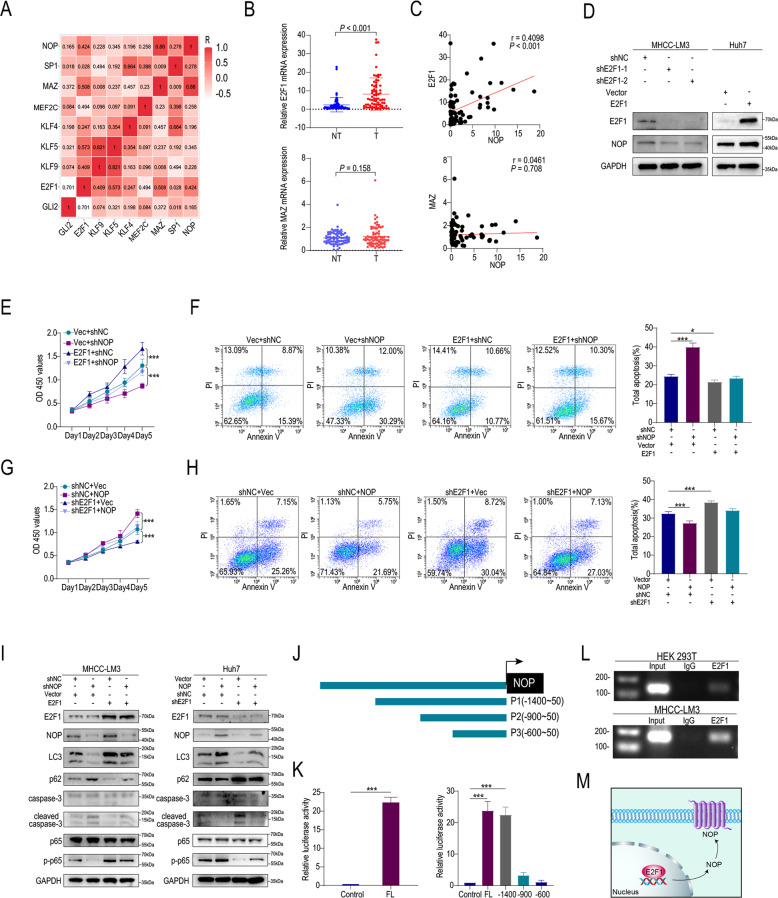
E2F1 is a transcription factor of NOP. **A** Correlations between NOP expression and the expression of candidate transcription factors. **B** Expression of E2F1 and MAZ in HCC and adjacent tissues. **C** Correlation between NOP expression and E2F1 or MAZ expression. **D** IB analysis of the indicated proteins in MHCC-LM3 and Huh7 cells. **E** CCK-8 assay showing the proliferation of vector + shNC, vector + shNOP, E2F1 + shNC, and E2F1-shNOP HCC cells. **F** Apoptosis rates in vector + shNC, vector + shNOP, E2F1 + shNC, and E2F1-shNOP HCC cells. **G** CCK-8 assay showing the proliferation of shNC + vector, shNC + NOP, shE2F1 + vector, and shE2F1 + shNOP HCC cells. **H** Apoptosis rates in shNC + vector, shNC + NOP, shE2F1 + vector, and shE2F1 + shNOP HCC cells. **I** IB analysis of the indicated proteins associated with autophagy and apoptosis in MHCC-LM3 and Huh7 cells. **J** Schematic drawing of the full-length and truncated NOP fragments. **K** Luciferase assay showing the binding site of NOP and E2F1. L. A specific band of the expected size was detected in the input DNA and the anti-E2F1 antibody (anti-E2F1)-precipitated DNA by ChIP. **M** Schematic showing the effect of increased expression of NOP via the transcription factor E2F1. ***P < 0.001, *P < 0.05.

We next investigated why NOP is upregulated in HCC. To elucidate the upstream regulator of NOP, we searched the JASPAR database and found eight candidate transcription factors that might regulate the expression of NOP. Correlation analysis was performed to identify transcription factors of NOP. The most strongly correlated genes were E2F1 and MAZ (Fig. 5A). The qRT–PCR results revealed that E2F1 was upregulated in HCC tissues compared with peritumor tissues (Fig. 5B). Further analysis showed that the E2F1 level was associated with the NOP mRNA level, whereas the association of MAZ with NOP was not significant (Fig. 5C). Western blotting showed that E2F1 overexpression led to an increased protein level of NOP in Huh7 cells and that NOP knockdown decreased the protein level of NOP in MHCC-LM3 cells (Fig. 5D). The Cell Counting Kit-8 (CCK-8) assay showed that E2F1 reversed the growth inhibition and apoptosis caused by NOP knockdown in Huh7 cells. E2F1 knockdown inhibited proliferation and promoted apoptosis in NOP-overexpressing HCC cells (Fig. 5E–H). Additionally, E2F1 knockdown decreased LC3 and p-p65 protein levels and increased the cleaved Caspase3 level in NOP-overexpressing cells (Fig. 5I). The NOP upstream region (−1 to −2000 kb) was analyzed with JASPAR (http://jaspar.genereg.net/), and three binding sites for E2F1 were predicted to exist in the putative promoter region (Fig. 5J). We speculated that the DNA binding sites for E2F1 were −1.4 kb to −0.9 kb from the promoter. Compared with the activity of the FL (full length) promoter, the activity of the P1 (−1.4 kb) promoter was not significantly different, and marked decreases in the activity of the P2 (−0.9 kb) and P3 (−0.5 kb) promoters were observed (Fig. 5K). To determine whether E2F1 bound directly to the NOP promoter, we performed a chromatin immunoprecipitation (ChIP) assay with an anti-E2F1 antibody, and the results demonstrated an interaction between E2F1 and the NOP promoter (Fig. 5L). These results indicate that E2F1 is a transcription factor of NOP.

### JTC801 suppresses HCC progression through the NOP-autophagy pathway

**Fig. 6:**
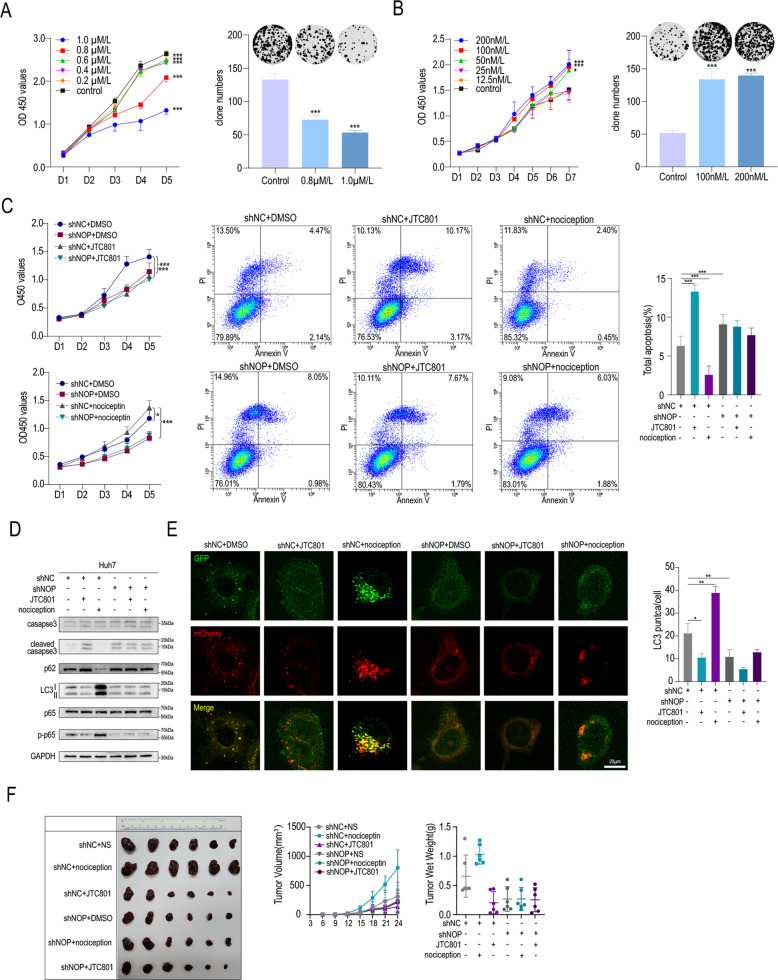
JTC-801 suppresses HCC progression through the NOP-autophagy pathway. **A** CCK-8 and colony-formation assays showing the proliferation of HCC cells treated with various concentrations of JTC801. **B** CCK-8 and colony-formation assays showing the proliferation of HCC cells treated with various concentrations of nociceptin. **C** CCK-8 assay and apoptosis analysis showing the proliferation of shNC and shNOP HCC cells treated with DMSO, JTC-801 or nociceptin. **D** IB analysis of the indicated proteins associated with NF-kB, autophagy, and apoptosis in Huh7 cells treated with JTC-801 or nociceptin. **E** IF staining showing LM3 expression in shNC and shNOP HCC cells treated with JTC-801 or nociceptin. Scale bars = 20 μm. **F** shNC and shNOP HCC cell-derived xenograft tumors in mice treated with JTC-801 or nociceptin. ***P < 0.001, **P < 0.01, *P < 0.05.

To evaluate whether JTC801 exhibits anticancer activity in HCC, we treated PLC-8024 cells with JTC801 at various concentrations. CCK-8 and colony formation assays demonstrated that JTC801 inhibited the proliferation of HCC cells in a concentration-dependent manner (Fig. 6A). In contrast, nociceptin, an agonist of NOP, promoted the growth and colony formation of HCC cells (Fig. 6B). NOP knockdown abolished the effects of JTC801 and nociceptin on proliferation and apoptosis (Fig. 6C). As expected, the effects of JTC801 and nociceptin on the levels of LC3, p-p65, and cleaved Caspase3 and on autophagy were also counteracted when NOP was knocked down (Fig. 6D). IF analysis suggested that nociceptin treatment increased red and yellow fluorescence, indicating that it increased autophagy. The opposite effect was observed in cells treated with JTC801. Furthermore, the effect of nociceptin on LC3 puncta was also abolished with NOP knockdown (Fig. 6E). It has been reported that autophagy and the NF-kB signaling pathway are associated with drug resistance [18, 19]. Thus, we next explored the role of JTC-801 in chemoresistance. As expected, JTC-801 had synergistic effects with doxorubicin (Supplementary Fig. 3). In vivo, nociceptin and JTC801 promoted and inhibited tumor growth, respectively, and these effects were also abolished with NOP knockdown (Fig. 6F).

## Discussion

Although it is widely recognized that opioids and their receptors are involved in cancer progression, the role of NOP in HCC and its molecular mechanism are still unclear. In the current study, we attempted to investigate the potential biological function and molecular mechanism of NOP in HCC progression. Our results proved that NOP was upregulated in HCC tissues. High NOP expression predicted an unfavorable OS and an increased recurrence probability in HCC patients. In vitro and in vivo functional experiments revealed that NOP significantly facilitated HCC cell proliferation.

**Fig. 7:**
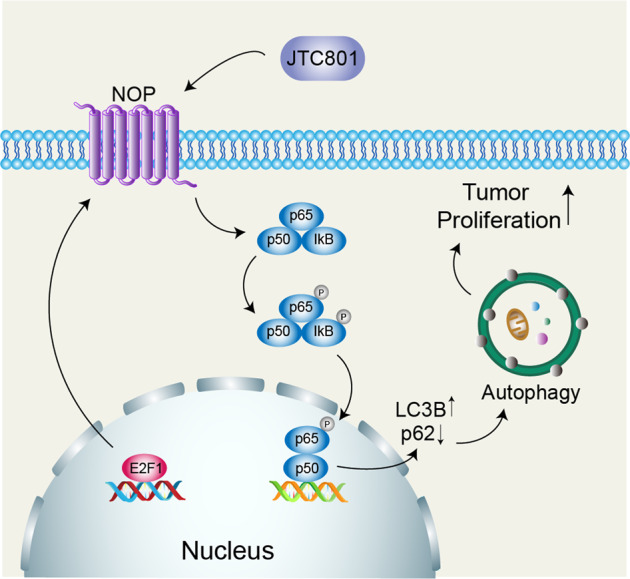
Working model. Proposed model of the mechanism by which NOP activates NF-kB signaling to promote autophagy, which inhibits apoptosis in HCC.

E2F1 is a member of the E2F family. Studies have proven its role in the cell cycle, DNA replication and repair, apoptosis, and checkpoint responses [20–23]. The RB/E2F1 pathway is a master regulator of cancer cell metabolism in advanced disease [24]. We found that E2F1 is a transcription factor and can regulate the expression of NOP. Consistent with previous studies, our study revealed that E2F1 plays an oncogenic role and reverses the effects of NOP knockdown, including apoptosis and cell proliferation. Furthermore, Fogal et al. reported that E2F1 interacts with p53 and enhances the nuclear retention of Ser315-phosphorylated p53, which is associated with autophagy [25]. The E2F1/USP11 positive feedback loop promotes hepatocellular carcinoma metastasis and inhibits autophagy by activating the ERK/mTOR pathway [26]. In our study, we proved that E2F1 regulates autophagy by upregulating NOP expression.

Autophagy acts as a double-edged sword in cancer development. Macroautophagy (hereafter referred to as autophagy) is an evolutionarily ancient and highly conserved catabolic process involving the formation of double-membraned vesicles called autophagosomes that engulf cellular proteins and organelles for delivery to the lysosome [27]. Cancer cells rely on autophagy; this reliance is likely due to inherent deficiencies in the microenvironment and to the increased metabolic and biosynthetic demands imposed by deregulated proliferation. For example, basal autophagy is upregulated in hypoxic tumor regions, where it is essential for tumor cell survival. Autophagy is also upregulated in RAS-transformed cancer cells and promotes their growth, survival, tumorigenesis, invasion, and metastasis [28]. Autophagy promotes tumor growth in many ways, including via suppression of p53 activation [29]. However, autophagy also suppresses breast cancer metastasis by degrading NBR1 [30]. It has been reported that autophagy mediates the proliferation and progression of HCC by inhibiting apoptosis [31, 32]. Our data showed that NOP knockdown upregulated P62 expression and downregulated LC3B, Beclin1, and atg5 expression. This result indicates that NOP inhibits autophagy in HCC cells. Rescue experiments demonstrated that NOP promotes HCC progression by promoting autophagy. The autophagy inhibitor 3-MA rescued Caspase-3 cleavage in NOP-overexpressing cells. 3-MA also inhibited proliferation and promoted apoptosis in NOP-expressing HCC cells.

NF-kB is well understood in cancer and is involved in many molecular processes [33]. Previous studies have confirmed that NF-kB promotes apoptosis and autophagy in various cancer types [34–36]. Exosomal miR-1910-3p promotes autophagy in breast cancer cells by activating the NF-κB signaling pathway [37]. It has been reported that morroniside attenuates apoptosis by inhibiting NF-κB signaling [38]. RNA sequencing revealed that the NF-kB signaling pathway was one of the most significantly enriched pathways after NOP overexpression. In our study, we found that NOP can activate NF-kB signaling, which could explain its function in autophagy and apoptosis. Recently, an NOP ligand, MCOPPB, was reported to increase lipid accumulation, TLR (NF-kB) signaling and inflammation in the liver [39]. The results of JSH-23 treatment also revealed that NF-kB inhibition can reverse the influence of NOP overexpression on HCC cells. However, autophagy has also been found to regulate the activity of NF-kB signaling in many other studies [40, 41]. Therefore, a positive feedback loop might exist in the NOP-autophagy pathway, which should be explored in the future.

We found that JTC801 inhibited NF-kB signaling. JTC801 is a selective antagonist of NOP; thus, we sought to determine whether it influences autophagy and NF-kB signaling through NOP. Xinxin Song et al. demonstrated that JTC801 induces pH-dependent death specifically in cancer cells and slows the growth of tumors in mice; however, this effect is not achieved through the classical μ receptor [14]. Our data indicate that JTC801 inhibits the oncogenic effects of NOP in vivo and in vitro. JTC801 treatment decreased LC3 and p-p65 levels and increased the cleaved Caspase3 level. However, NOP knockdown abolished the antitumor effects of JTC801, indicating that NOP is indispensable for the anticancer activity of JTC801 in HCC. Therefore, our study constitutes the first report of the role of JTC801 in autophagy and NF-kB signaling. More importantly, we reveal that JTC801 acts as a tumor suppressor in HCC. We also report that JTC-801 can overcome chemoresistance in HCC and that JTC801 could become a potential drug for HCC therapy.

In conclusion, the present study found that NOP exhibited increased expression in HCC and promoted tumor growth in vitro and in vivo. NOP was closely associated with the clinicopathological features and survival outcomes of HCC patients. The oncogenic role of NOP was positively regulated by E2F1. NOP activated NF-kB signaling to promote autophagy, which inhibited apoptosis in HCC cells. Additionally, we revealed that 3-MA and JSH-23 attenuated the function of NOP in HCC. Furthermore, JTC801, a selective antagonist of NOP, abolished the function of NOP by inhibiting NF-kB signaling and autophagy (Fig. 7). Our research suggests that NOP might be a potential therapeutic candidate and prognostic predictor in HCC and that JTC801 could become a potential drug for HCC therapy.

## Materials and methods

### Cell culture

Established human liver cancer cell lines (MCHH-LM3, Huh7, PLC-8024, and Hep 3B) were obtained from the Chinese Type Culture Collection (Shanghai, China). All cell lines were validated by short tandem repeat (STR) profiling. All cell lines were cultured in DMEM supplemented with 10% fetal bovine serum. Real-time PCR was performed regularly to ensure that the cells were not contaminated with mycoplasma.

### CCK-8 assay

A CCK-8 kit (Dojindo, Kumamoto, Japan) was utilized to detect cell viability and proliferation. Cells were seeded into 96-well tissue culture plates at a density of 1000 cells per well. The OD value was measured at 450 nm following a 2-h incubation with 10 μl of CCK-8 solution per well.

### Colony formation assay

Cells were seeded in 6-well culture plates at a density of 1000 cells per well. The cells were further cultured for an additional 14 days. Then, the colonies were stained with crystal violet and subsequently analyzed by ImageJ software (ImageJ.nih.gov).

### Flow cytometry

To assess apoptosis, cells were pretreated with FITC Annexin V/PI (BD Pharmingen, USA) according to the manufacturer’s instructions.

### Western blot analysis

Protein lysates were loaded into 7.5–15% SDS–PAGE gels, subjected to electrophoretic separation and then transferred to PVDF membranes (Millipore). After the membranes were blocked with 5% skim milk powder, they were incubated with primary antibodies and corresponding secondary antibodies. The target protein bands were visualized with enhanced chemiluminescence reagents (Tanon 180-501). The following antibodies were used: anti-NOP (1:1000, 12970-1-AP, Proteintech), anti-BAX (1:1000, 2772s, Cell Signaling Technology), anti-Bcl-2 (1:1000, 15071t, Cell Signaling Technology), anti-Caspase-3 (1:1000, 9665s, Cell Signaling Technology), anti-cleaved Caspase-3 (1:1000, 9661, Cell Signaling Technology), anti-LC3B (1:1000, 12741, Cell Signaling Technology), anti-p62 (1:1000, 8025, Cell Signaling Technology), anti-Beclin-1 (1:1000, 3738, Cell Signaling Technology), anti-atg5 (1:1000, 9980, Cell Signaling Technology), anti-p65 (1:1000, 8242, Cell Signaling Technology), anti-p-p65 (1:1000, 3033, Cell Signaling Technology), anti-E2F1 (1:1000, 3742, Cell Signaling Technology), and anti-GAPDH (1:5000, 2118, Cell Signaling Technology).

### Immunofluorescence

Cells were seeded into glass coverslips in 8-well plates and fixed with 4% paraformaldehyde at room temperature for 15 min before permeabilization with 0.2% Triton X-100 in PBS for 10 min. The cells were then blocked with 5% BSA for 1 h and incubated with an anti-LC3B (1:400, ARG55251, Arigobio) antibody followed by the corresponding secondary antibodies. The cells were counterstained with DAPI, and coverslips were mounted prior to image acquisition. ImageJ software (ImageJ.nih.gov) was employed for the quantification of LC3B fluorescence.

### IHC analysis of clinical HCC specimens

All human tissue research in this study was conducted according to protocols approved by the Sun Yat-sen University Cancer Center, Guangzhou, China. The HCC tissue sections were stained with antibodies against NOP.

IHC analyses were performed, and the percentage of positively stained cells was quantified and statistically analyzed as previously described. Briefly, based on the IHC scores, we divided the patients into the negative-staining group (0–25%), the weak-staining group (25–50%), the moderate-staining group (50–75%), and the strong-staining group (75–100%). The high-expression group was composed of patients with tumors exhibiting moderate or strong staining intensity, while the low-expression group was composed of patients with tumors exhibiting negative or weak staining intensity.

### RNA extraction and real-time PCR

Total RNA was extracted using TRIzol reagent (Invitrogen, Carlsbad, USA) following the manufacturer’s instructions. Total RNA (1 μg) was reverse-transcribed into cDNA using a PrimeScript RT Kit (Takara Bio Inc., Japan). Quantitative reverse transcription-PCR was performed using a SYBR Green Kit (Takara Bio Inc., Japan). β-Actin mRNA was employed as a reference for normalization of expression, and the 2−ΔΔCt method was applied to quantify changes in the expression of target mRNAs within samples. The primers used in this study are shown in Additional file 3.

### Transmission electron microscopy

For TEM, cells were fixed with 3% glutaraldehyde in 0.1 M phosphate buffer (pH 7.4) and then fixed with 1% OsO_4_. After dehydration, 10-nm thin sections were prepared and stained with uranyl acetate and lead (II) nitrate before examination under a JEM-1230 transmission electron microscope (JEOL, Tokyo, Japan). High-resolution digital images were acquired from 10 randomly selected fields for samples of each condition.

### Autophagy induction and ad-mCherry-GFP-LC3 transient transfection

For autophagy analysis, HCC cells with or without PHF8 knockdown, PHF8 overexpression or combined PHF8 knockdown, and FIP200 exogenous overexpression were infected with adenoviruses expressing the Ad-mCherry-GFP-LC3 fusion protein (Vigene Bioscience, at a multiplicity of infection of 20). After incubation in a complete medium for 48 h, the cells were treated with starvation medium (EBSS) for 6 h and observed under a fluorescence microscope. Autophagic flux was assessed by manually counting the yellow and red dots in each cell in five random fields from images in which the red and green channels were merged. The yellow and red dots represented autophagosomes and autolysosomes, respectively.

### Chromatin immunoprecipitation

ChIP was carried out with a SimpleChIP® Enzymatic Chromatin IP Kit (Magnetic Beads) (9003, Cell Signaling Technology) according to the manufacturer’s specifications. MHCC-LM3 and HEK293 cells were cultured in 10 cm dishes. DNA and proteins in cells were crosslinked with 1% paraformaldehyde/PBS at room temperature for 10 min; the reaction was terminated with 0.125 M glycine. After the cells were harvested and lysed as described in the manufacturer’s instructions, lysates from the two groups were incubated with 4 μg of anti-E2F1 antibody (ab128874, Abcam), normal rabbit IgG (ChIP kit) or anti-RNA polymerase II antibody (ChIP kit) overnight at 4 °C. The antibody–protein complexes were precipitated using magnetic beads (ChIP kit) at 4 °C for 2 h and eluted with IP elution buffer at 65 °C for 30 min. The proteins were digested with proteinase K and reverse-crosslinked at 65 °C for 90 min. DNA was removed with a DNA clean-up column (ChIP kit) and resuspended in 50 μl of DNA elution buffer. The primers used for ChIP are shown in Additional file 3.

### Luciferase assay

Wild-type and mutant DNA fragments corresponding to the open reading frame of the human NOP gene were subcloned into the pGL3-Basic vector (denoted P1, P2, and P3). All constructs were confirmed by DNA sequencing. Cells were plated at a density of 1.2 × 10^5^ cells/well in 96-well plates prior to transfection. MHCC-LM3 and HEK293 cells were transfected with luciferase plasmids along with a plasmid containing the Renilla luciferase gene and the NOP expression plasmid using Lipofectamine™ 2000 Reagent (Invitrogen). Luciferase activity was measured with a Dual-Luciferase Assay System (Promega) 48 h post-transfection. The ratio of firefly luciferase activity to Renilla luciferase activity was considered the relative luciferase activity, which was then normalized to that of the pGL3-Basic vector.

### Plasmids, shRNAs, and lentiviruses

Lentiviral shRNAs targeting NOP with nonoverlapping sequences were purchased from GeneChem (Shanghai, China). The promoter sequence of NOP was cloned from genomic DNA extracted from Huh7 cells and was then subcloned into the pGL3-Basic luciferase reporter vector. Site-directed mutagenesis of the NOP promoter and the corresponding plasmid was performed by GeneChem (Shanghai, China). The lentiviruses described above were added directly to cells for 24 h, and the cells were then cultured in the presence of puromycin (1.0 μg/ml, Sigma–Aldrich) for selection of stably transduced cells. The corresponding protein knockdown or overexpression in the cell lines was confirmed by western blotting. Control cells were transduced with lentiviral vectors containing scrambled control shRNA (GeneChem, China) accordingly.

### Xenograft assay

MHCC-LM3 vector or MHCC-LM3 NOP cells (1 × 10^7^) in the exponential growth phase were subcutaneously inoculated into the thighs of 5-week-old male BALB/c nude mice (GemPharmatech Co. Ltd, China). Huh7 shNC or Huh7 shNOP cells (1 × 10^7^) in the exponential growth phase were subcutaneously inoculated into the thighs of 5-week-old male BALB/c-nude mice. The tumor volume was calculated with the following formula: Volume = 0.5 × Length × Width^2^ (mm^3^). When the tumor volume was 200 mm^3^, the mice were randomly assigned to independent groups (six mice per group) as follows: shNC + NS, shNC + nociceptin, shNC + JTC801, shNOP + DMSO, shNOP + nociceptin, and shNOP + JTC801. After grouping, JTC801 and nociceptin were intraperitoneally administered at a dose of 50 mg/kg once a day [14]. The in vivo experiments were approved by the Institutional Animal Care and Use Committee of Sun Yat-sen University and abided by the Institutional Guidelines and Protocols.

### Statistical analysis

The data were analyzed using SPSS software 25.0 (IBM, SPSS, Chicago, USA) and processed with GraphPad Prism 8.0 (GraphPad Prism, CA, USA) and are presented as the means ± SDs. Student’s *t* test or ANOVA was used for group comparisons. Relapse-free survival and overall survival curves were plotted via the Kaplan–Meier method and compared by the log-rank test. A *p* value <0.05 (two-tailed) was considered to indicate statistical significance. The expression level of NOP was dichotomized for OS analysis followed by the log-rank test according to optimal cutoff values calculated with the “survcutpoint” function of the “survminer” R package.

## Supplementary information

original western blots

final supplementary material

SuppleFig1

SuppleFig2

SuppleFig3

## Data Availability

The original contributions presented in the study are included in the paper. The full-length uncropped original western blots are included in Supplementary Material. Further inquiries can be directed to the corresponding authors.
