# Supplementary material for: The nociceptin receptor promotes autophagy through NF-kB signaling and is transcriptionally regulated by E2F1 in HCC
Source: Cell Death Discov. 2022 Apr 5;8:165. doi: 10.1038/s41420-022-00978-7 (PMC8983730; doi:10.1038/s41420-022-00978-7)
Supplement: Supplementary file 1 — original western blots [file 41420_2022_978_MOESM1_ESM.docx]

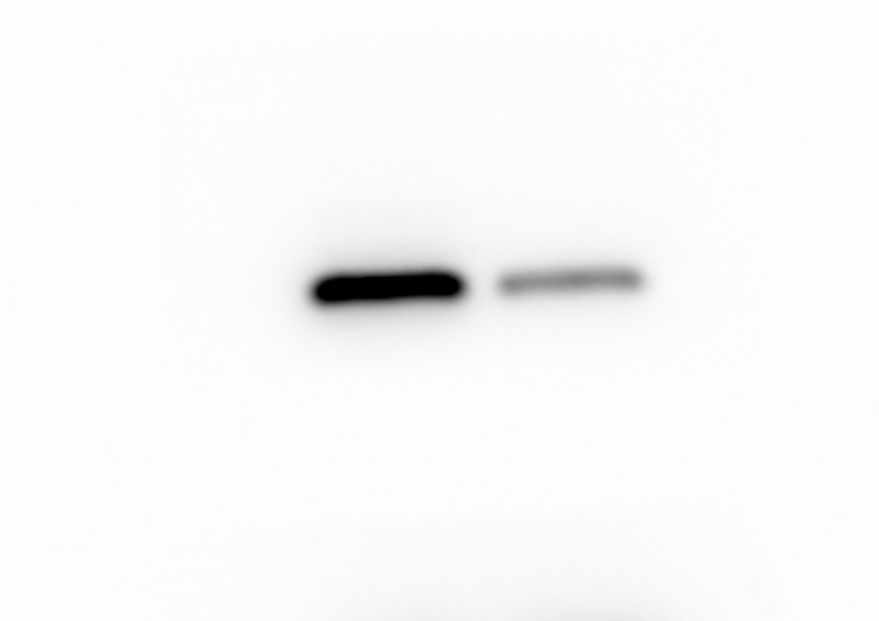
Fig3C BAX (left)


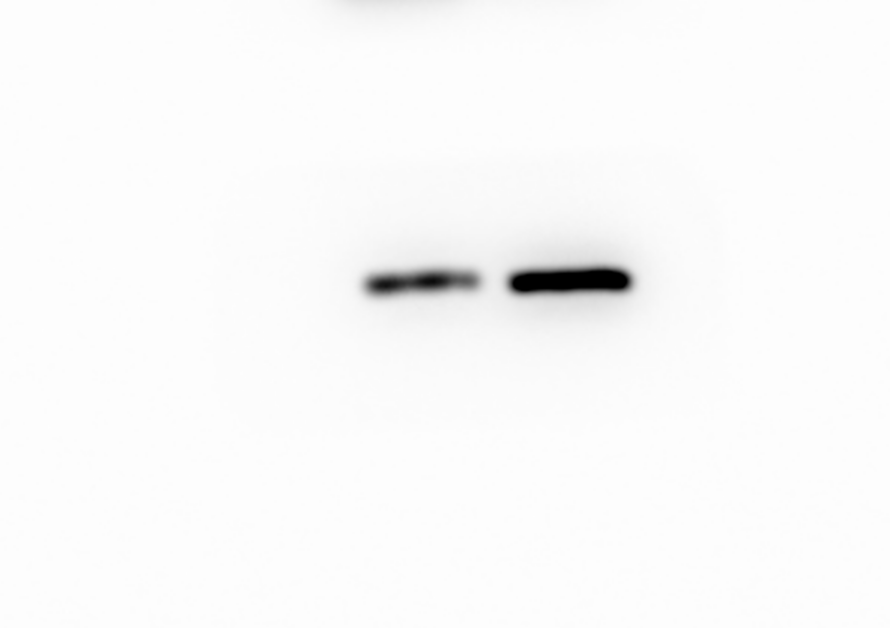
Fig3C Bcl-2 (left)


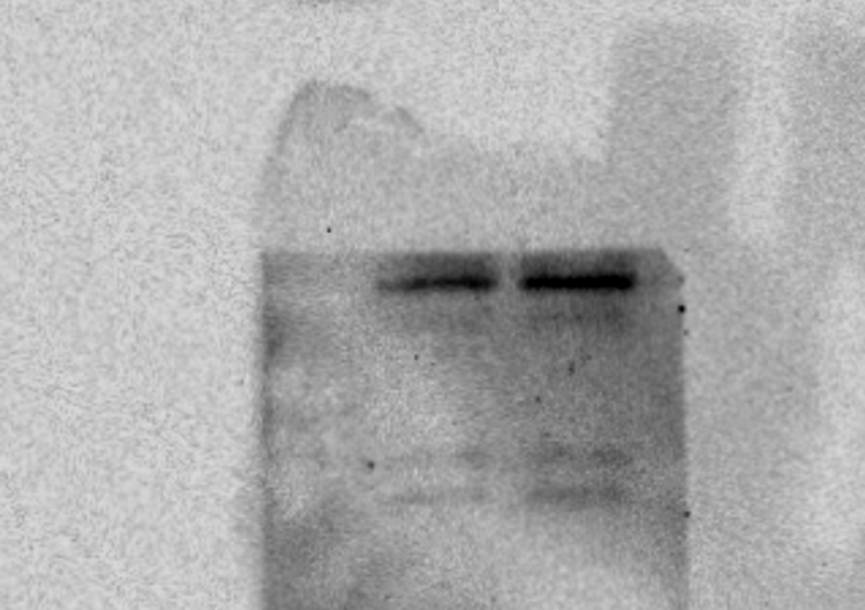
Fig3C. Caspase-3 (left)


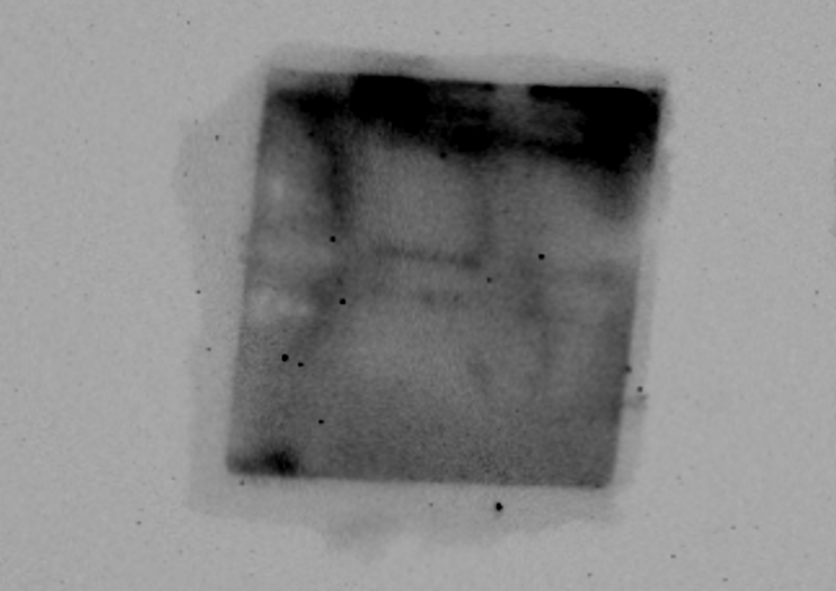
Fig3C. Cleaved caspase-3 (left)


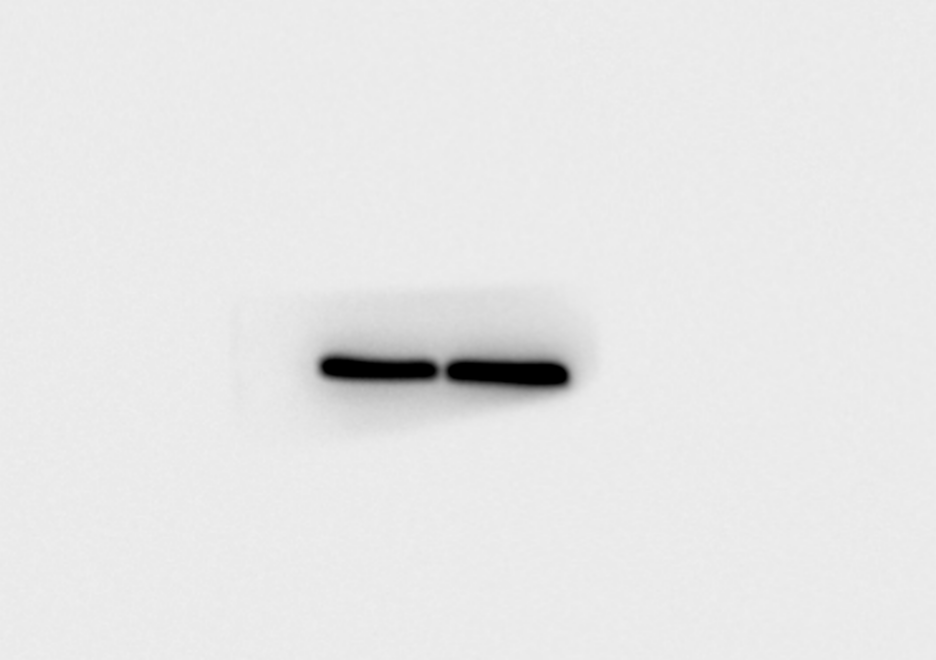
Fig3C. GAPDH (left)


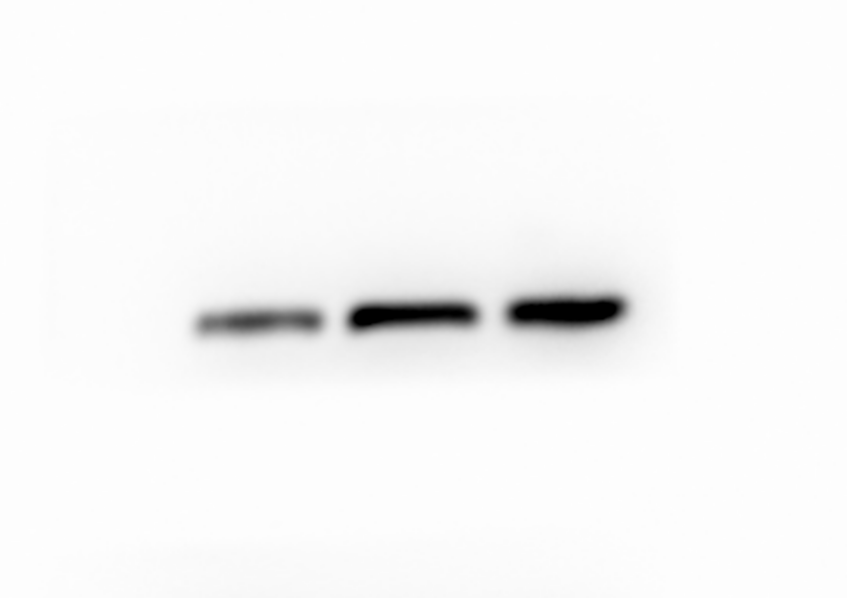
Fig3C. BAX (right)


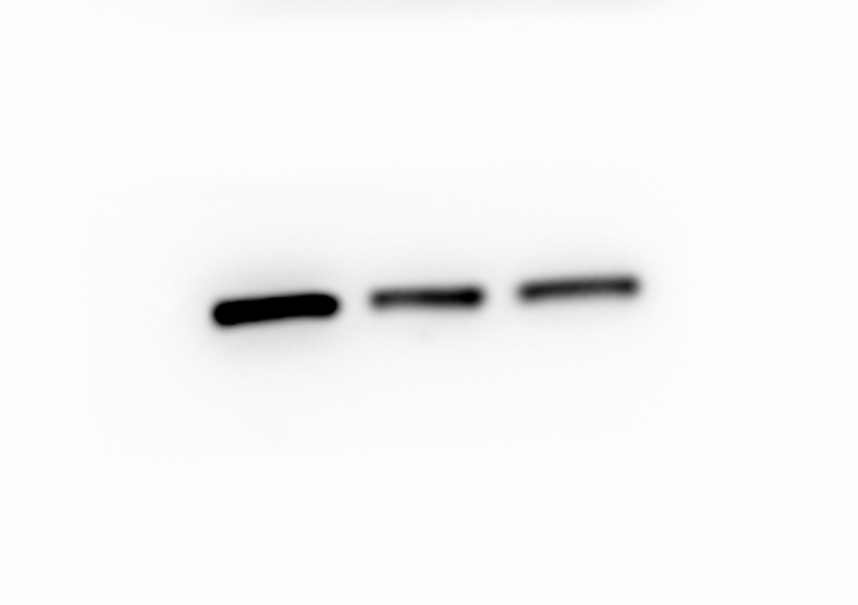
Fig3C. Bcl-2 (right)


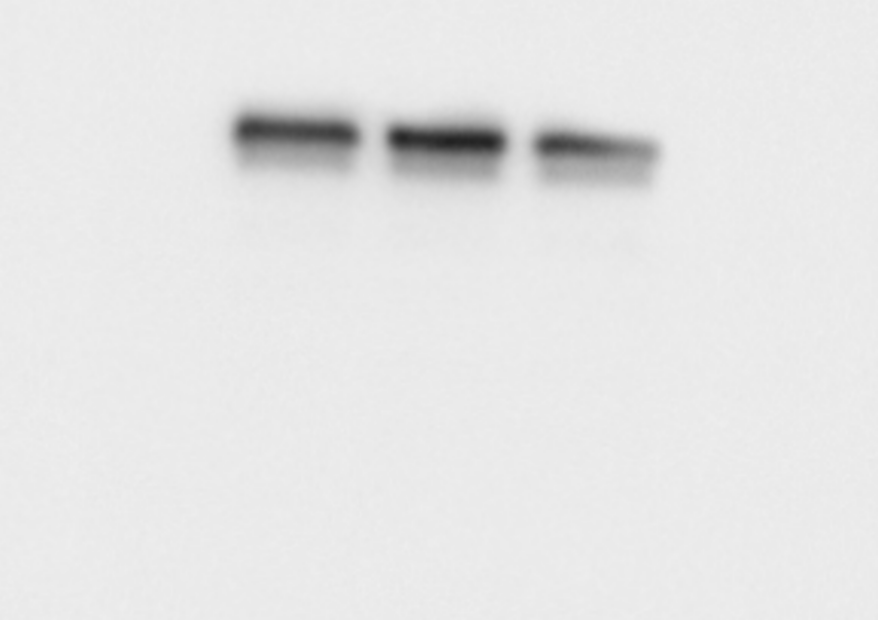
Fig3C. caspase-3 (right)


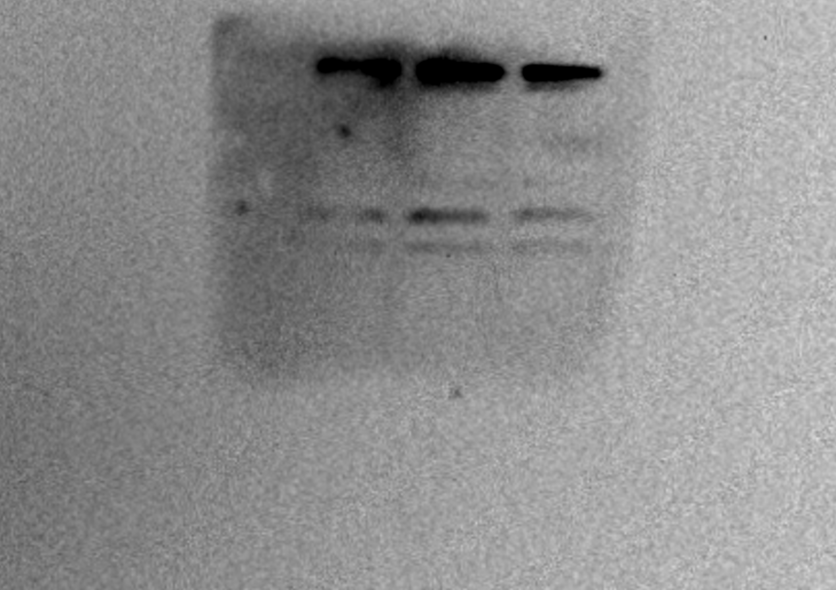
Fig3C. cleaved-caspase-3 (right)


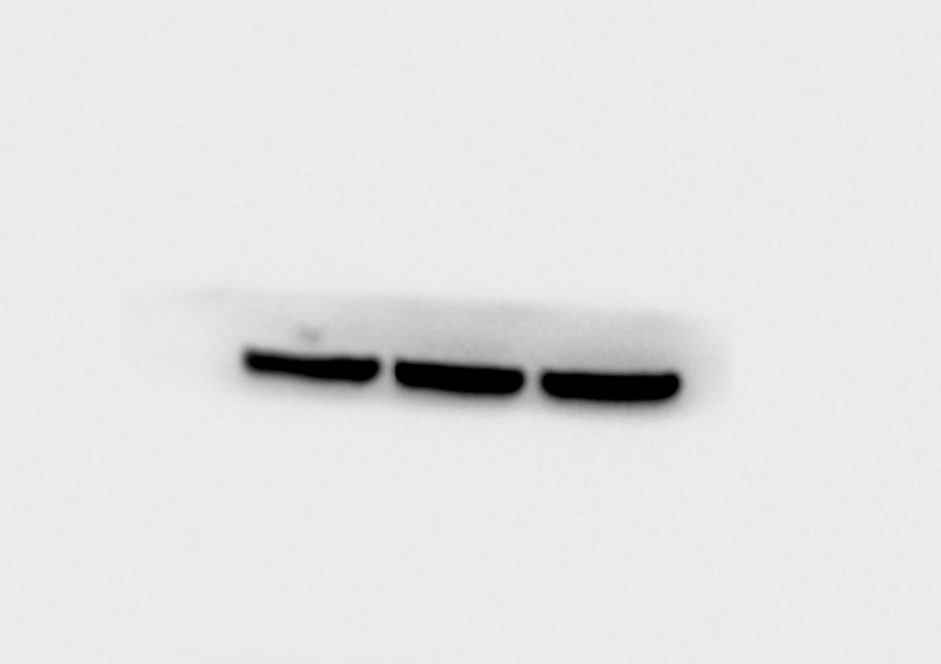
Fig3C. GAPDH (right)


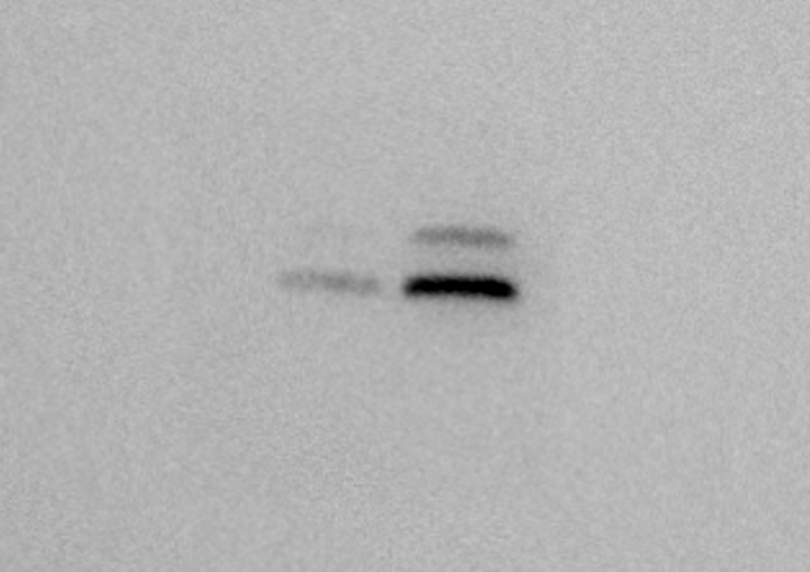
Fig3D. LC3 (left)


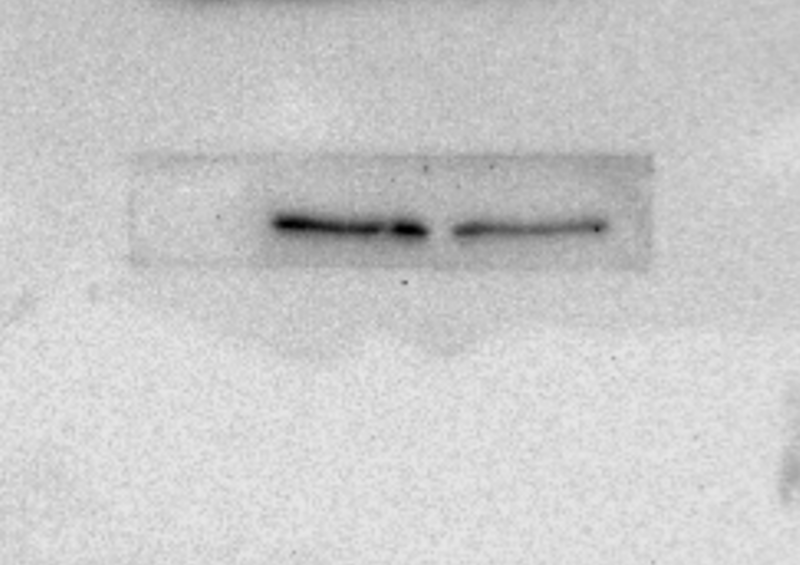
Fig3D. p62(left)


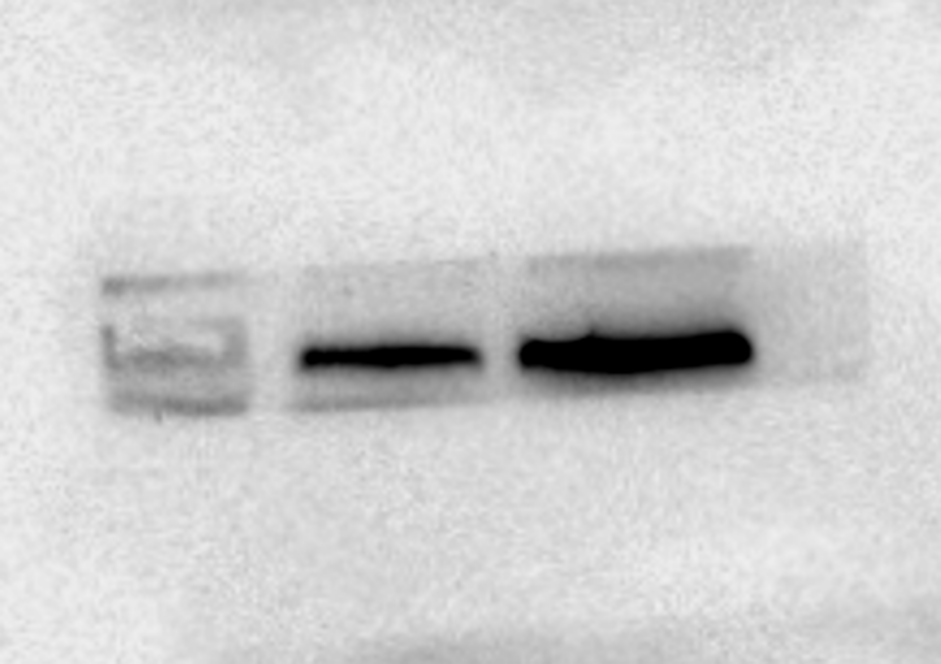
Fig3D. Beclin-1 (left)


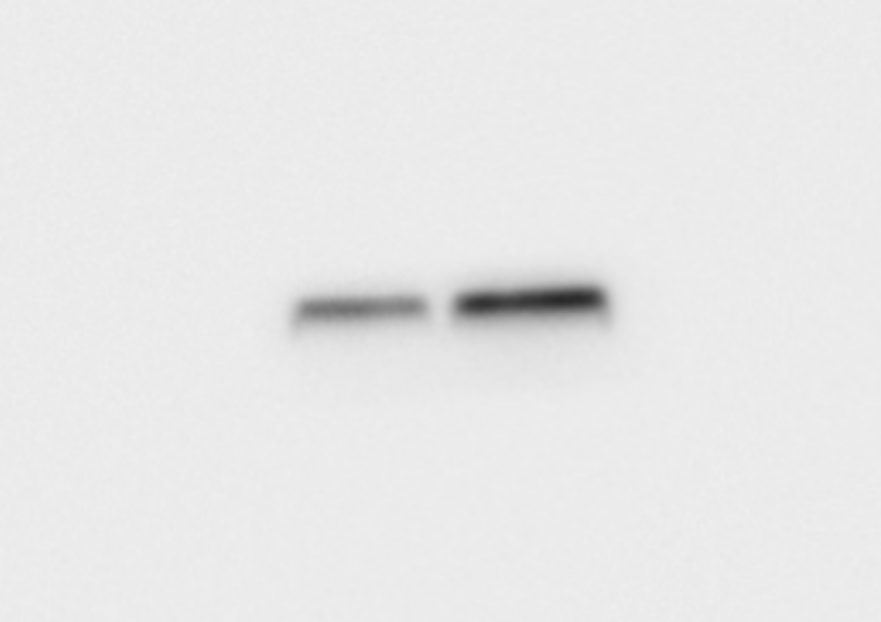
Fig3D. atg5 (left)


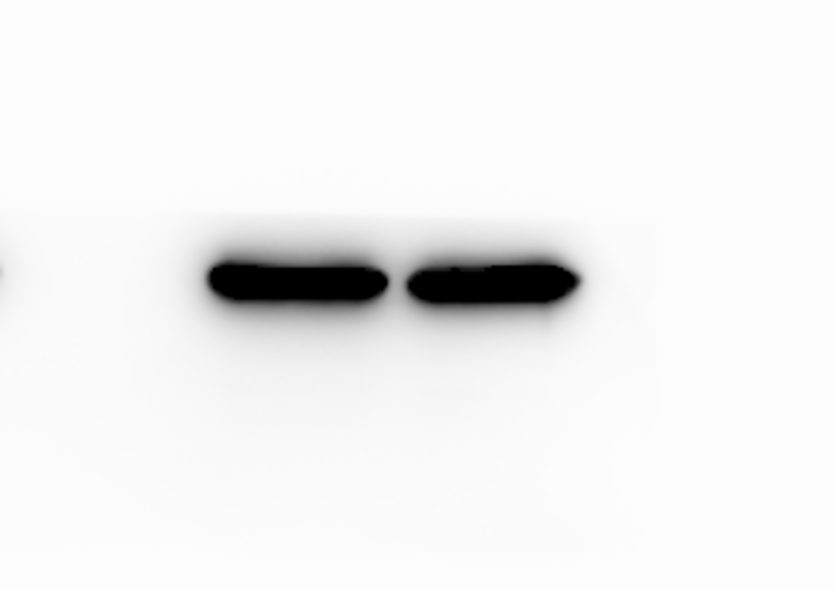
Fig3D. GAPDH (left)


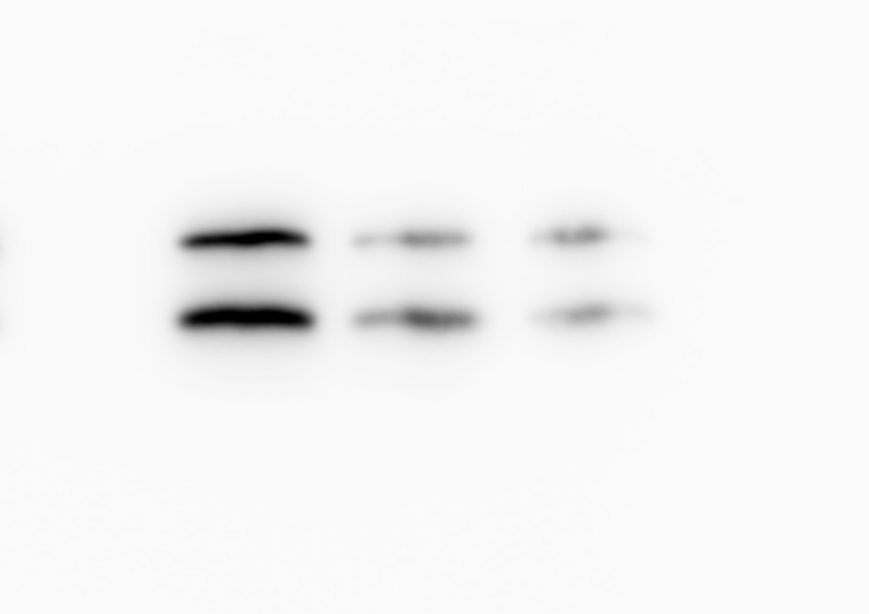
Fig3D. LC3 (right)


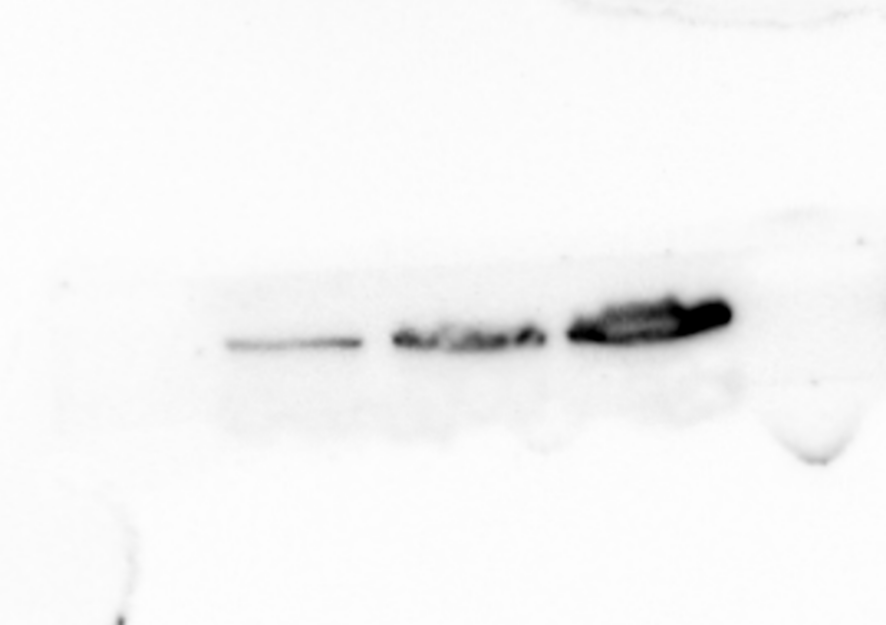
Fig3D. p62 (right)


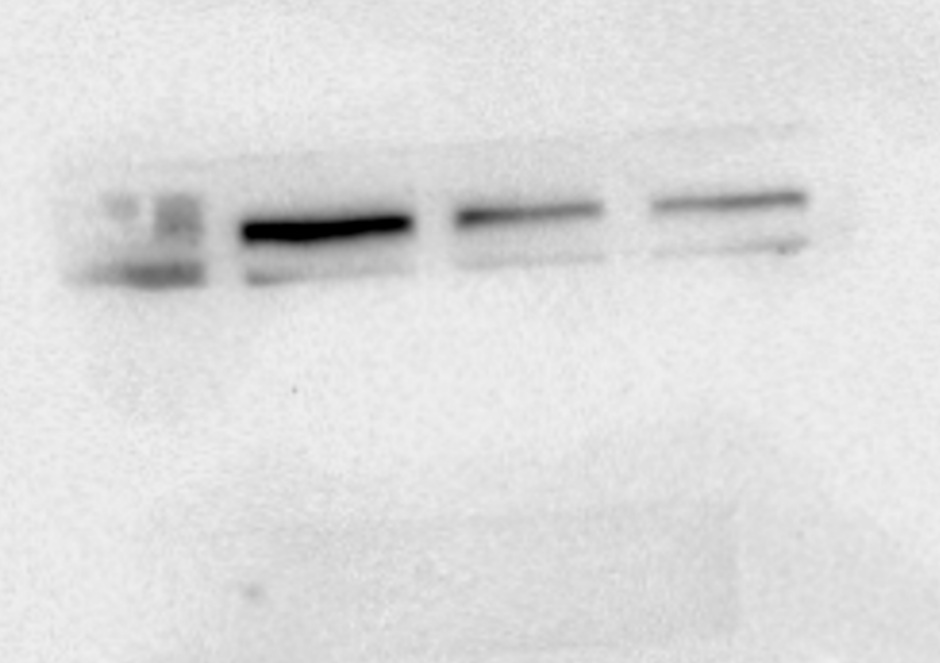
Fig3D. beclin-1 (right)


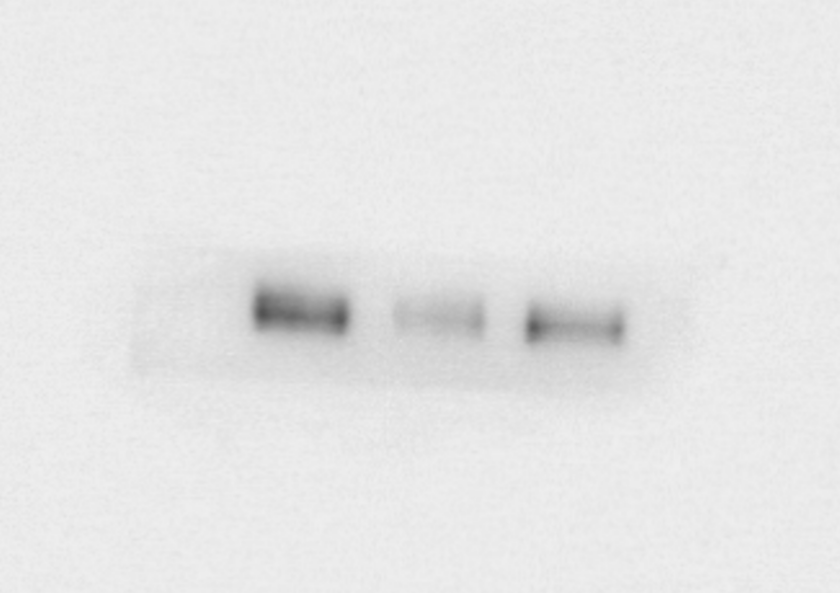
Fig3D. atg5 (right)


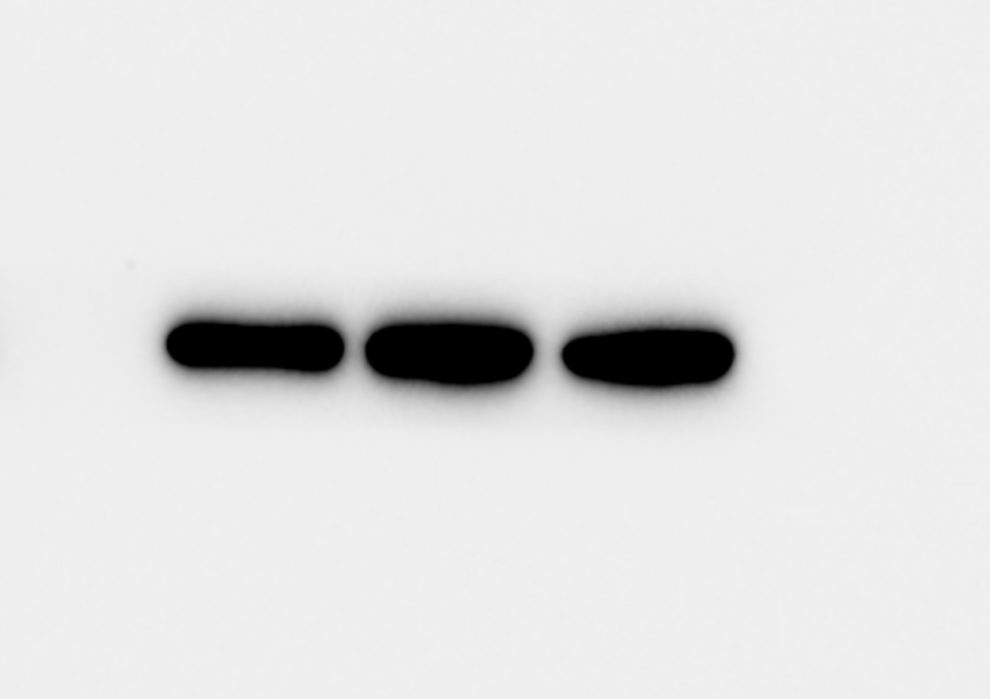
Fig3D. GAPDH(right)


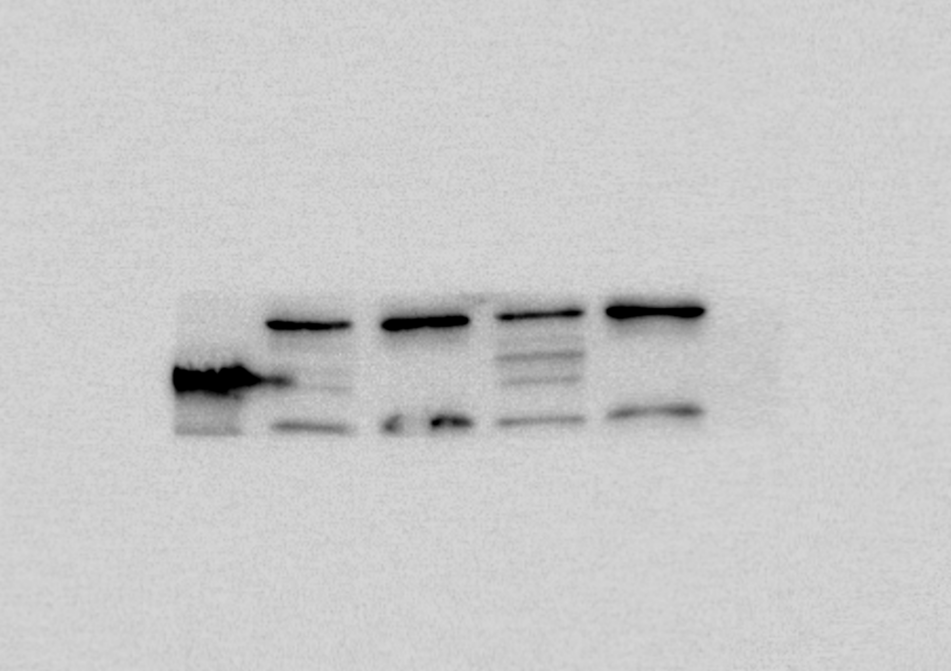
Fig3H. NOP


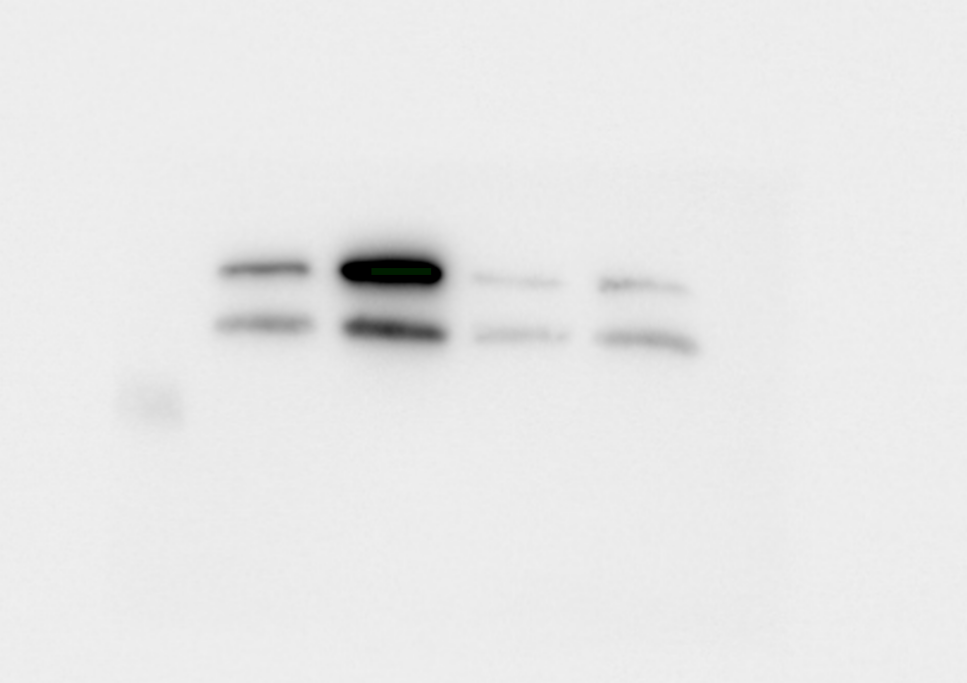
Fig3H. LC3


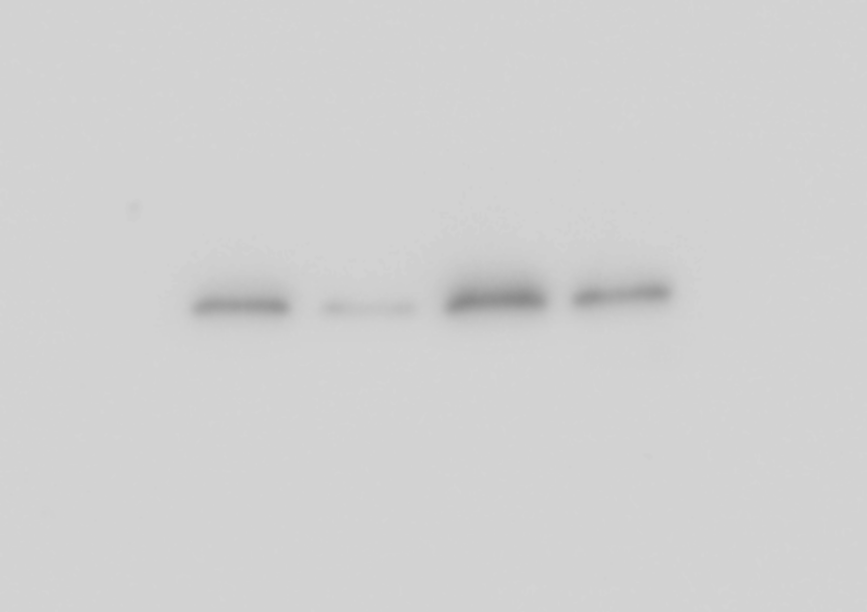
Fig3H. p62


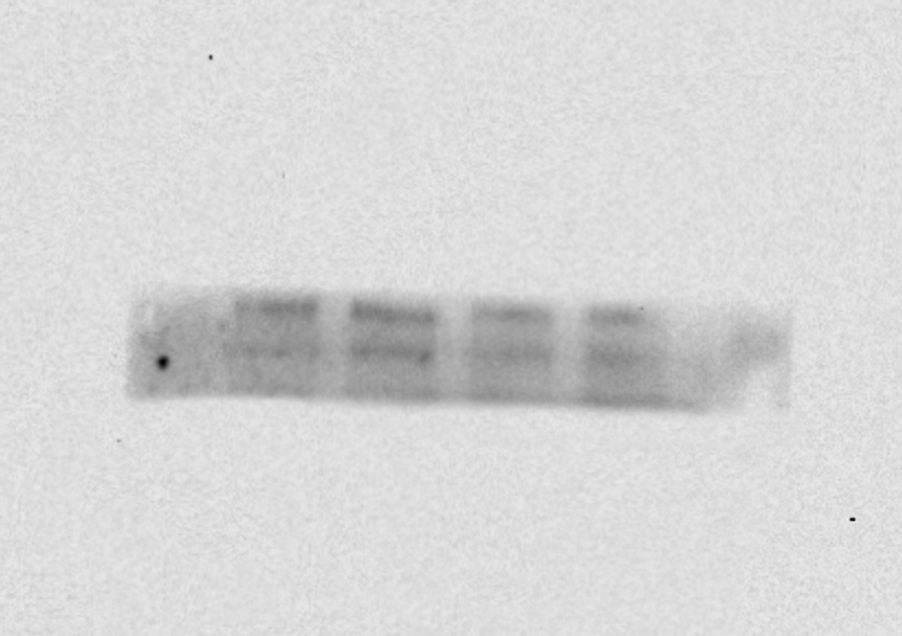
Fig3H. caspase-3


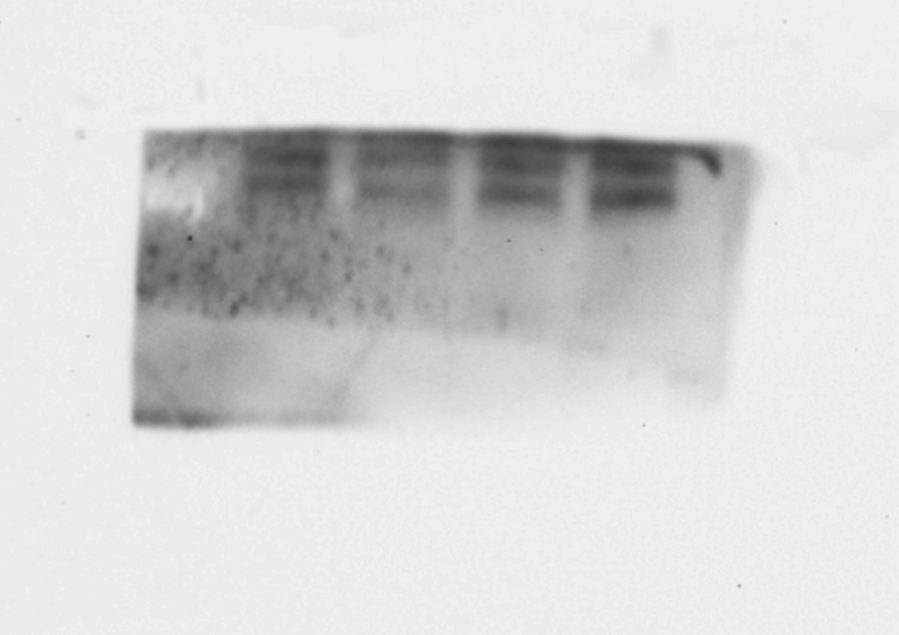
Fig3H. cleaved caspase-3


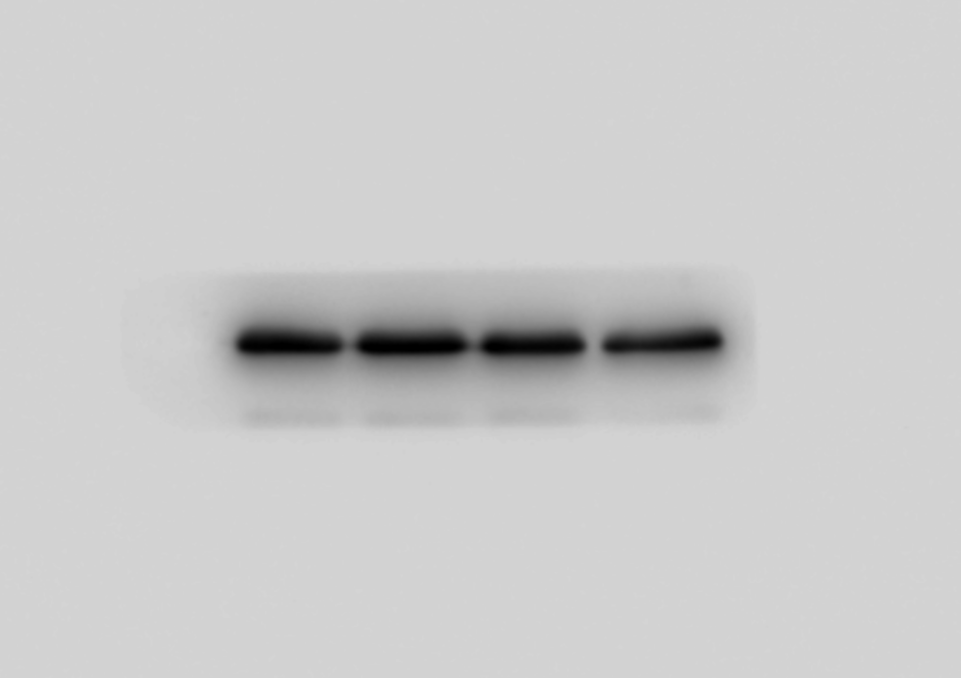
Fig3H. GAPDH


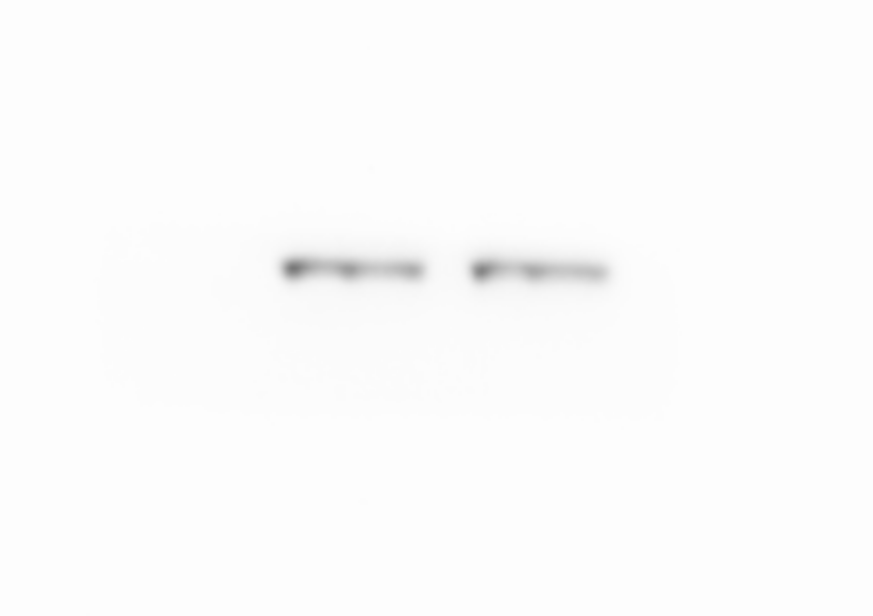
Fig4A. p65(left)


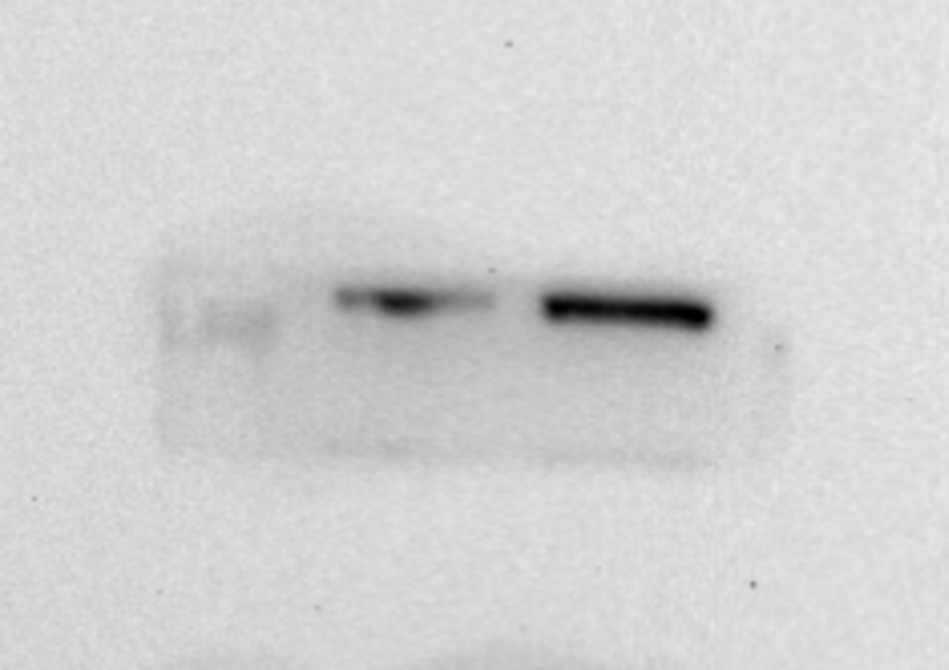
Fig4A. p-p65(left)


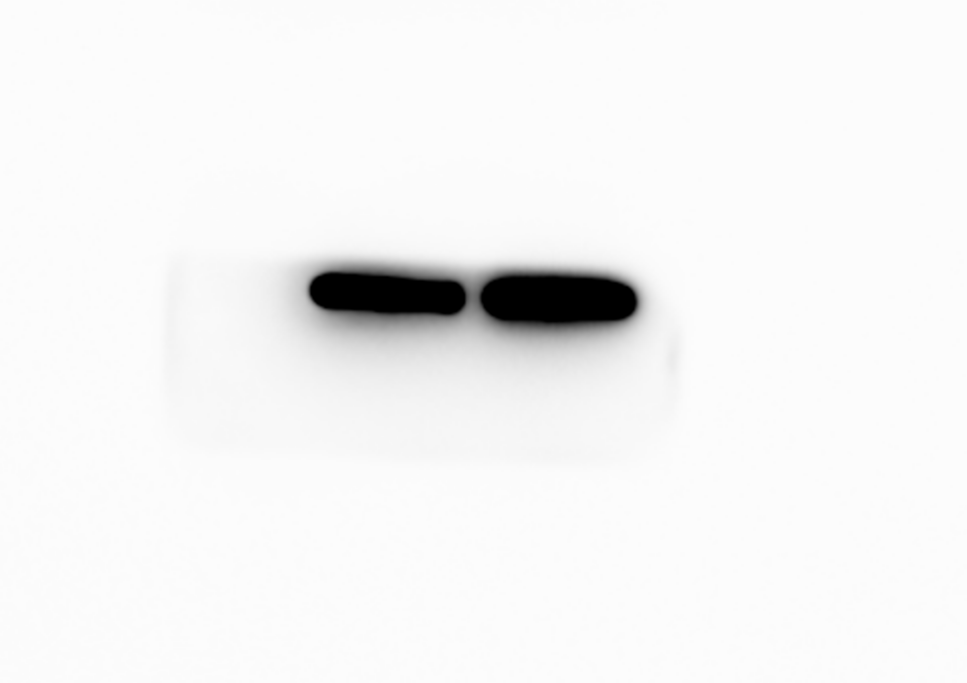
Fig4A. GAPDH(left)


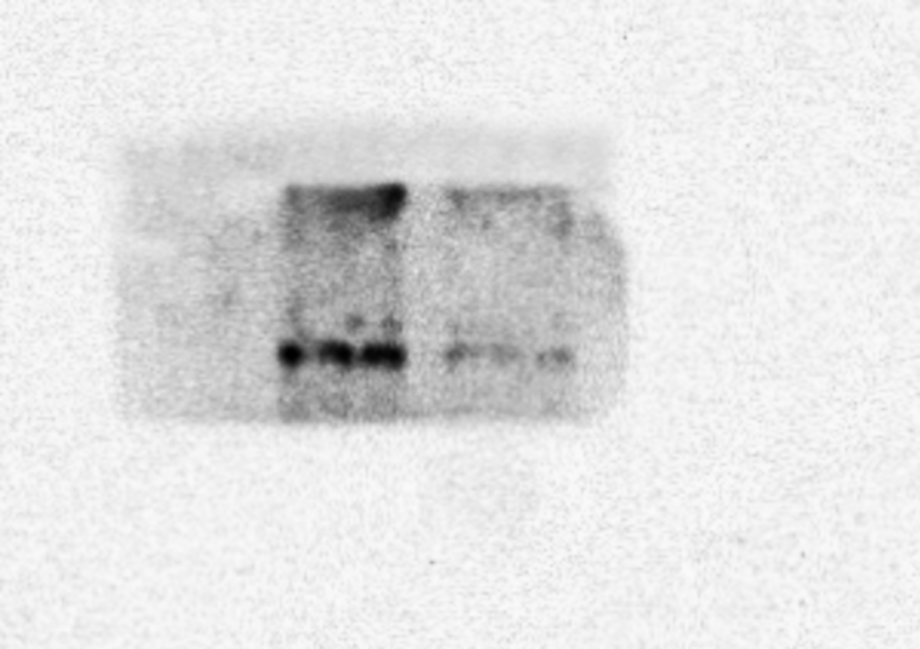
Fig4A. cyto p-p65(left)


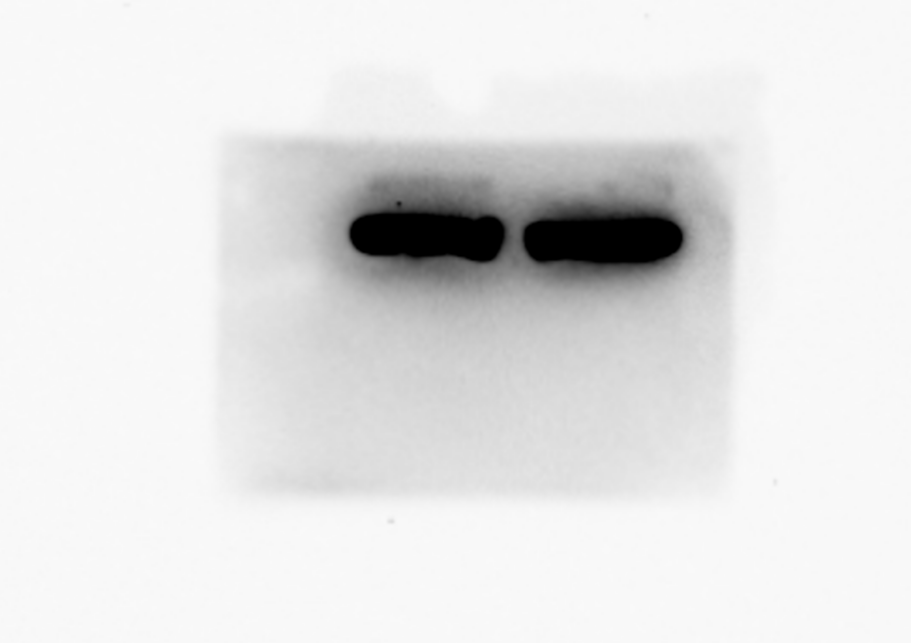
Fig4A. cyto GAPDH(left)


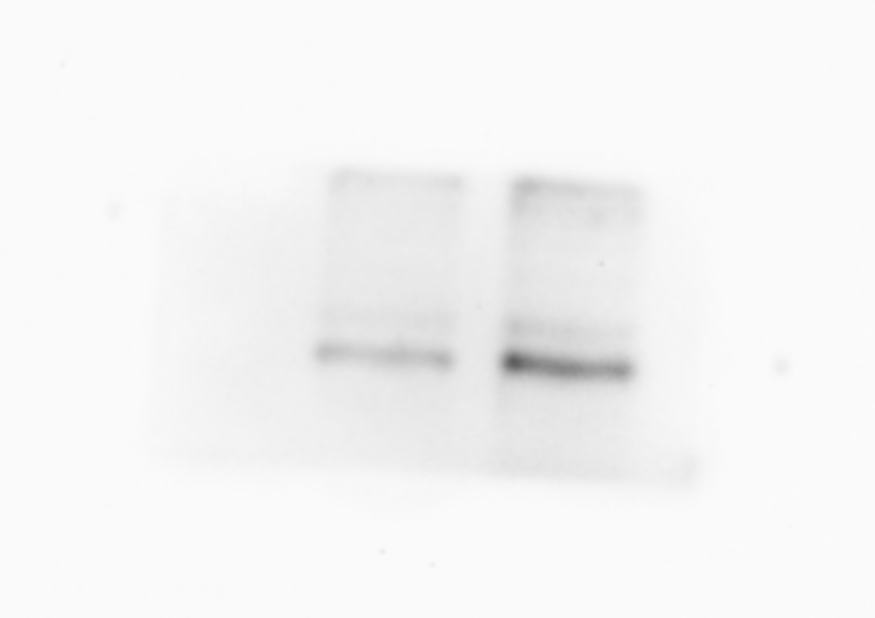
Fig4A. Nuc p-p65 (left)


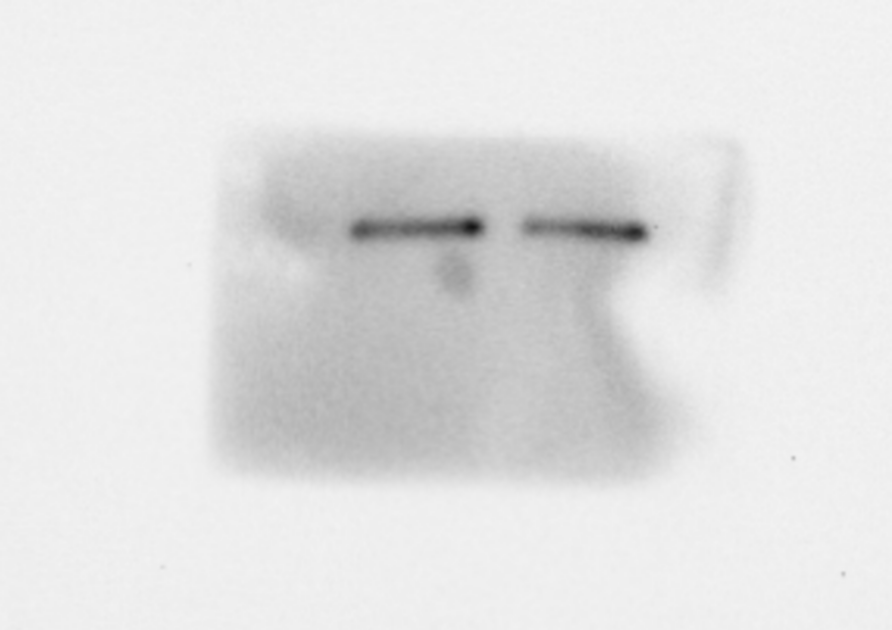
Fig4A. Nuc Histone H3(left)


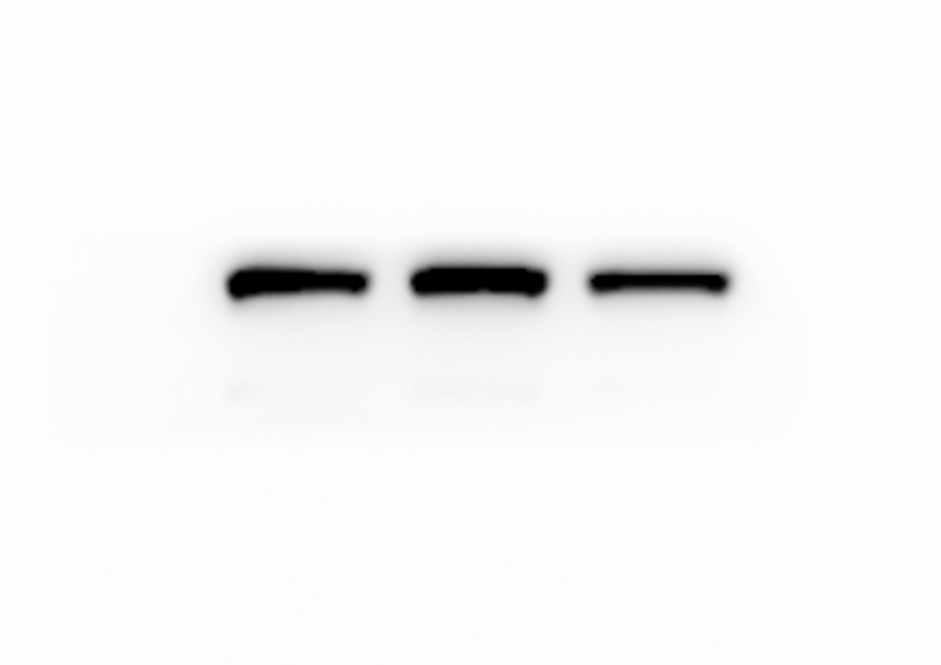
Fig4A. p65 (right)


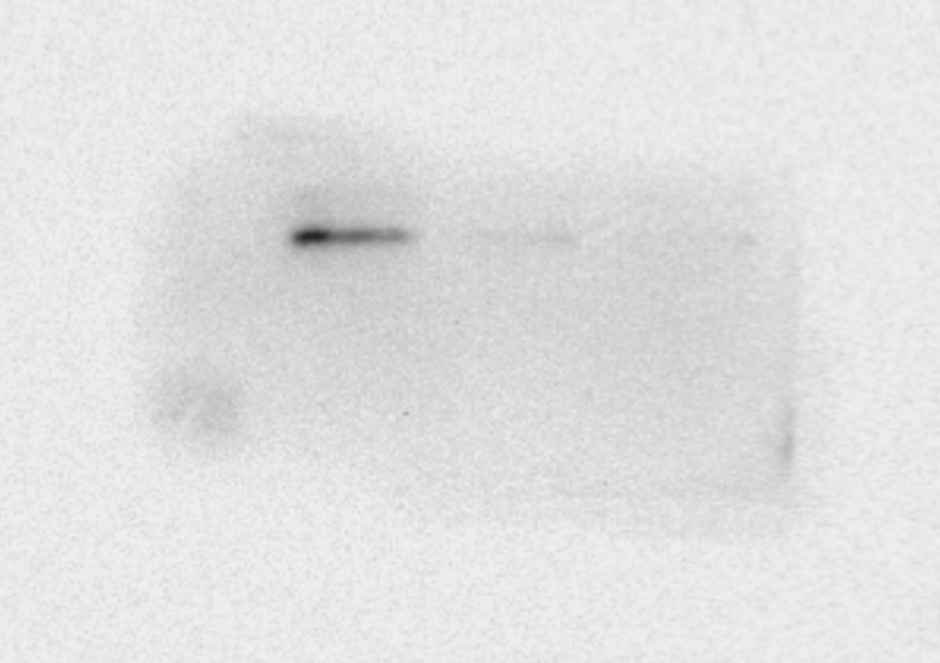
Fig4A. p-p65 (right)


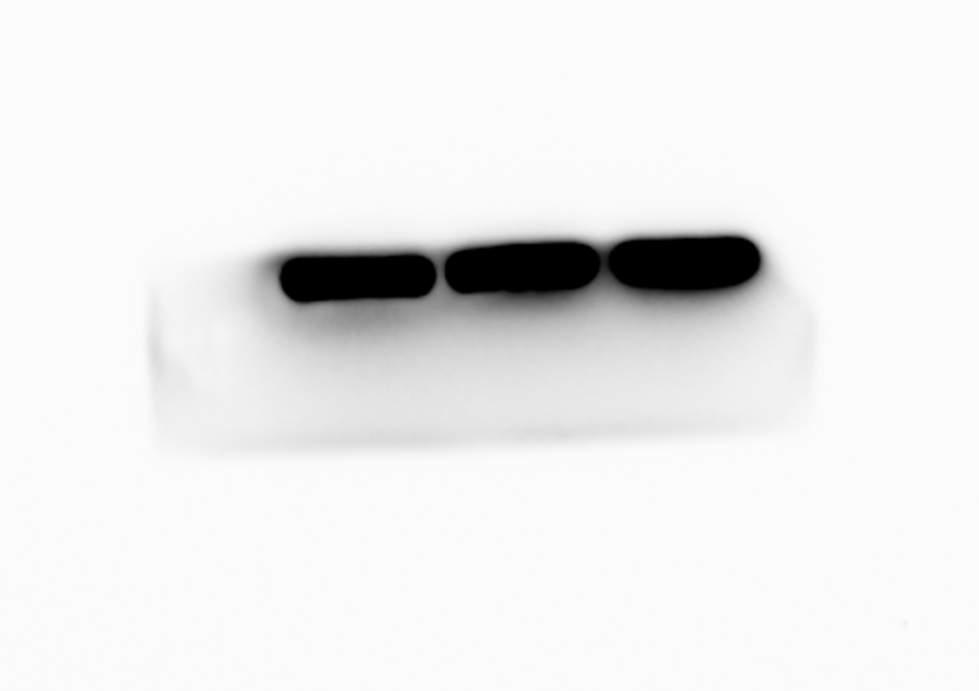
Fig4A. GAPDH (right)


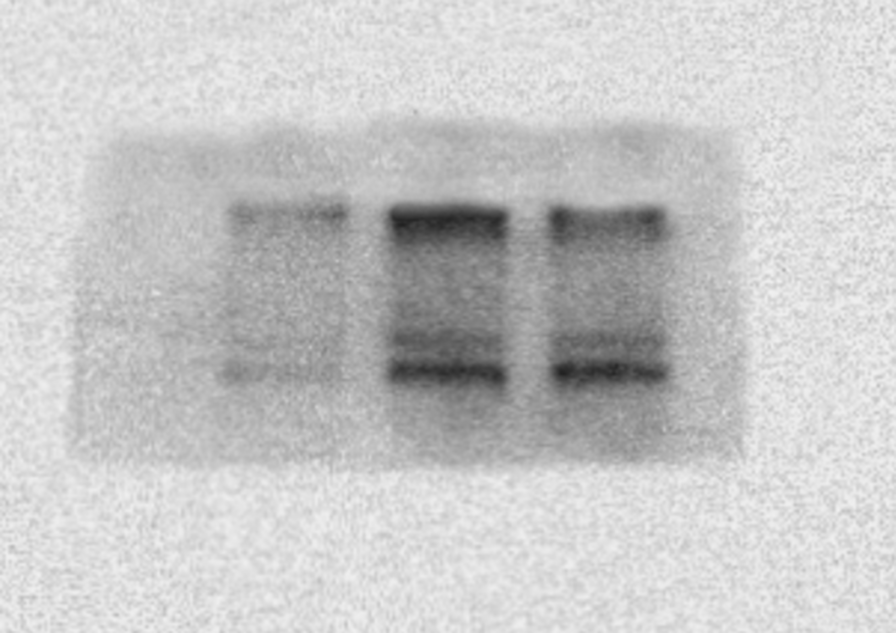
Fig4A. cyto p-p65 (right)


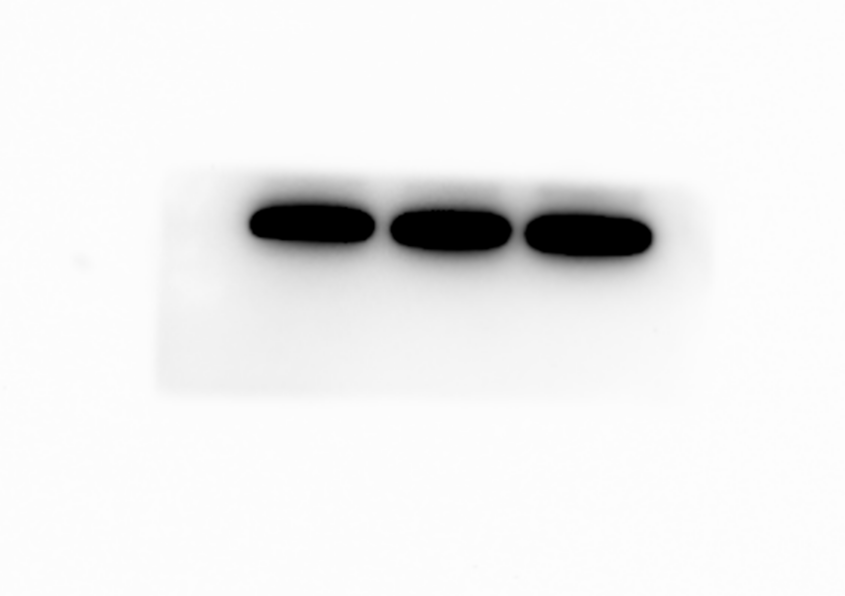
Fig4A. cyto GAPDH (right)


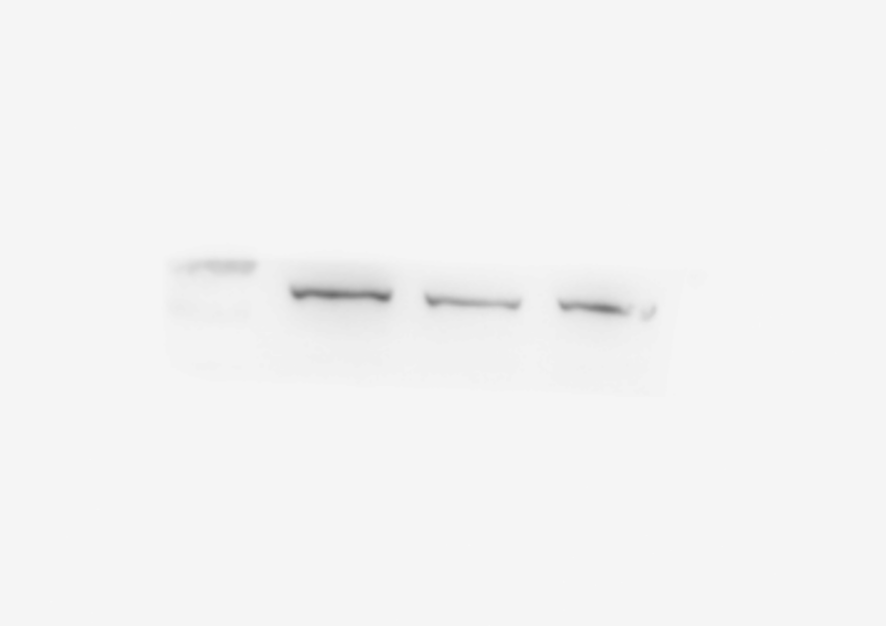
Fig4A. Nuc p-p65 (right)


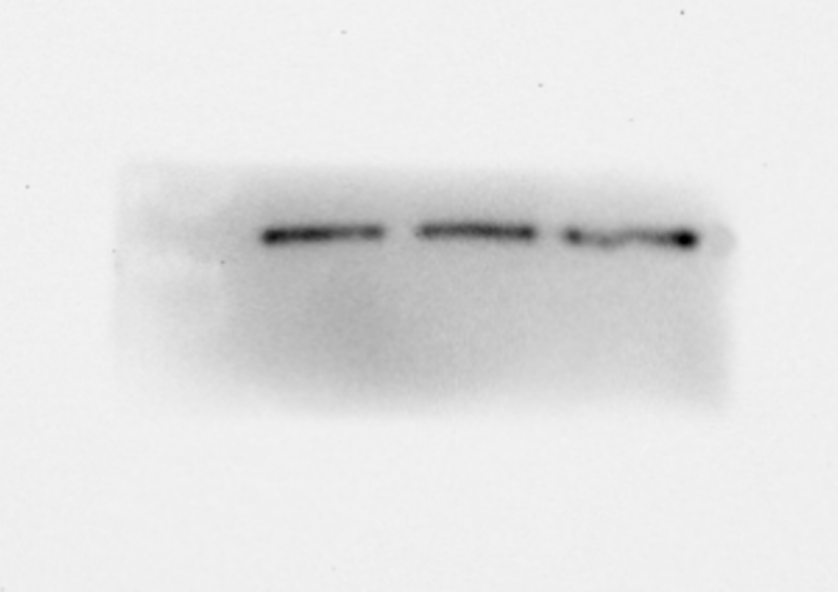
Fig4A. Nuc Histone H3 (right)


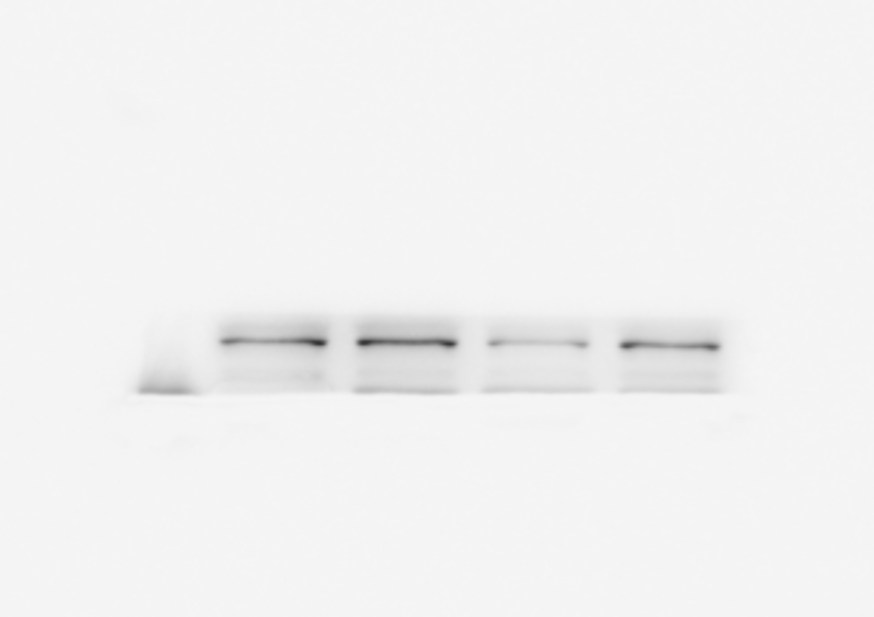
Fig4B. NOP


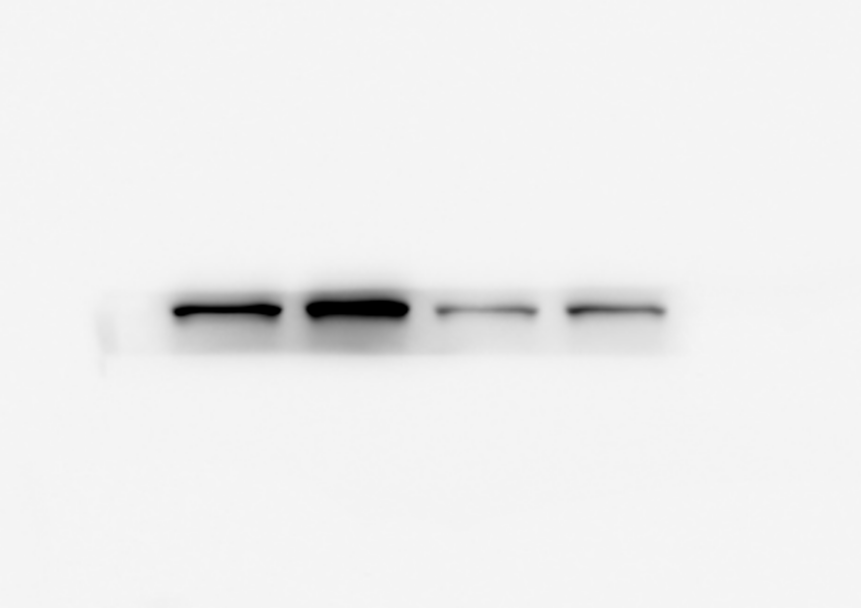
Fig4B. p-p65


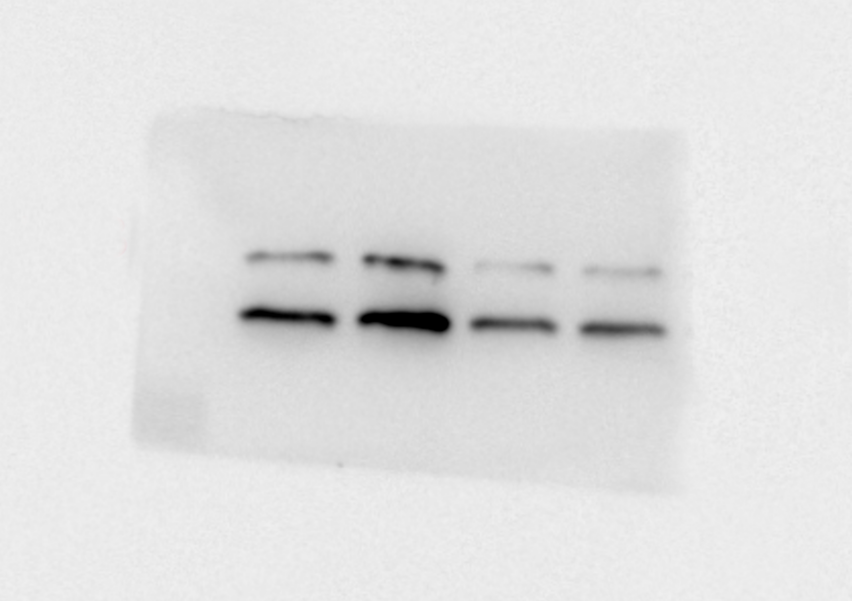
Fig4B. LC3


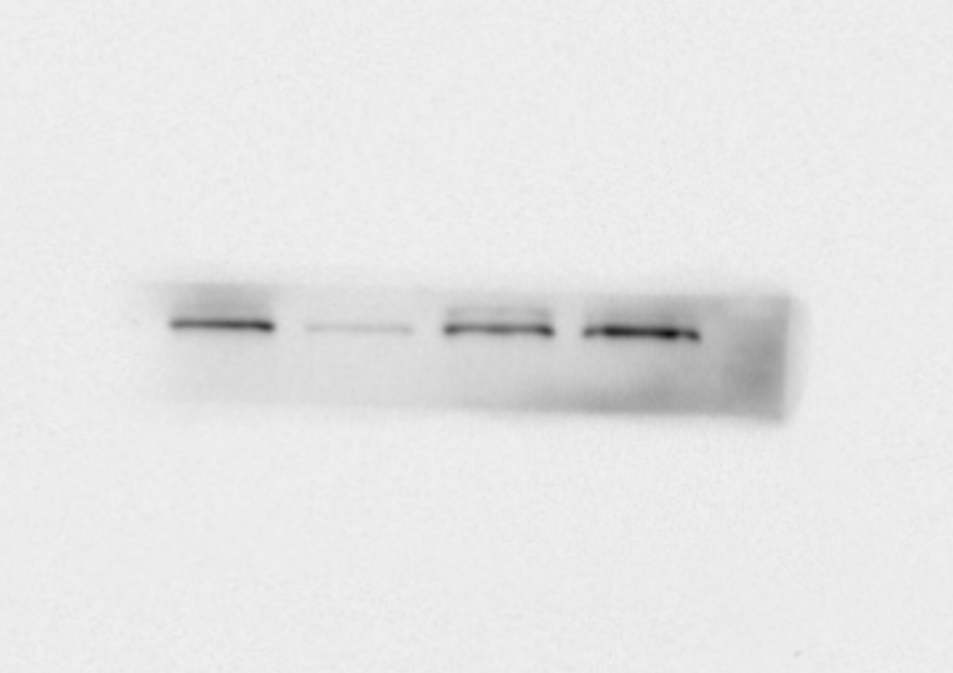
Fig4B. p62


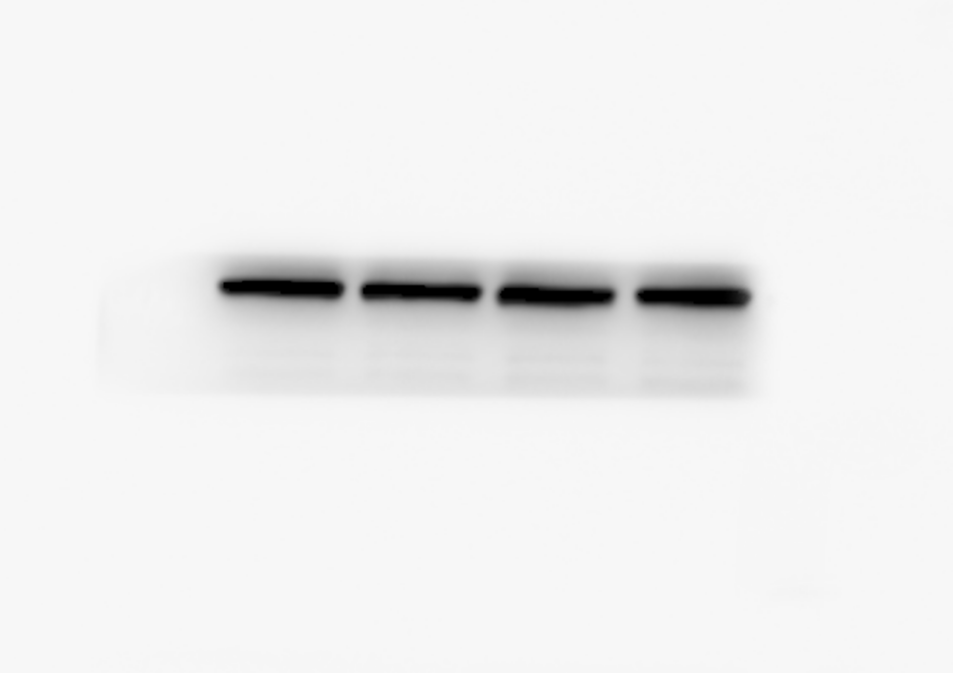
Fig4B. GAPDH


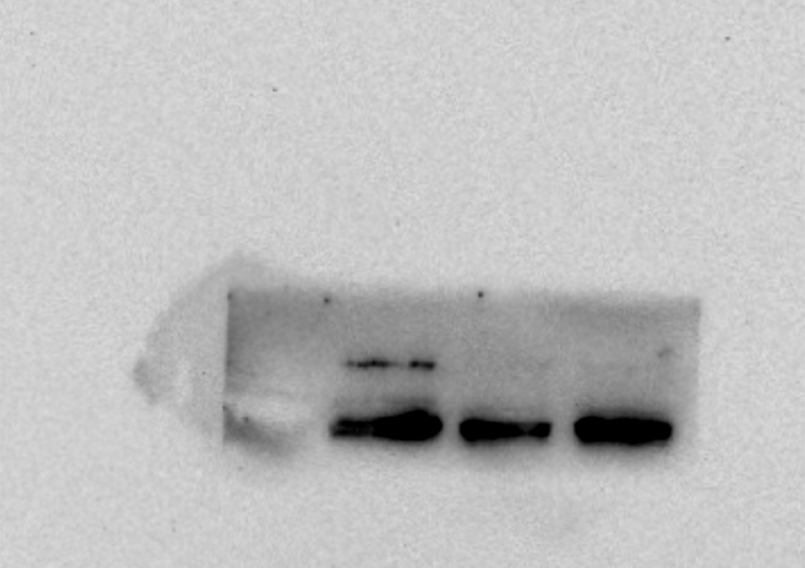
Fig5D. E2F1 (left)


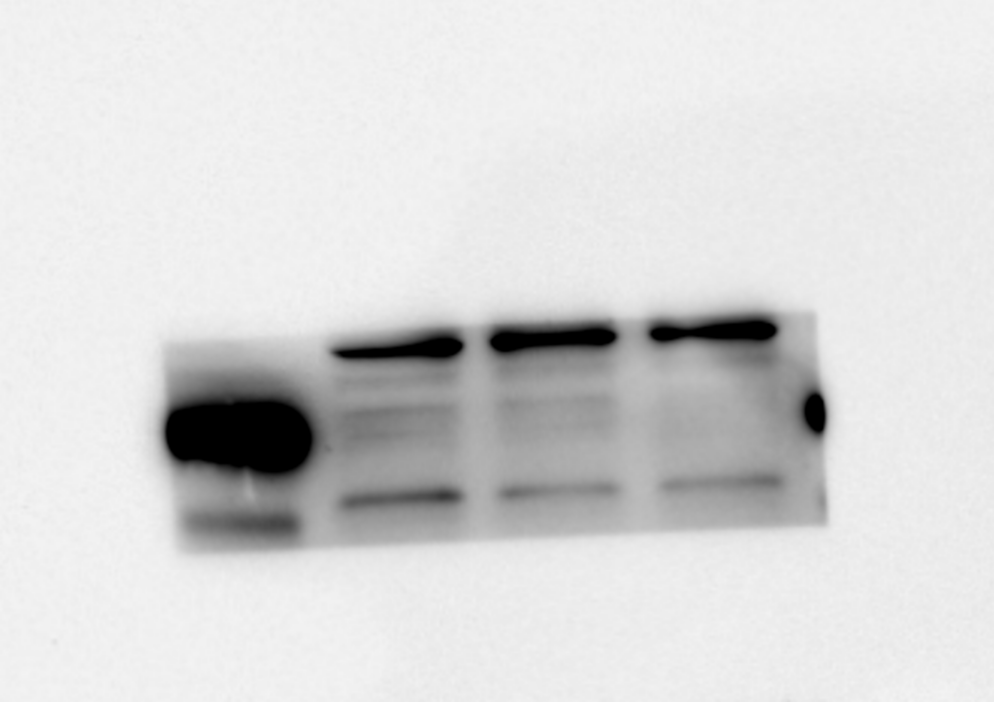
Fig5D. NOP (left)


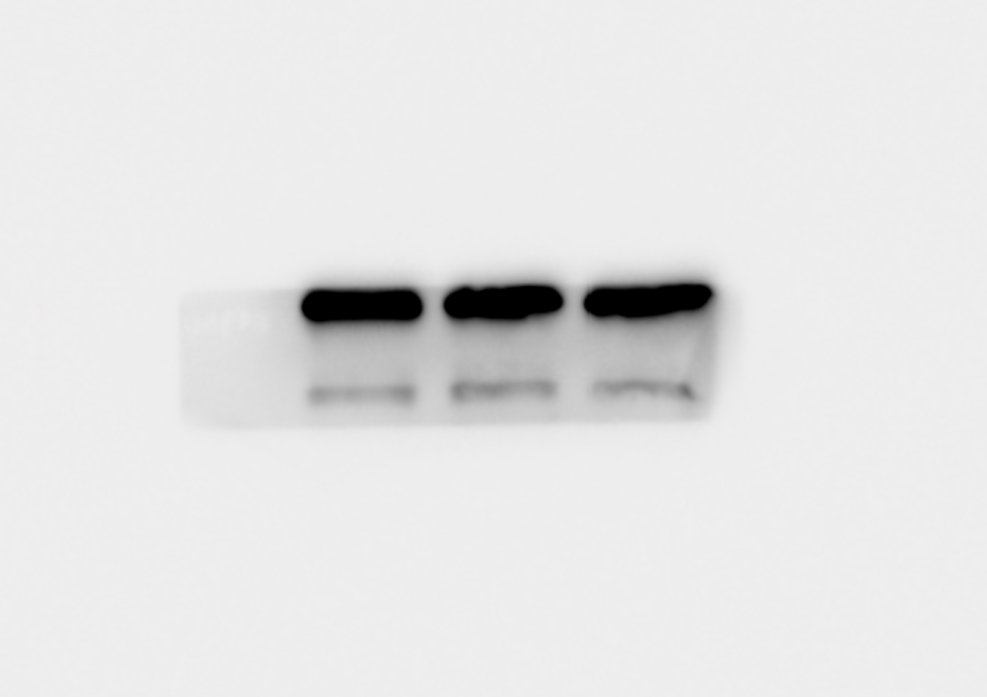
Fig5D. GAPDH (left)


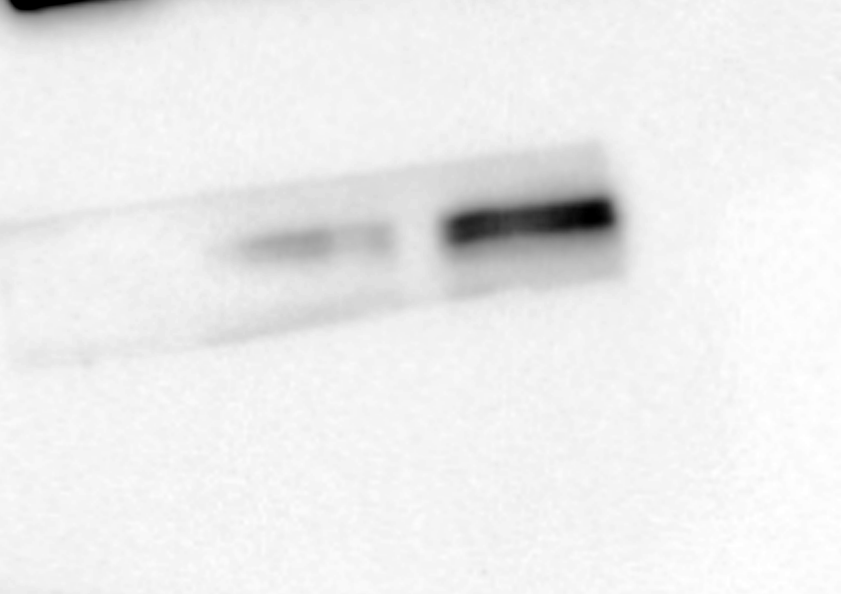
Fig5D. E2F1 (right)


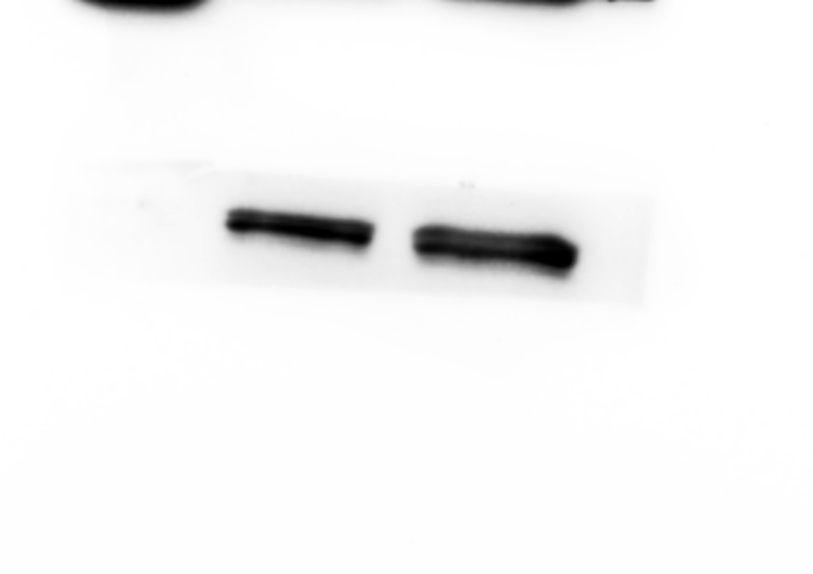
Fig5D. NOP (right)


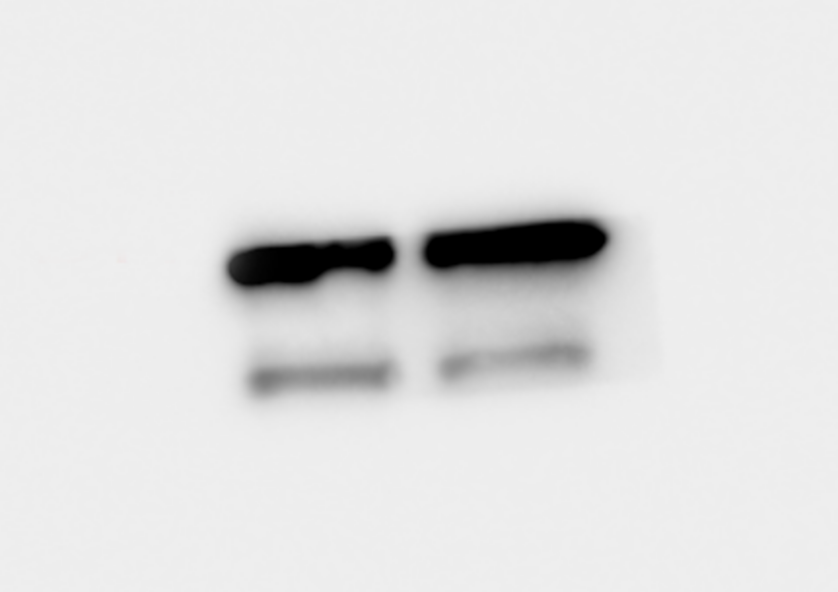
Fig5D. GAPDH (right)


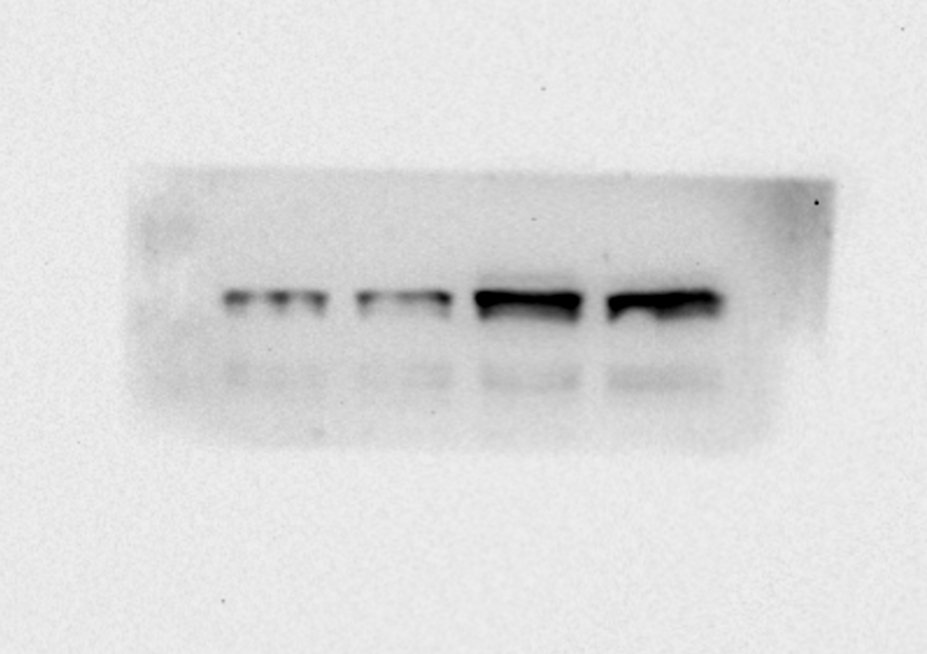
Fig5I. E2F1 (left)


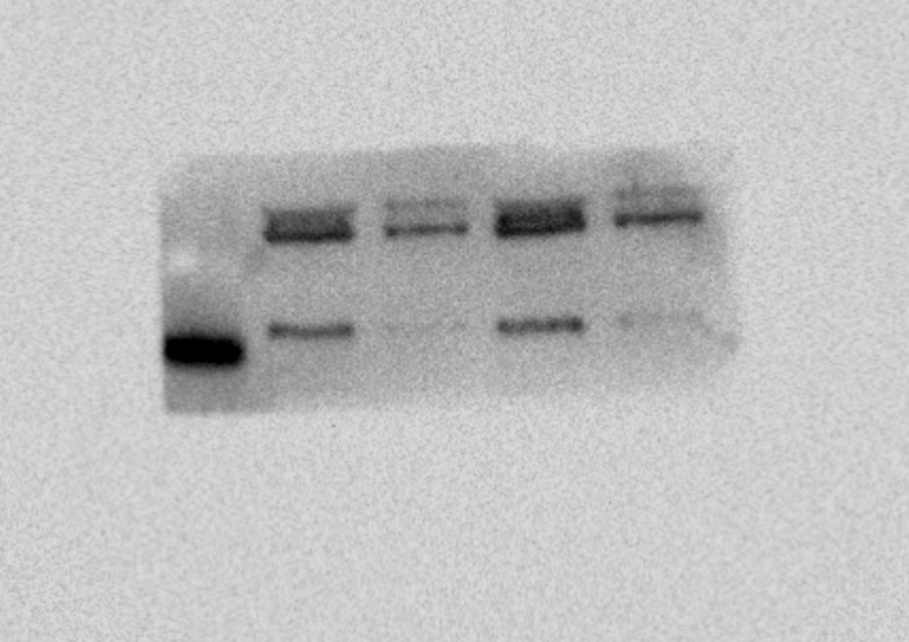
Fig5I. NOP (left)


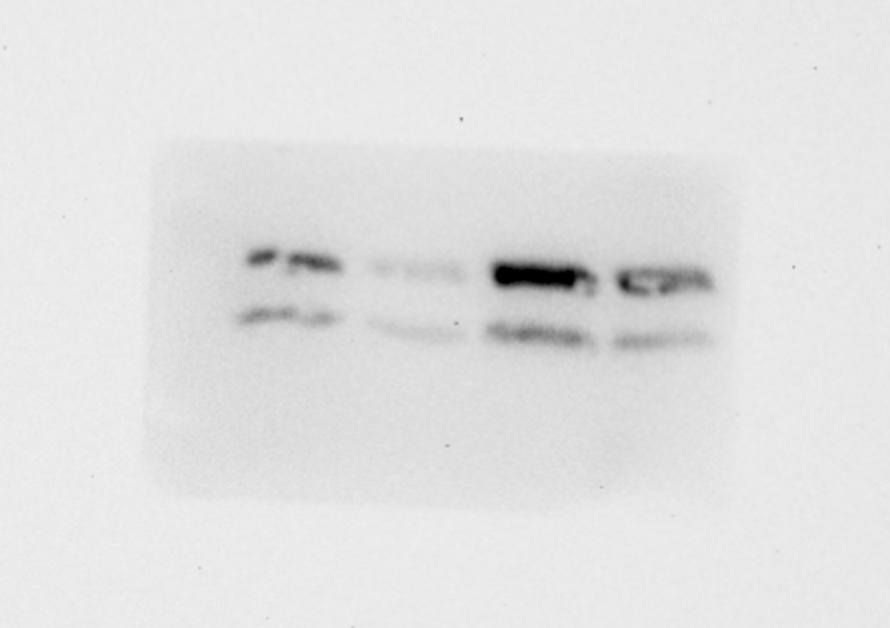
Fig5I. LC3 (left)


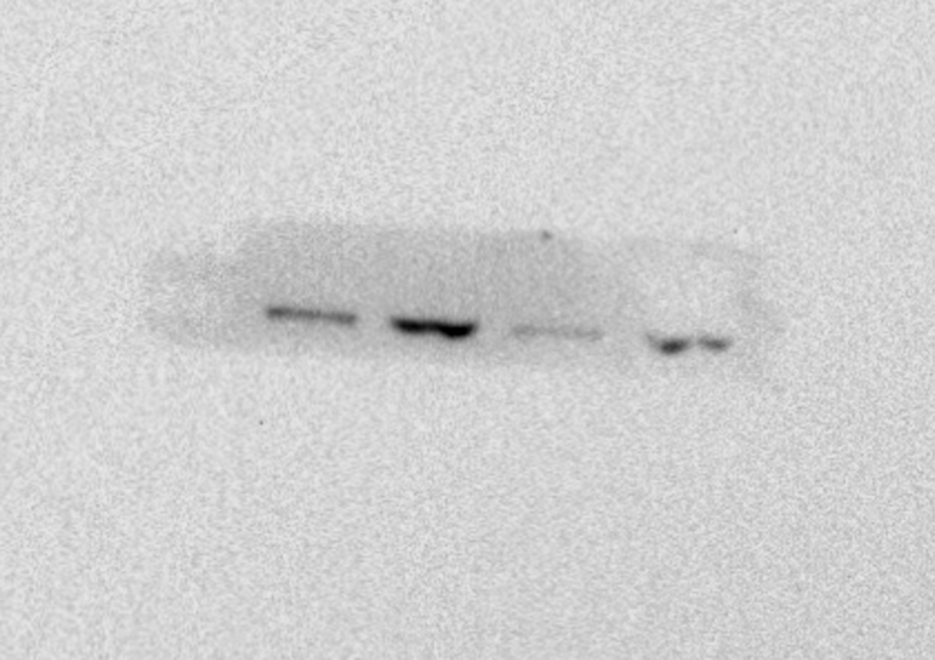
Fig5I. p62 (left)


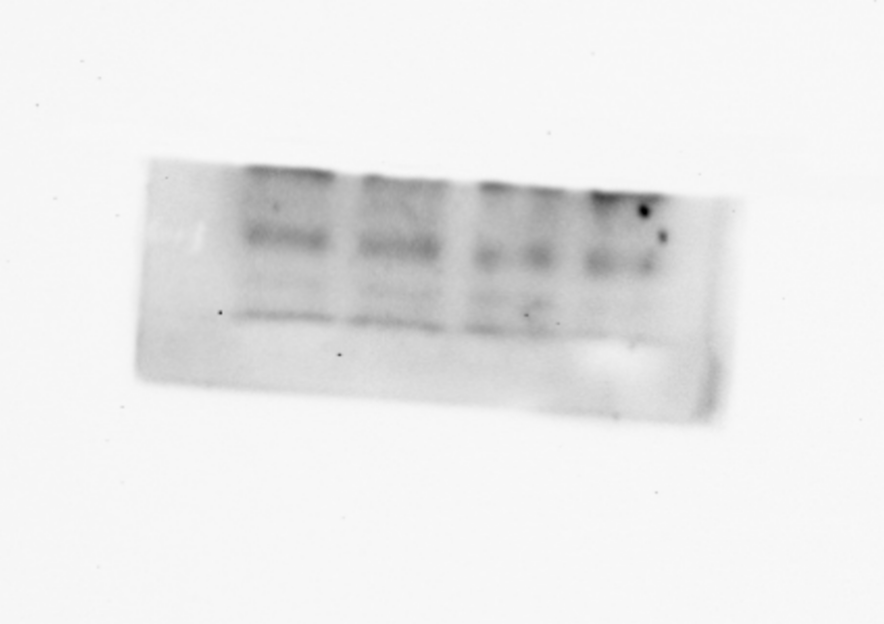
Fig5I caspase3 (left)


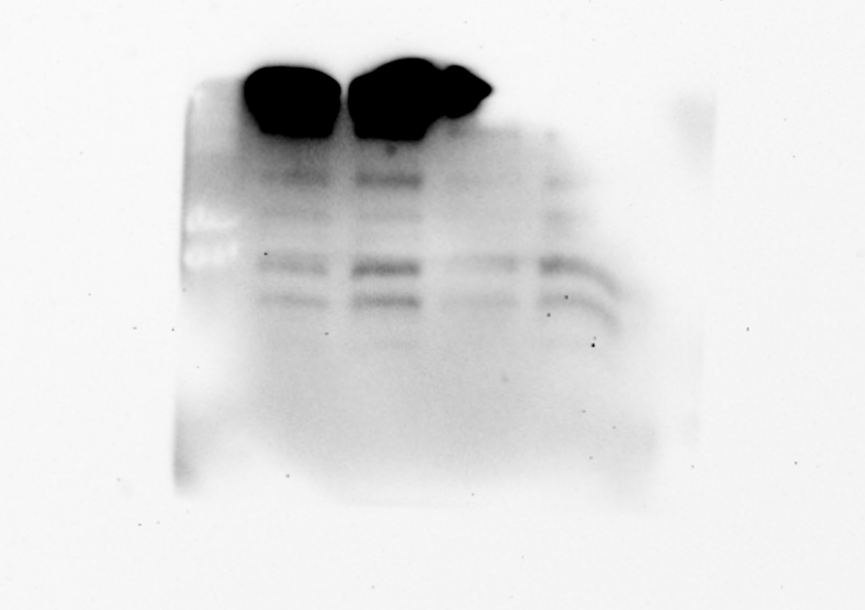
Fig5I. cleaved caspase3 (left)


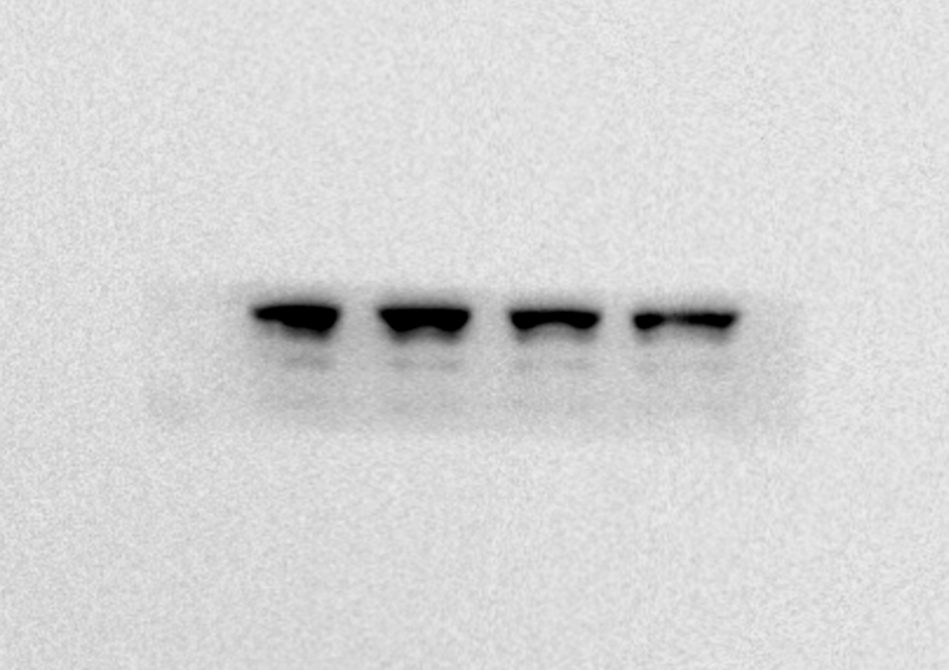
Fig5I p65(left)


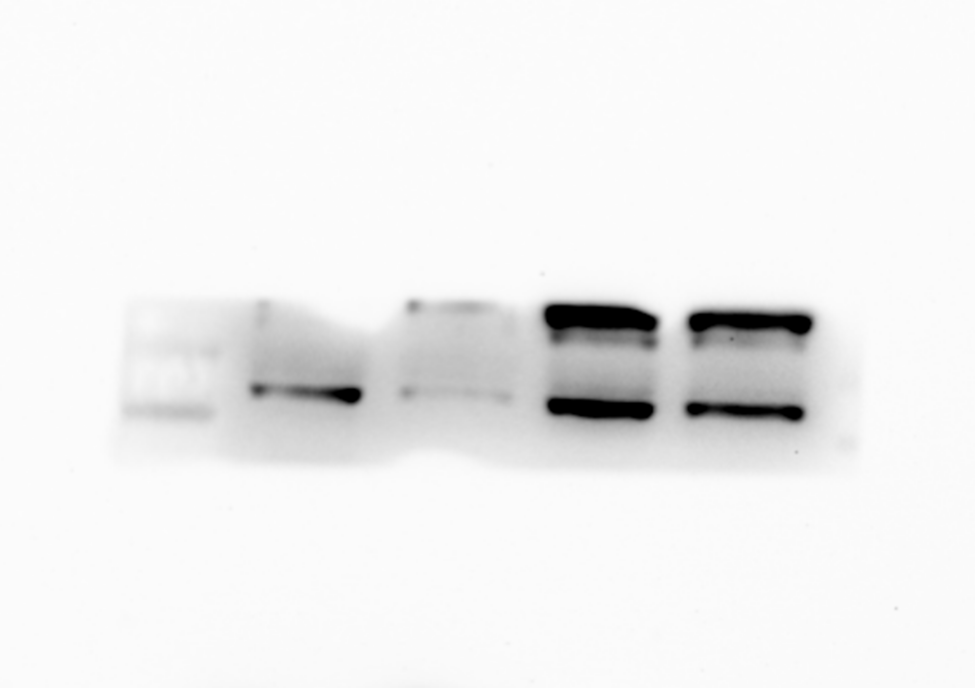
Fig5I p-p65(left)


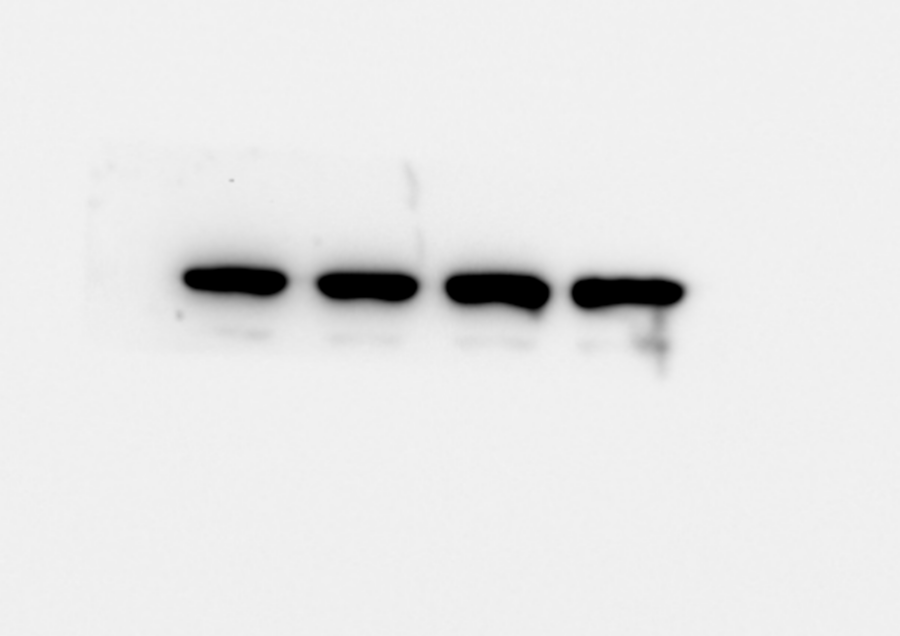
Fig5I GAPDH(left)


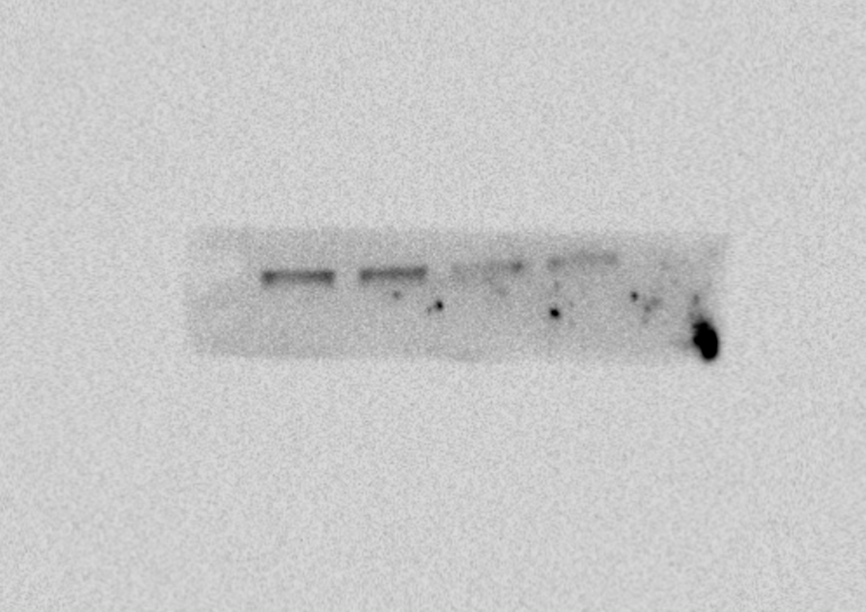
Fig5I E2F1(right)


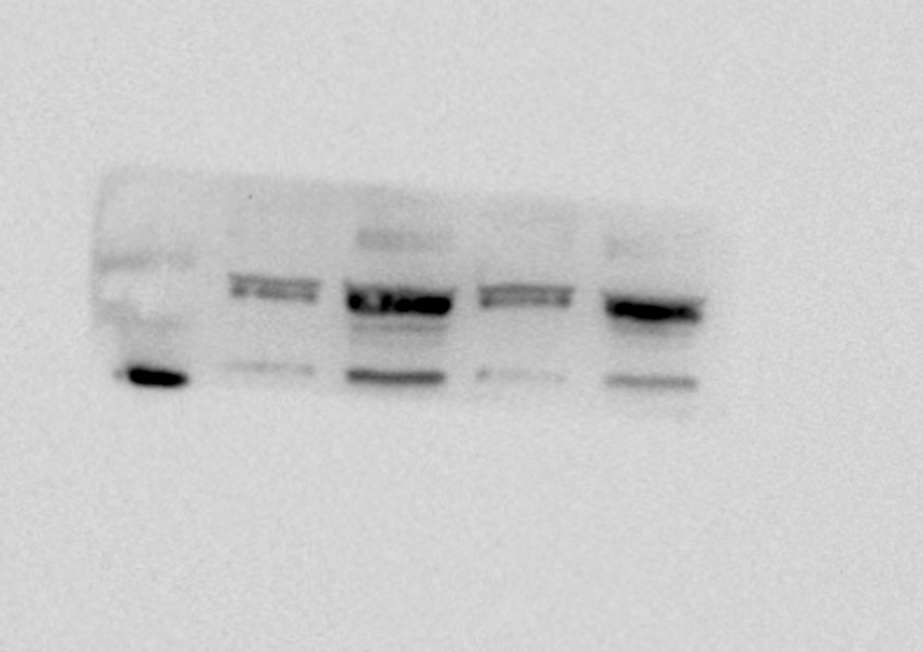
Fig5I NOP(right)


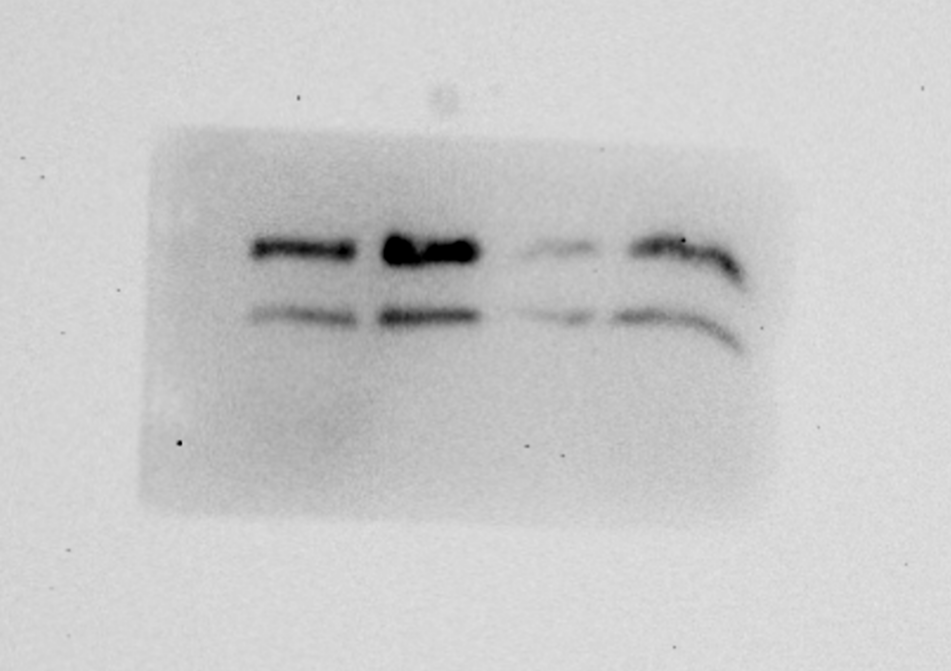
Fig5I LC3(right)


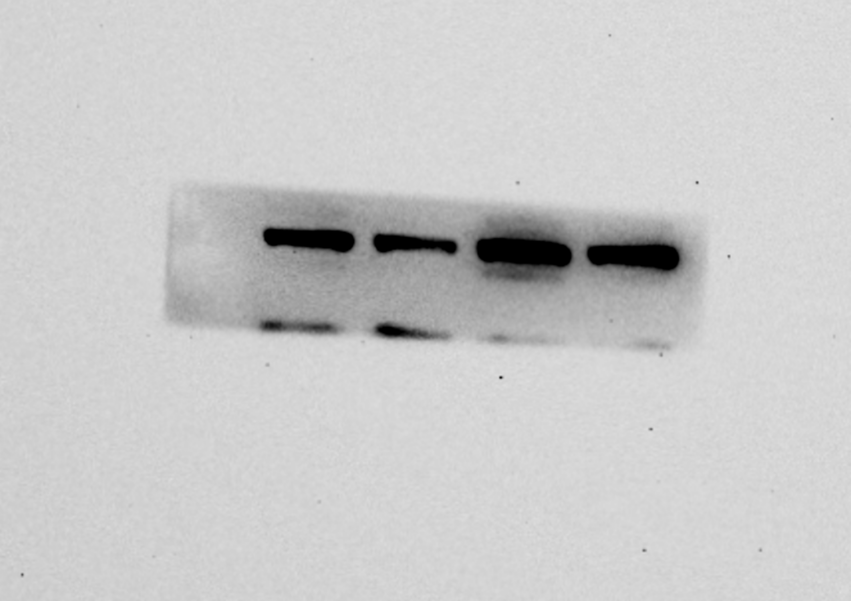
Fig5I p62(right)


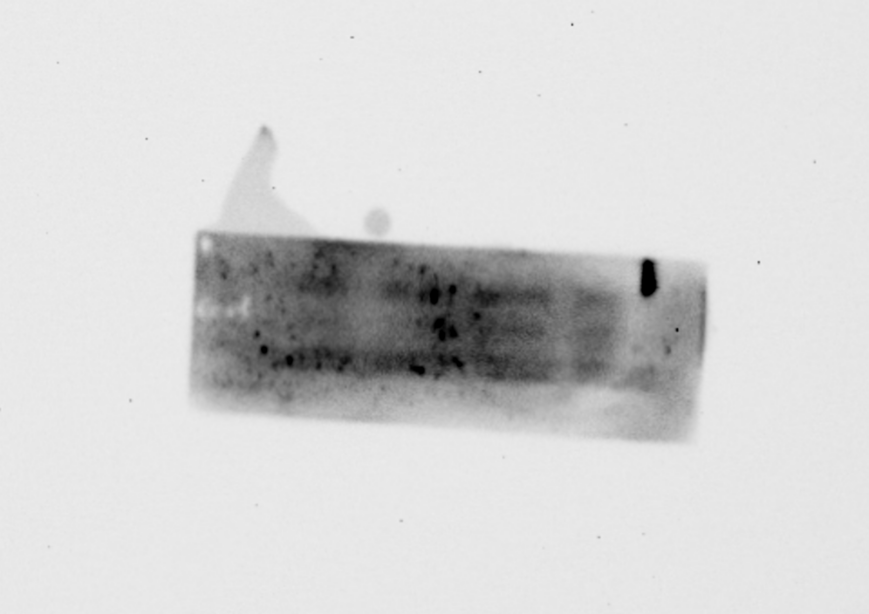
Fig5I Caspase3(right)


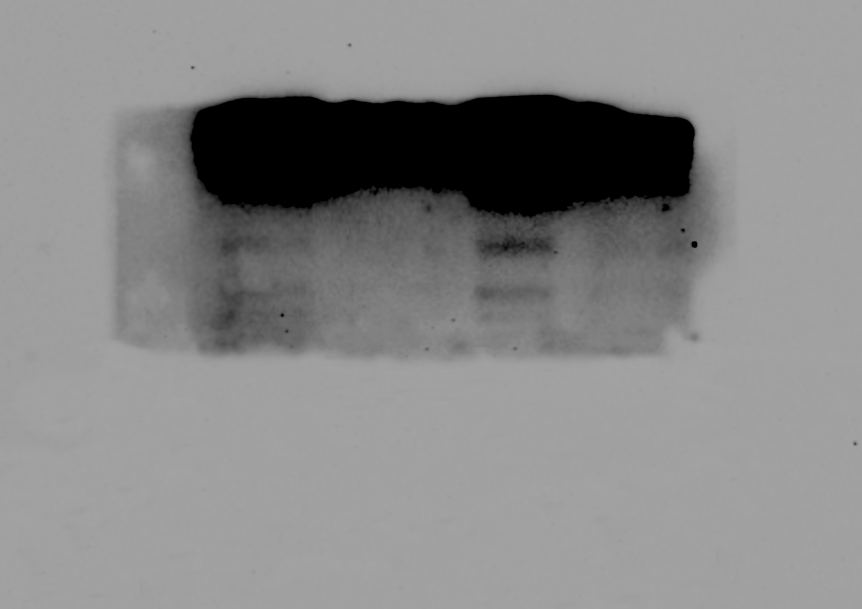
Fig5I cleaved-Caspase3(right)


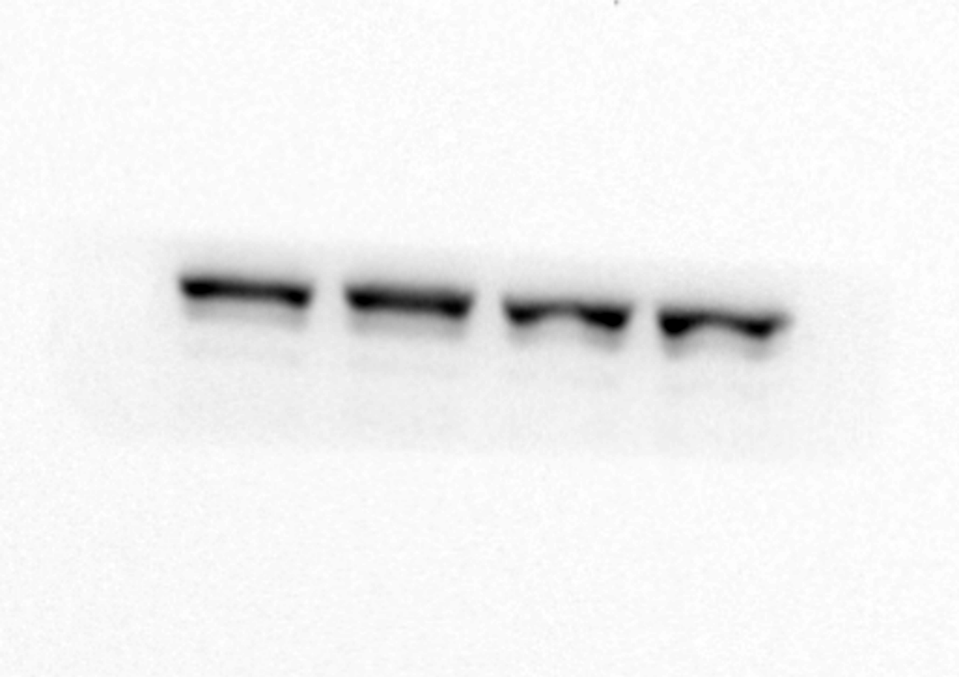
Fig5I p65(right)


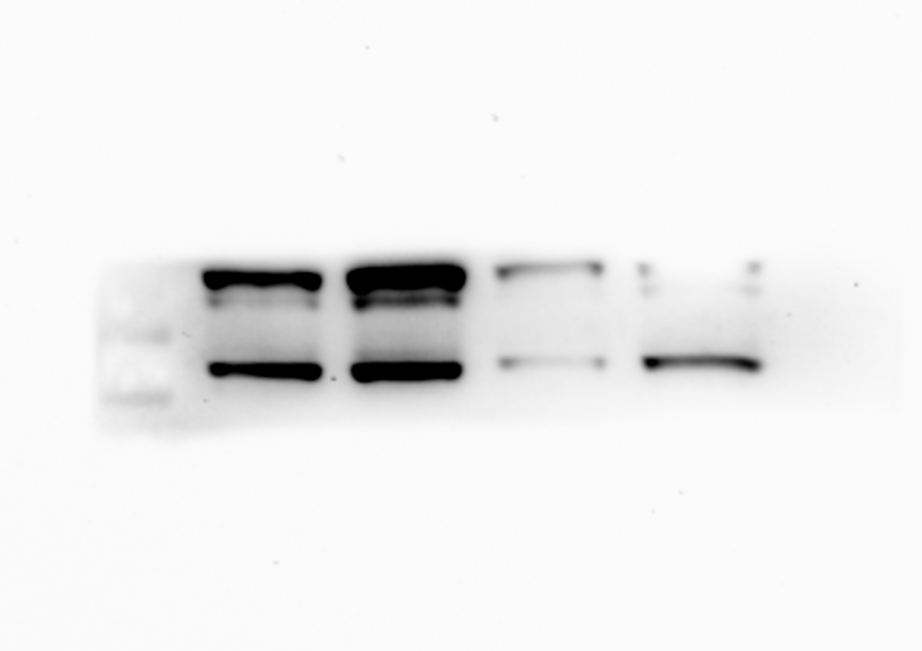
Fig5I p-p65(right)


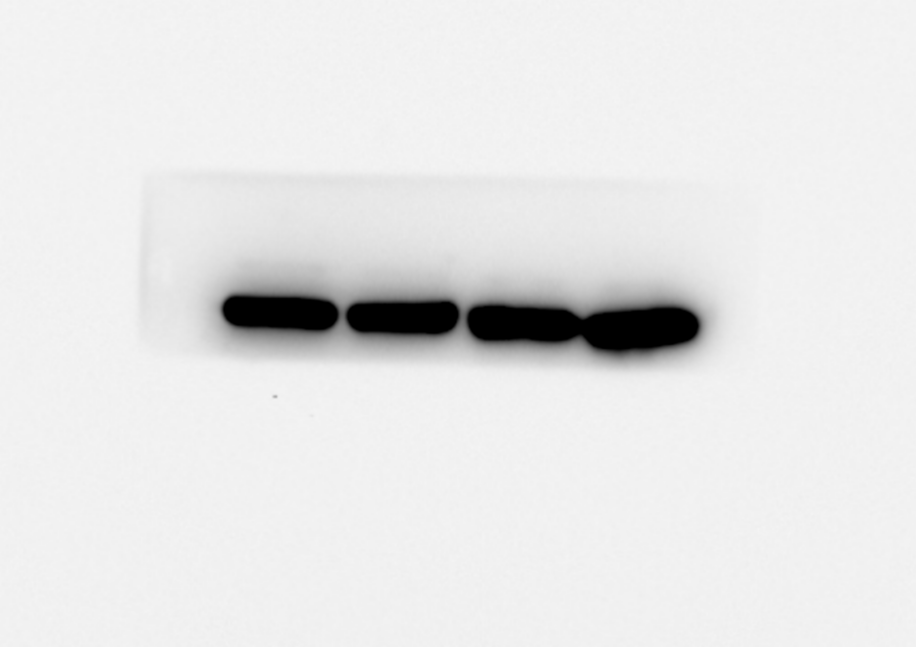
Fig5I GAPDH(right)


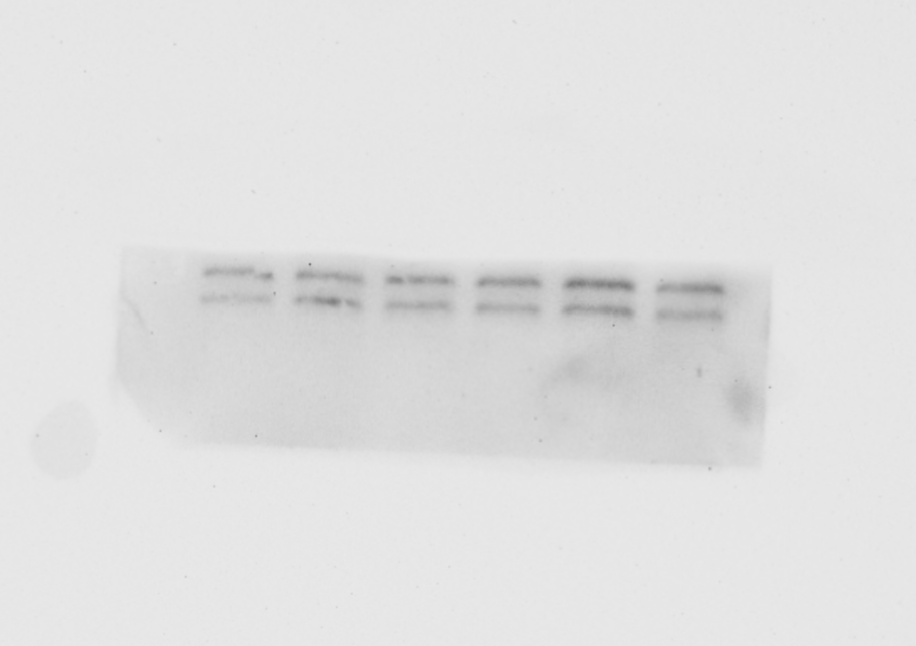
Fig6D Caspase3


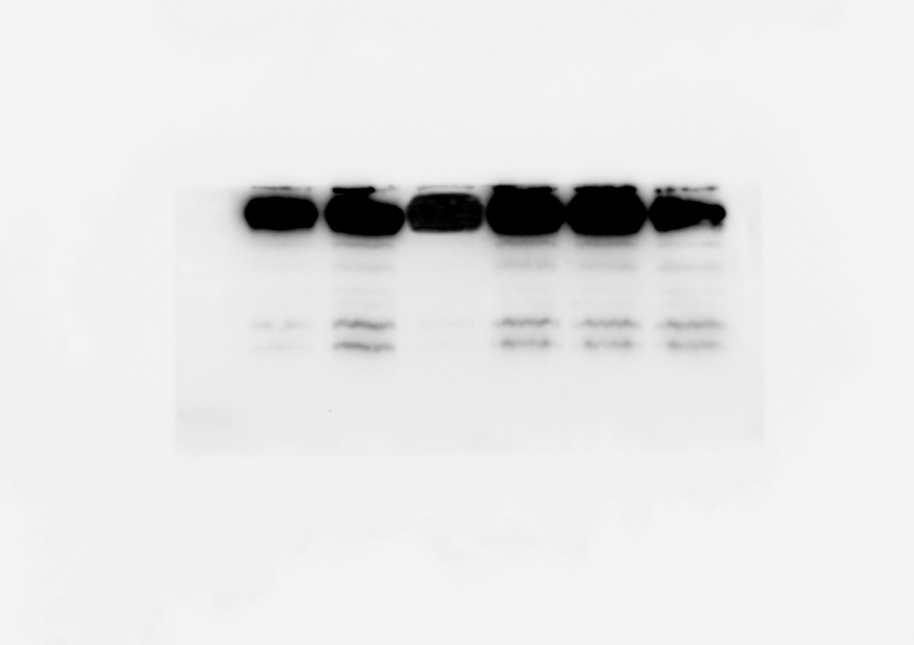
Fig6D cleaved-Caspase3


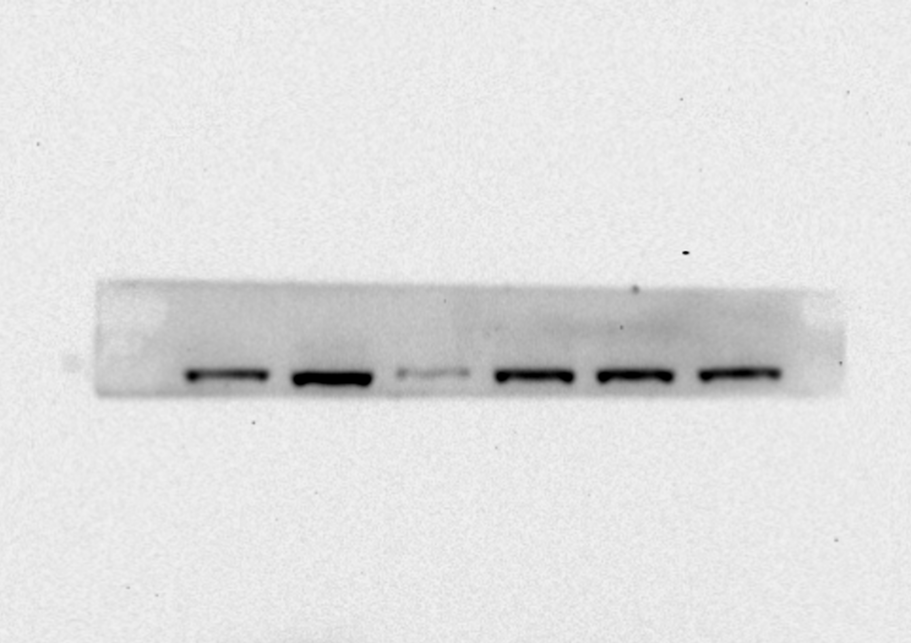
Fig6D p62


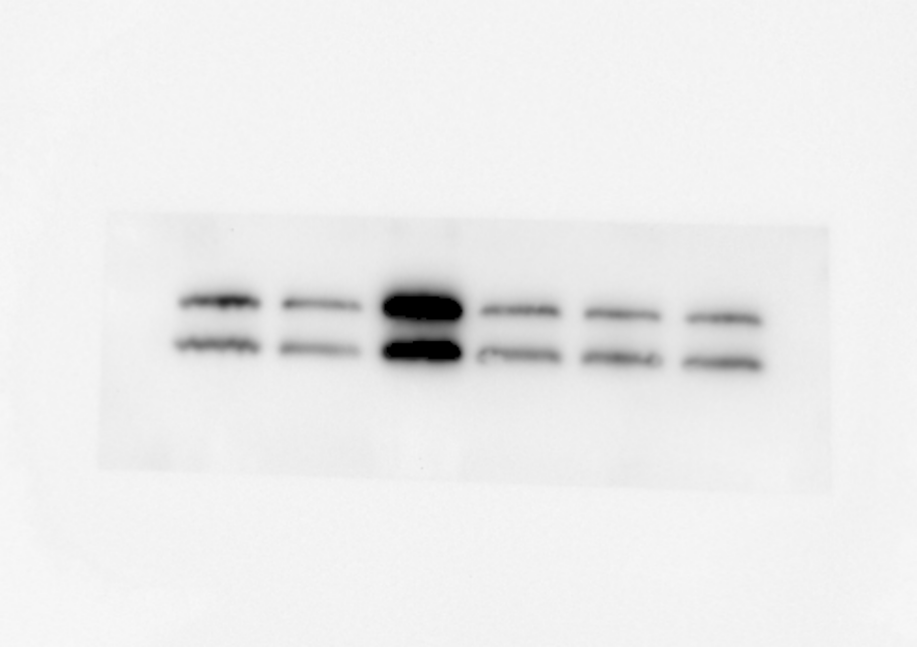
Fig6D LC3


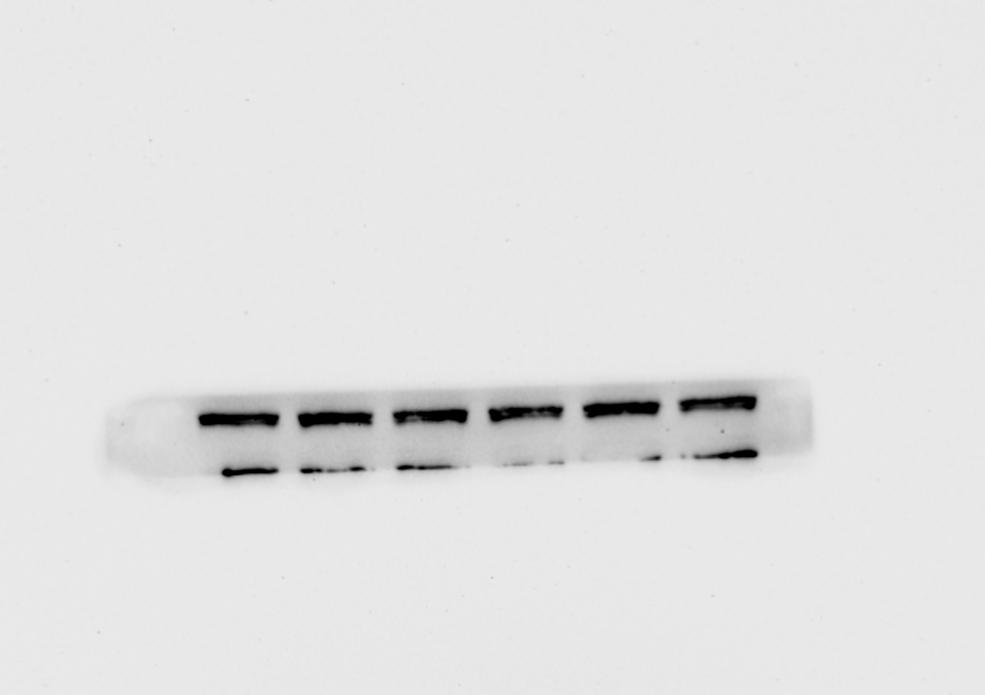
Fig6D p65


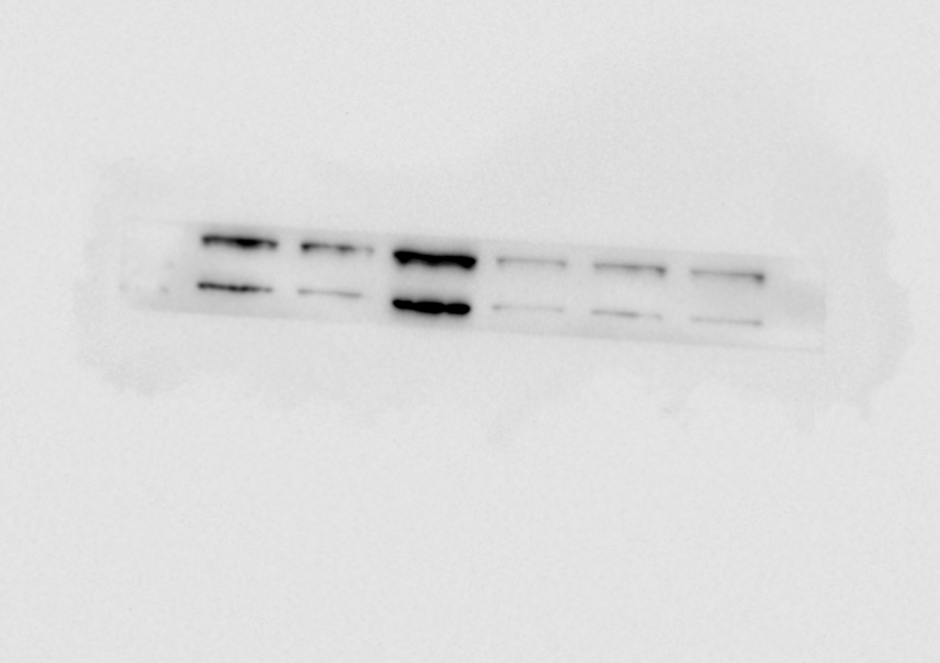
Fig6D p-p65


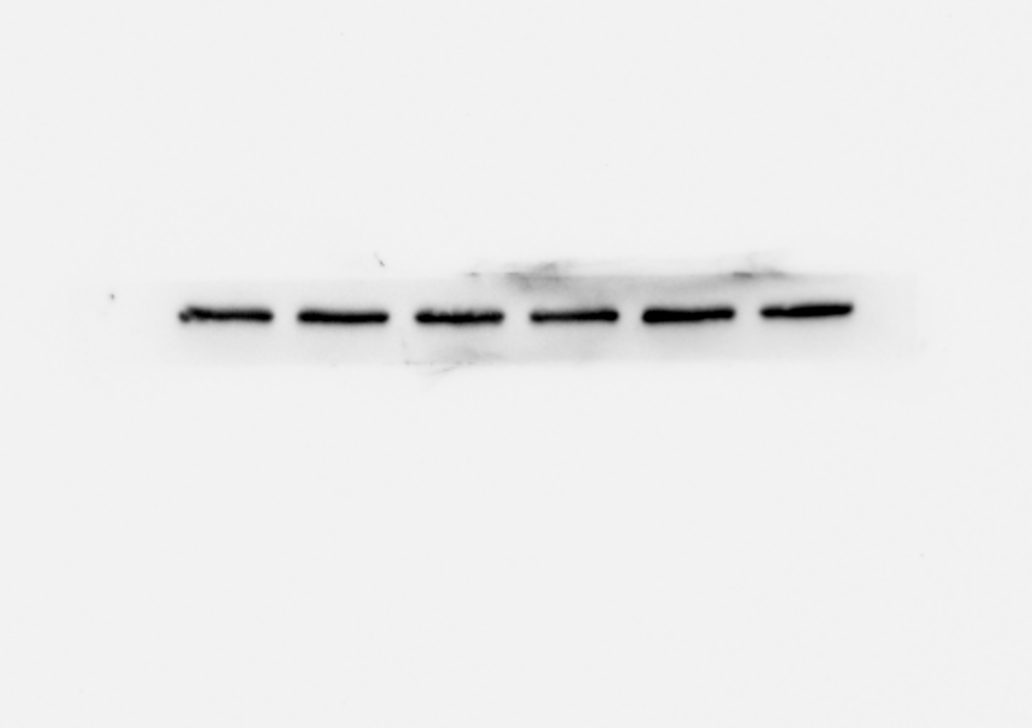
Fig6D GAPDH


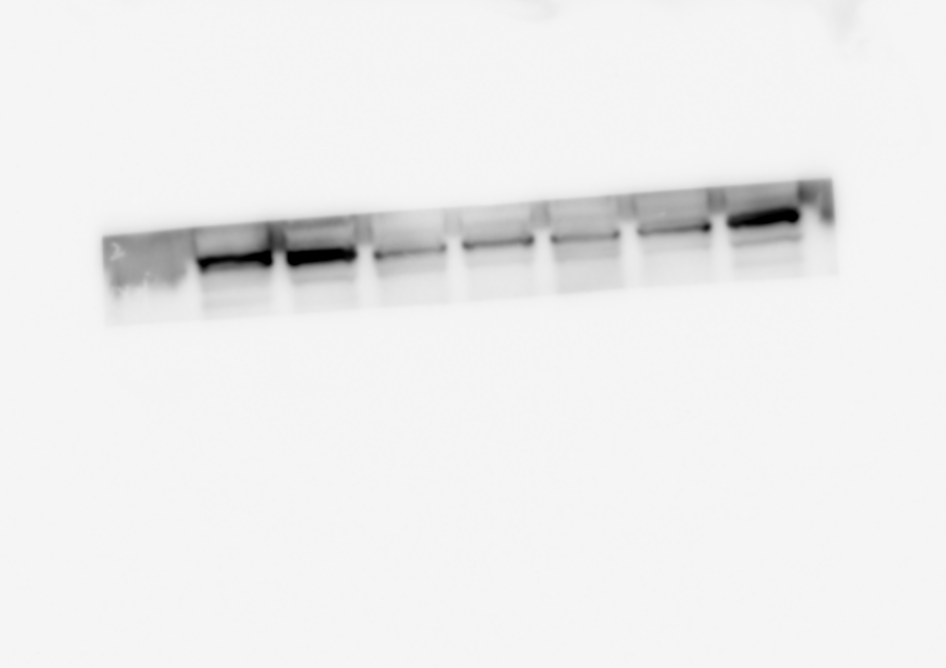
supple Fig1A. NOP


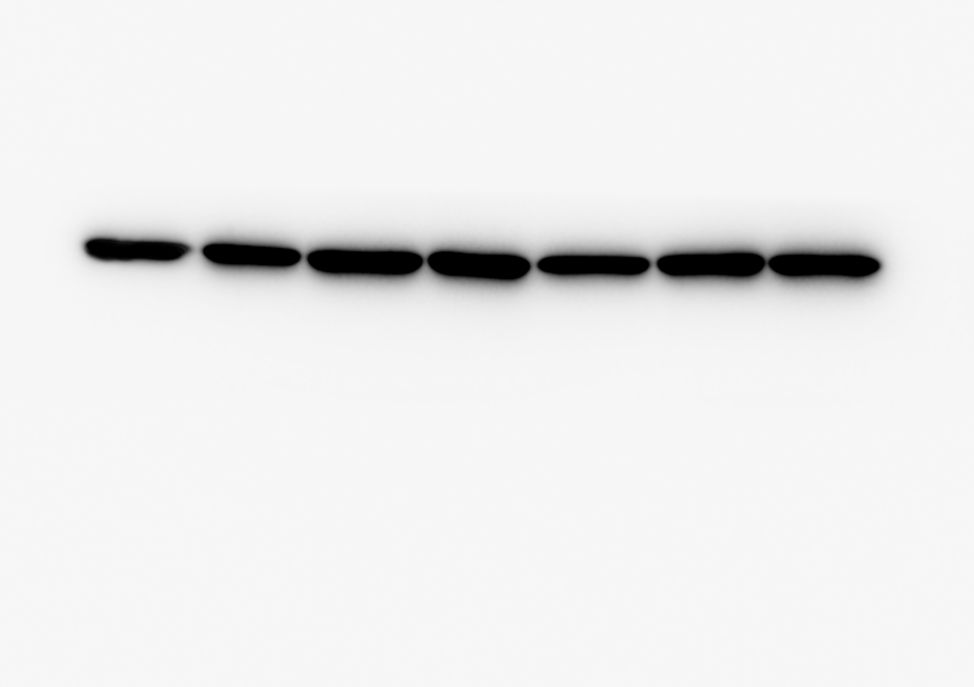
supple Fig1A. GAPDH


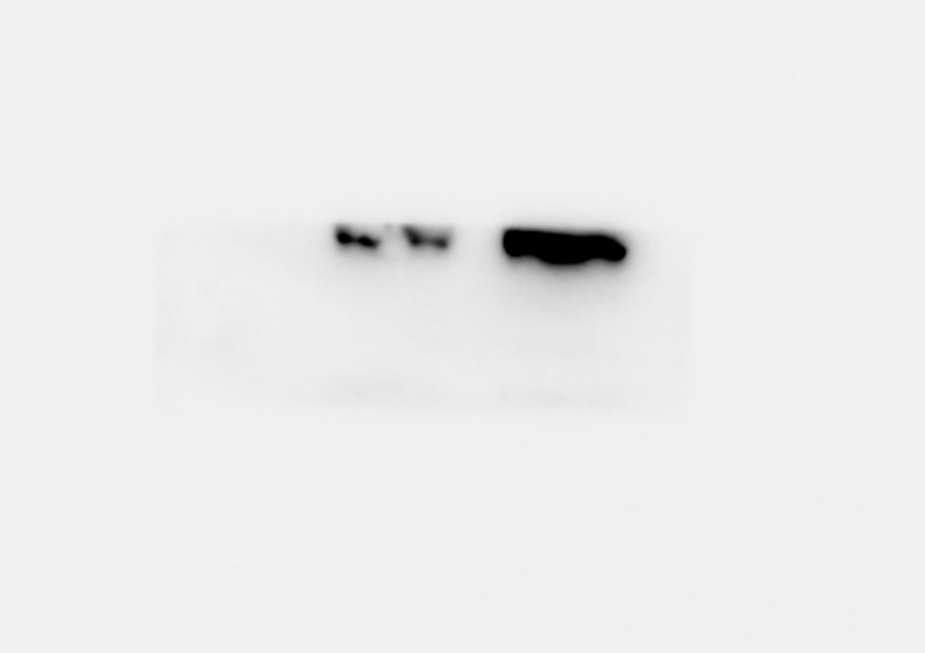
supple Fig1B. NOP (left)


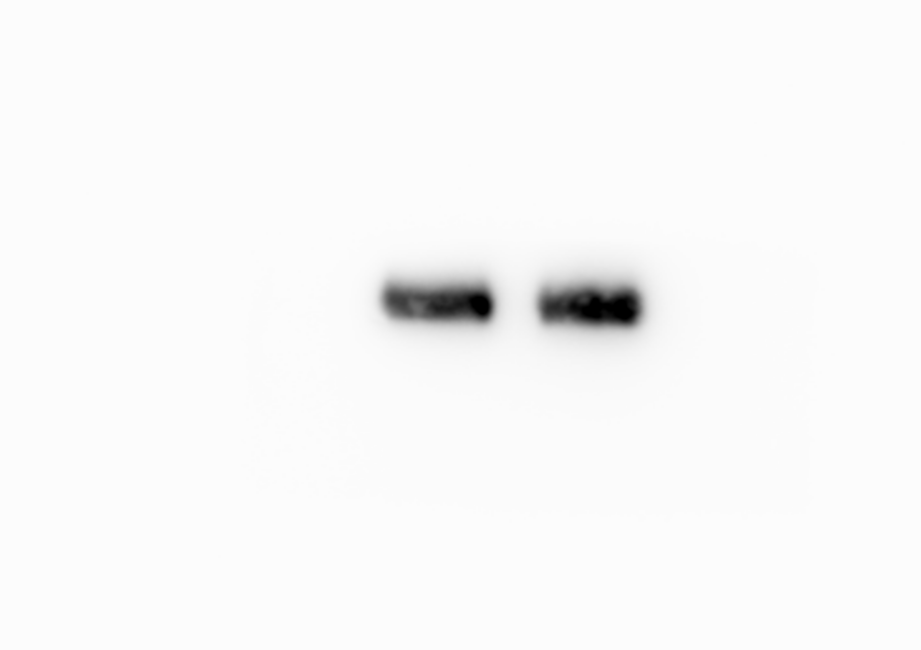
supple Fig1B. GAPDH(left)

supple Fig1B. NOP(right)


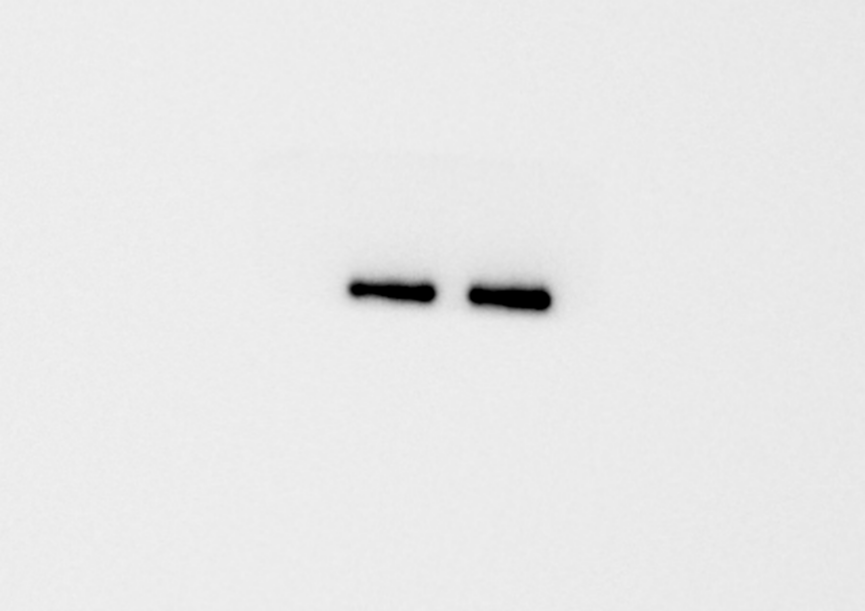
supple Fig1B. GAPDH(right)


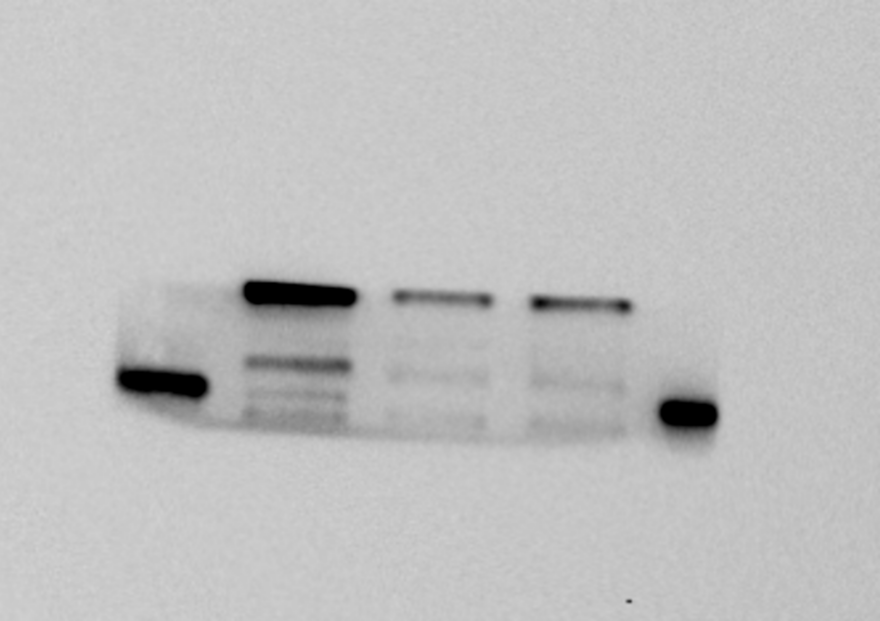
supple Fig1C. NOP(left)


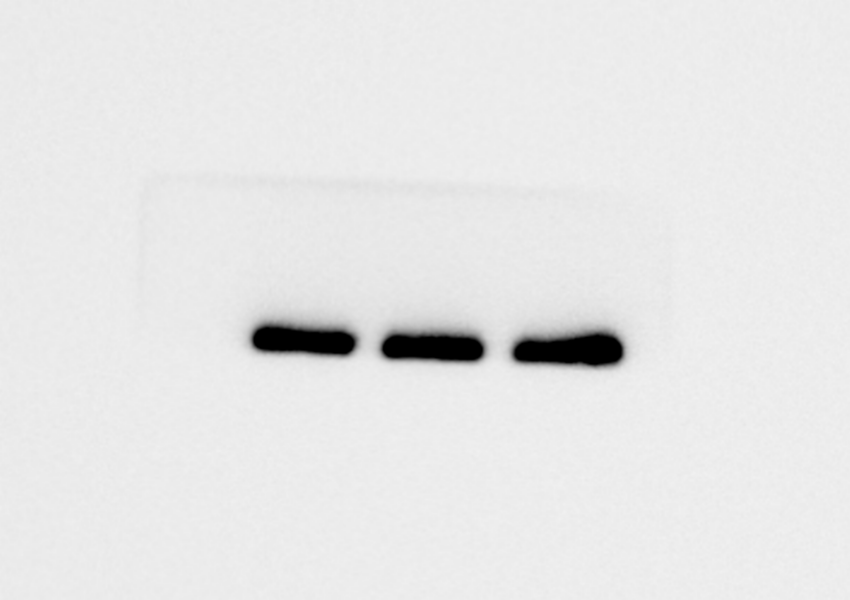
supple Fig1C. GAPDH(left)

supple Fig1C. NOP(right)


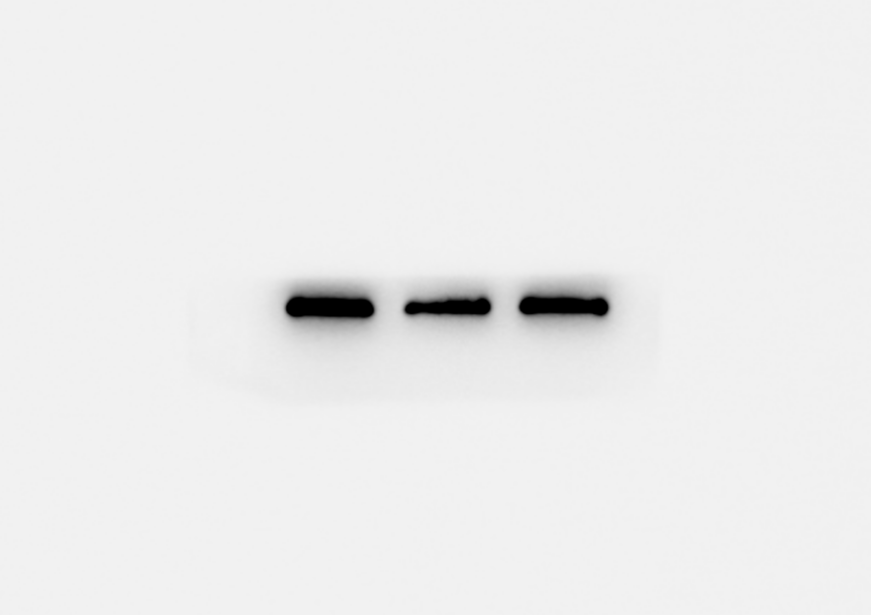
supple Fig1C. GAPDH(right)


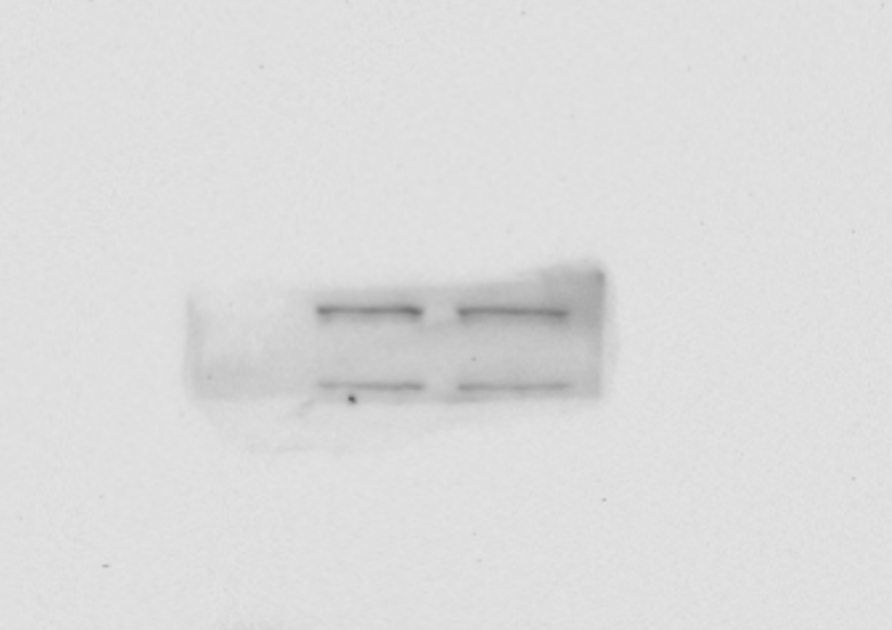
supple Fig2C. p65(left)


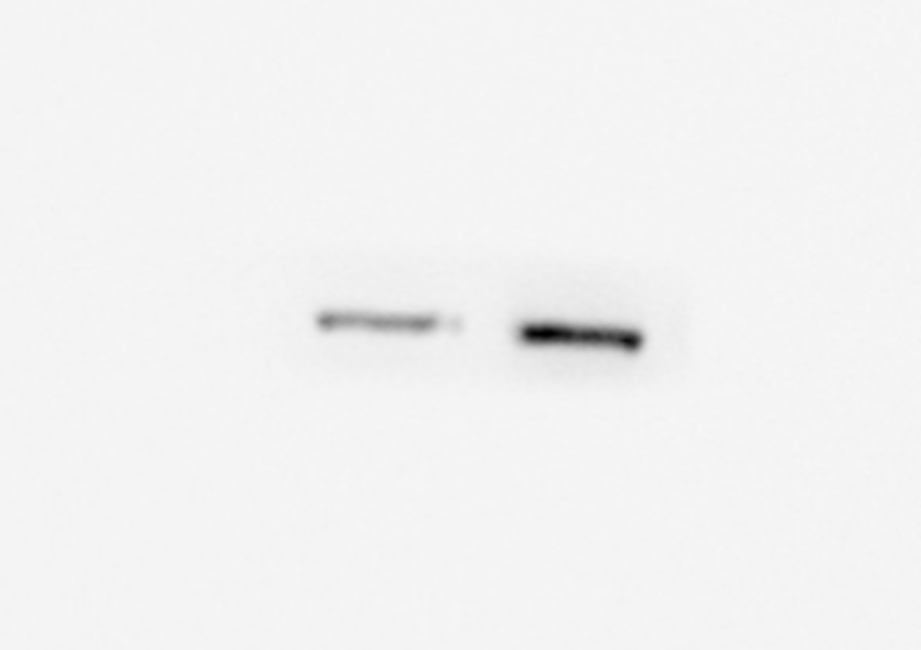
supple Fig2C. p-p65(left)


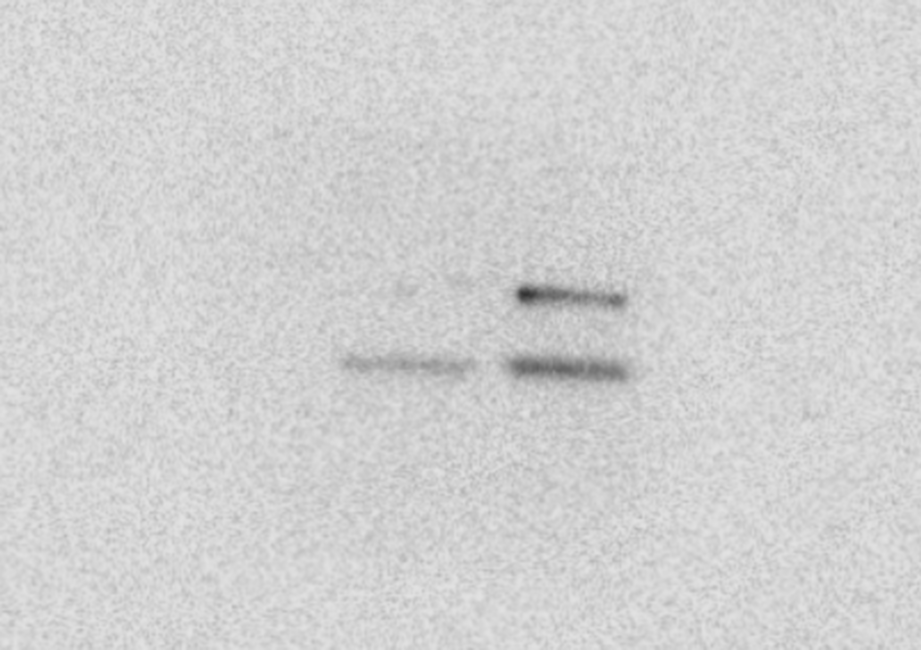
supple Fig2C. LC3(left)


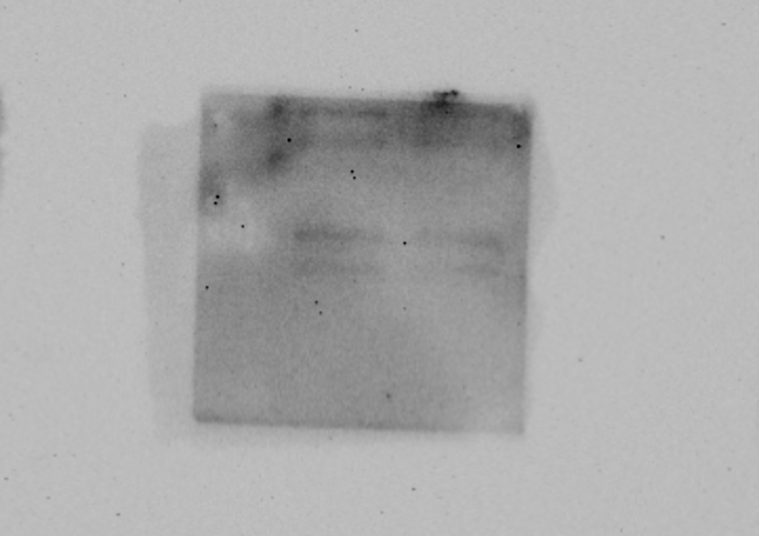
supple Fig2C. cleaved caspase 3(left)

supple Fig2C. GAPDH(left)


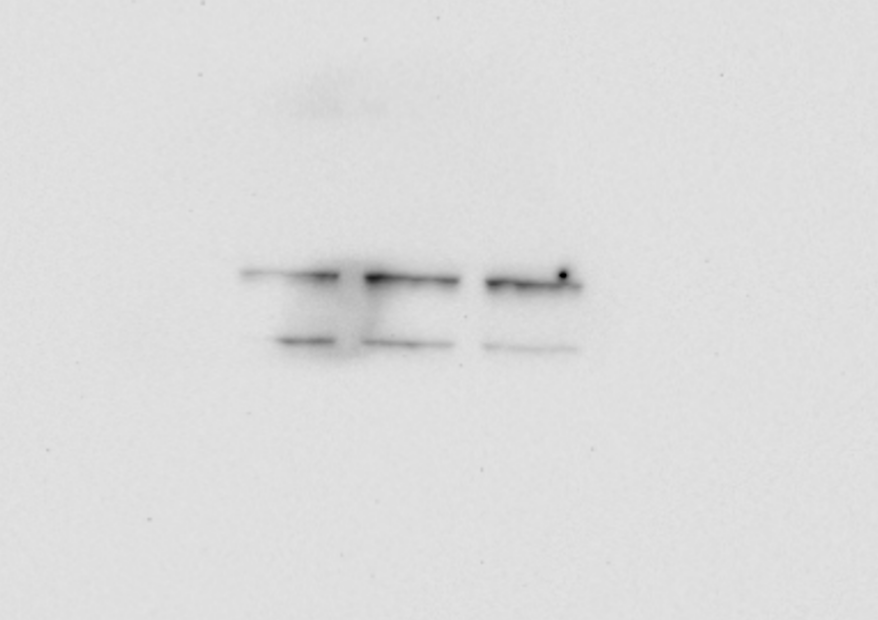
supple Fig2C. p65(right)


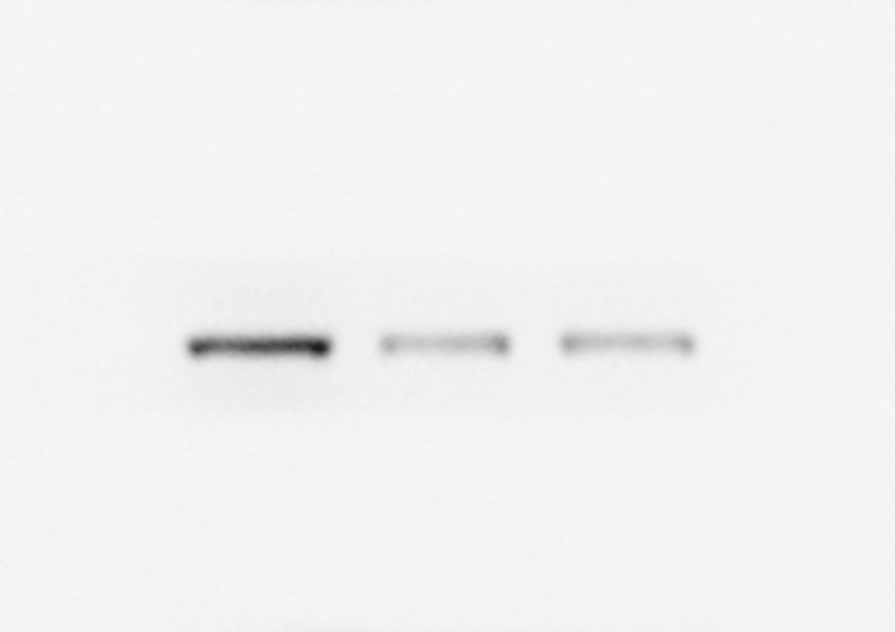
supple Fig2C. p-p65(right)


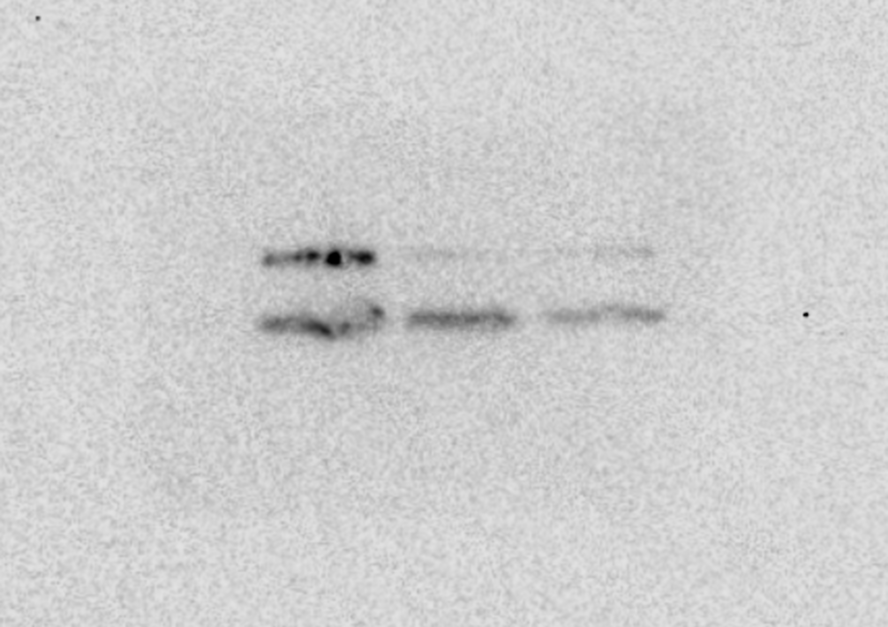
supple Fig2C. LC3(right)


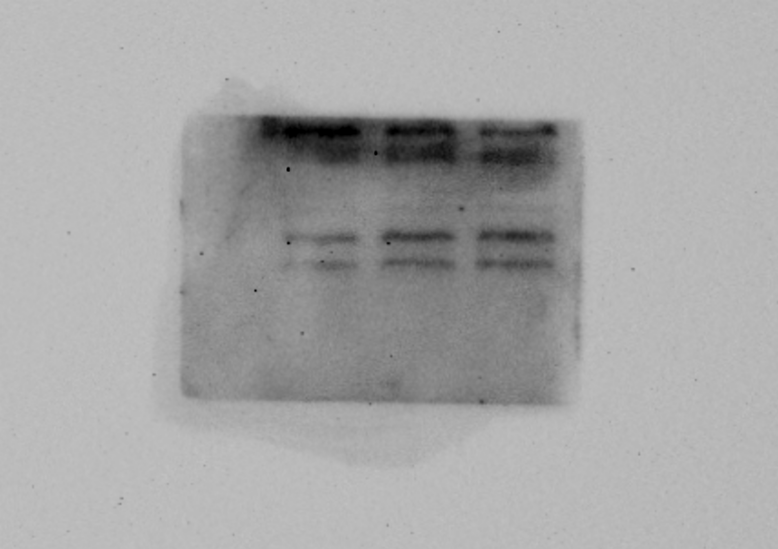
supple Fig2C. cleaved caspase3(right)

supple Fig2C. GAPDH(right)
