# Supplementary material for: The nociceptin receptor promotes autophagy through NF-kB signaling and is transcriptionally regulated by E2F1 in HCC
Source: Cell Death Discov. 2022 Apr 5;8:165. doi: 10.1038/s41420-022-00978-7 (PMC8983730; doi:10.1038/s41420-022-00978-7)
Supplement: Supplementary file 2 — final supplementary material [file 41420_2022_978_MOESM2_ESM.docx]

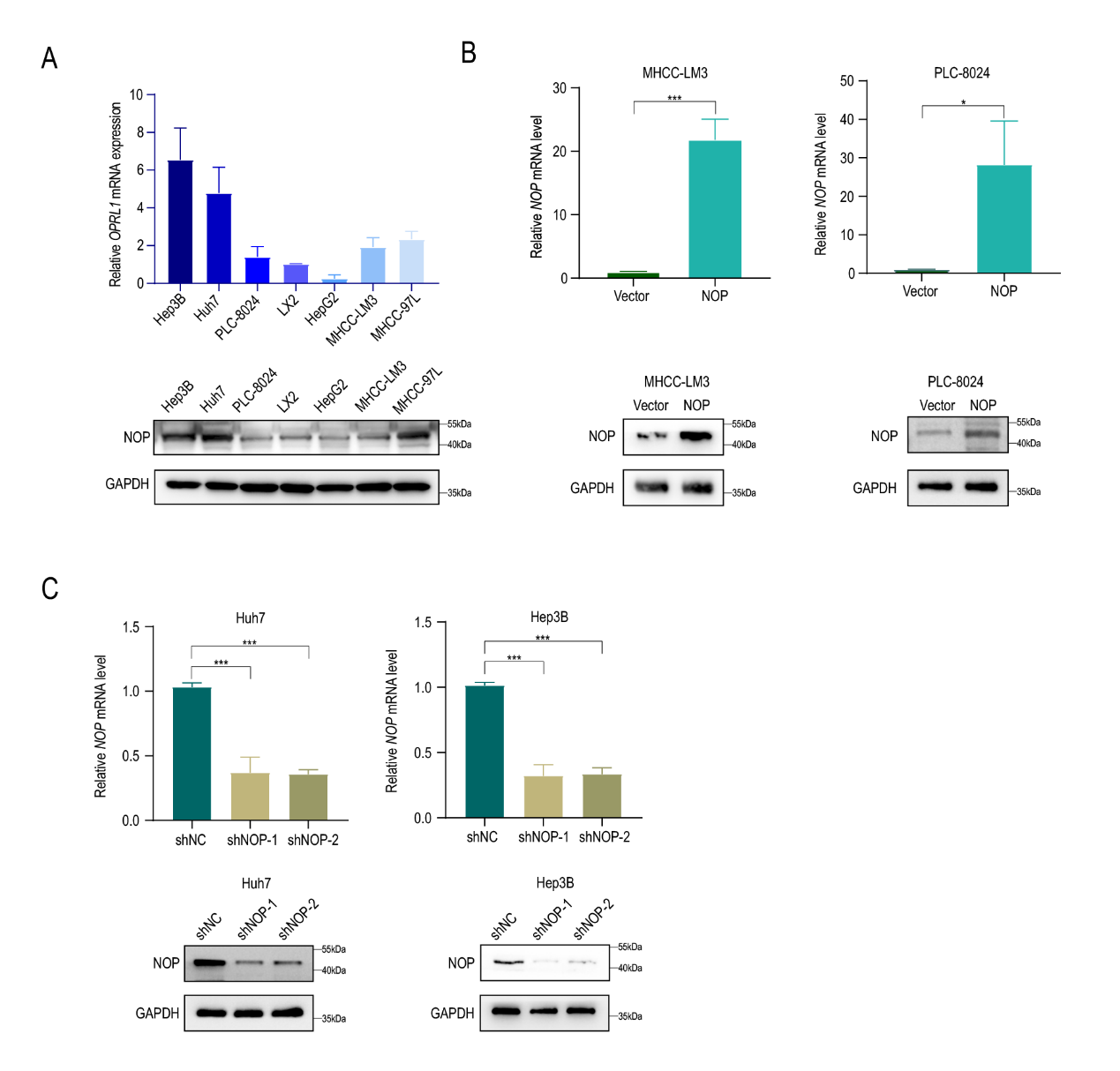


**Suppl. Fig. 1. Stable transfection of HCC cell lines with the NOP overexpression plasmid and shNOP plasmid.**

A. NOP mRNA levels and protein levels in HCC cell lines were tested by qRT–PCR and western blot analyses.

B. Western blot analysis confirmed the overexpression of NOP in MHCC-LM3 cells and PLC-8024 cells by stable transfection.

C. Western blot analysis confirmed the knockdown of NOP expression in Huh7 cells and Hep3B cells by stable transfection.


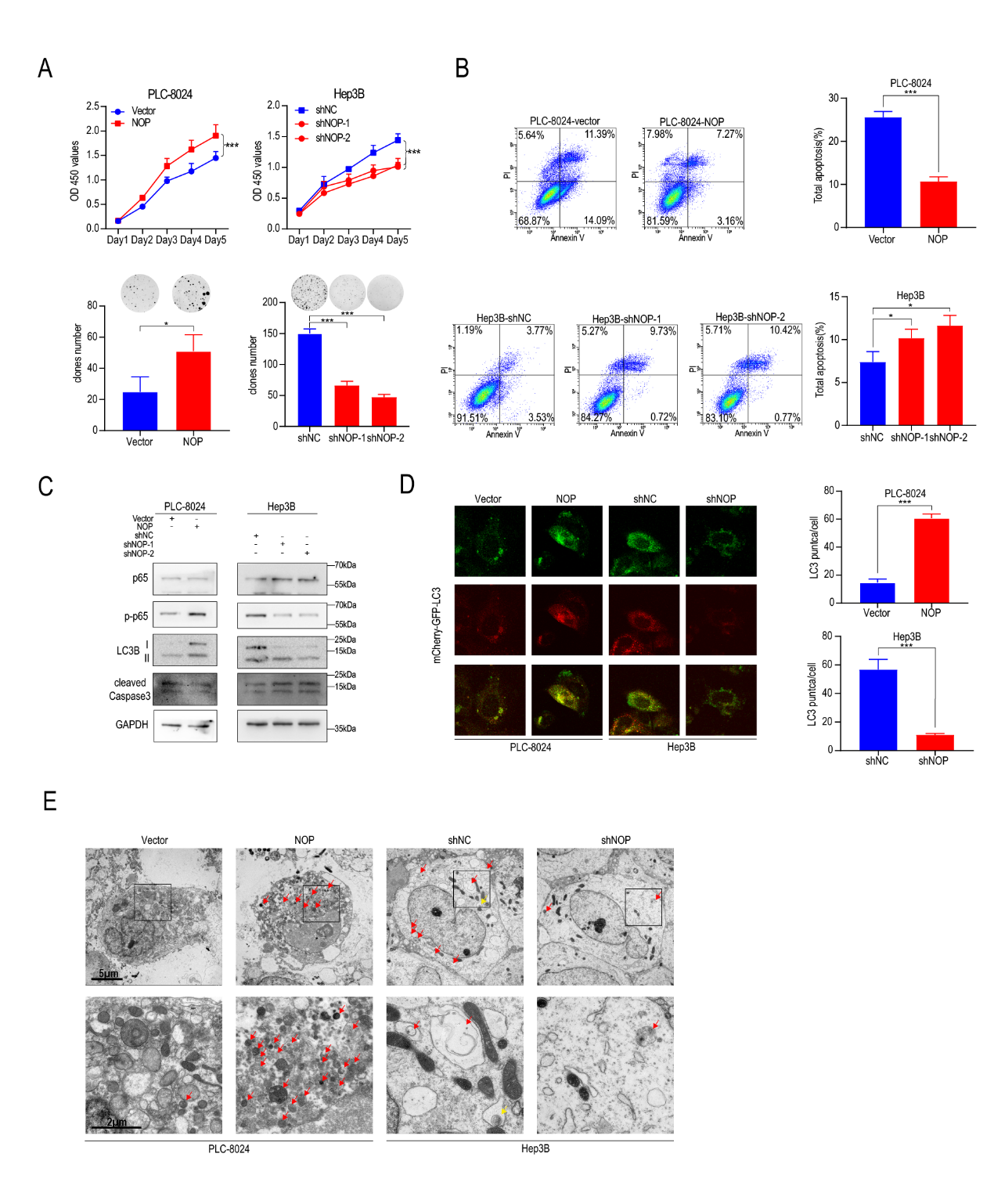


**Suppl. Fig. 2. NOP influences the proliferation, apoptosis and autophagy of HCC cells**

A. CCK-8 assay and colony formation assay showing the proliferation of PLC-8024-vector, PLC-8024-NOP, Hep3B-shNC, Hep3B-shNOP-1 and Hep3B-shNOP-2 cells.

B. Apoptosis rates in PLC-8024-vector, PLC-8024-NOP HCC cells, Hep3B-shNC, Hep3B-shNOP-1 and Hep3B-shNOP-2 HCC cells.

C. IB analysis of the indicated proteins associated with apoptosis and autophagy in PLC-8024 and Hep3B cells.

D. IF staining showing the GFP-mCherry-LC3B fusion protein in PLC-8024-vector, PLC-8024-NOP, Hep3B-shNC, and Hep3B-shNOP cells (GFP: green, mCherry: red; merged: yellow). Scale bars=20 μm.

E. Electron microscopy showing the autolysosome (red arrow) and autophagosome (yellow arrow) numbers in PLC-8024-vector, PLC-8024-NOP, Hep3B-shNC, and Hep3B-shNOP cells. Scale bars=2 μm.


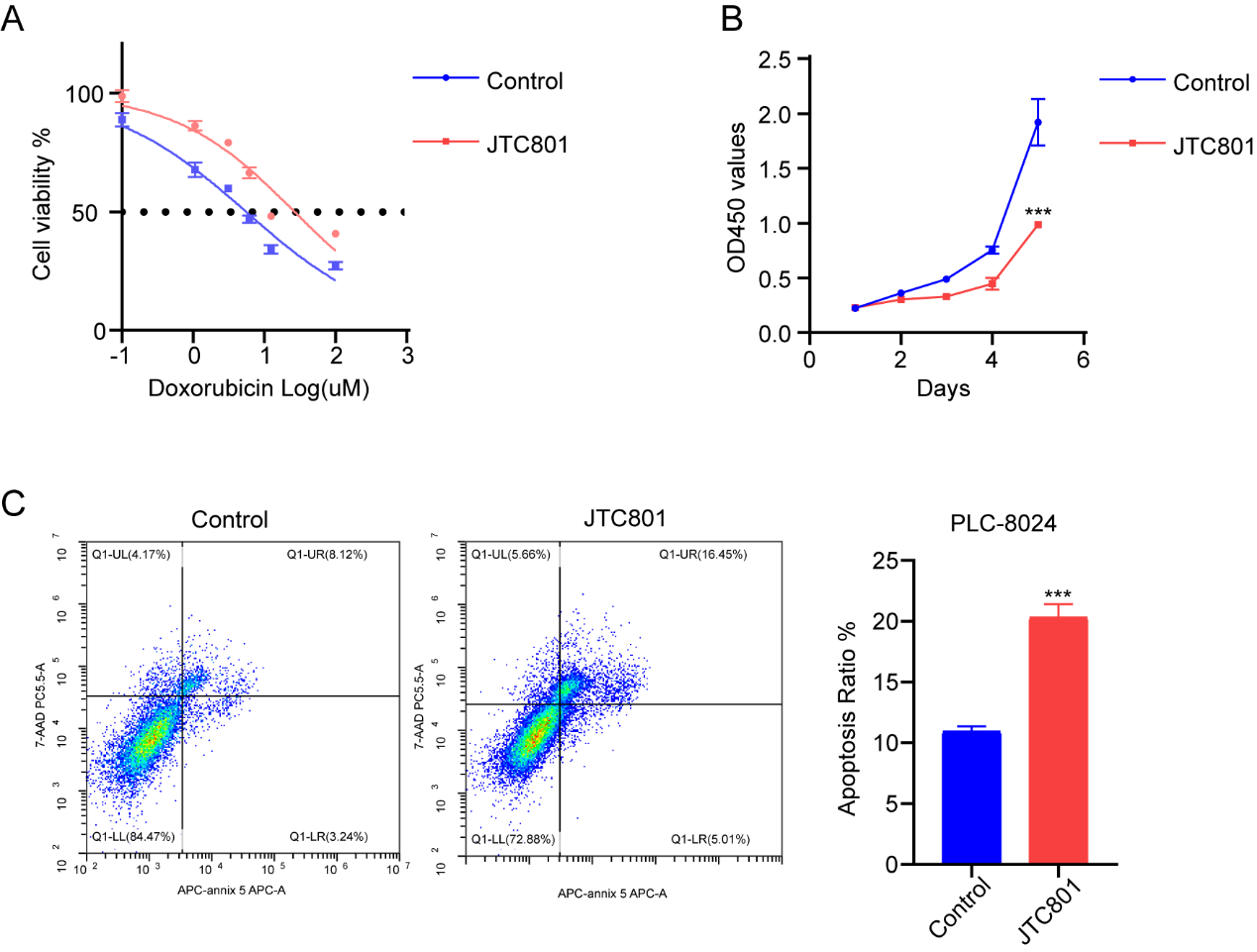


**Supple. Fig. 3 JTC-801 played synergistic effects on doxorubicin**

A. IC50 of doxorubicin in HCC cells treated with JTC801.

B. CCK8 assay accessed effects of JTC801 on cell proliferation of PLC-8024 under doxorubicin treatment.

C. Effects of JTC801 on cell apoptosis of PLC-8024 under doxorubicin treatment.
