# Supplementary figures and images for: The nociceptin receptor promotes autophagy through NF-kB signaling and is transcriptionally regulated by E2F1 in HCC
Source: Cell Death Discov. 2022 Apr 5;8:165. doi: 10.1038/s41420-022-00978-7 (PMC8983730; doi:10.1038/s41420-022-00978-7)

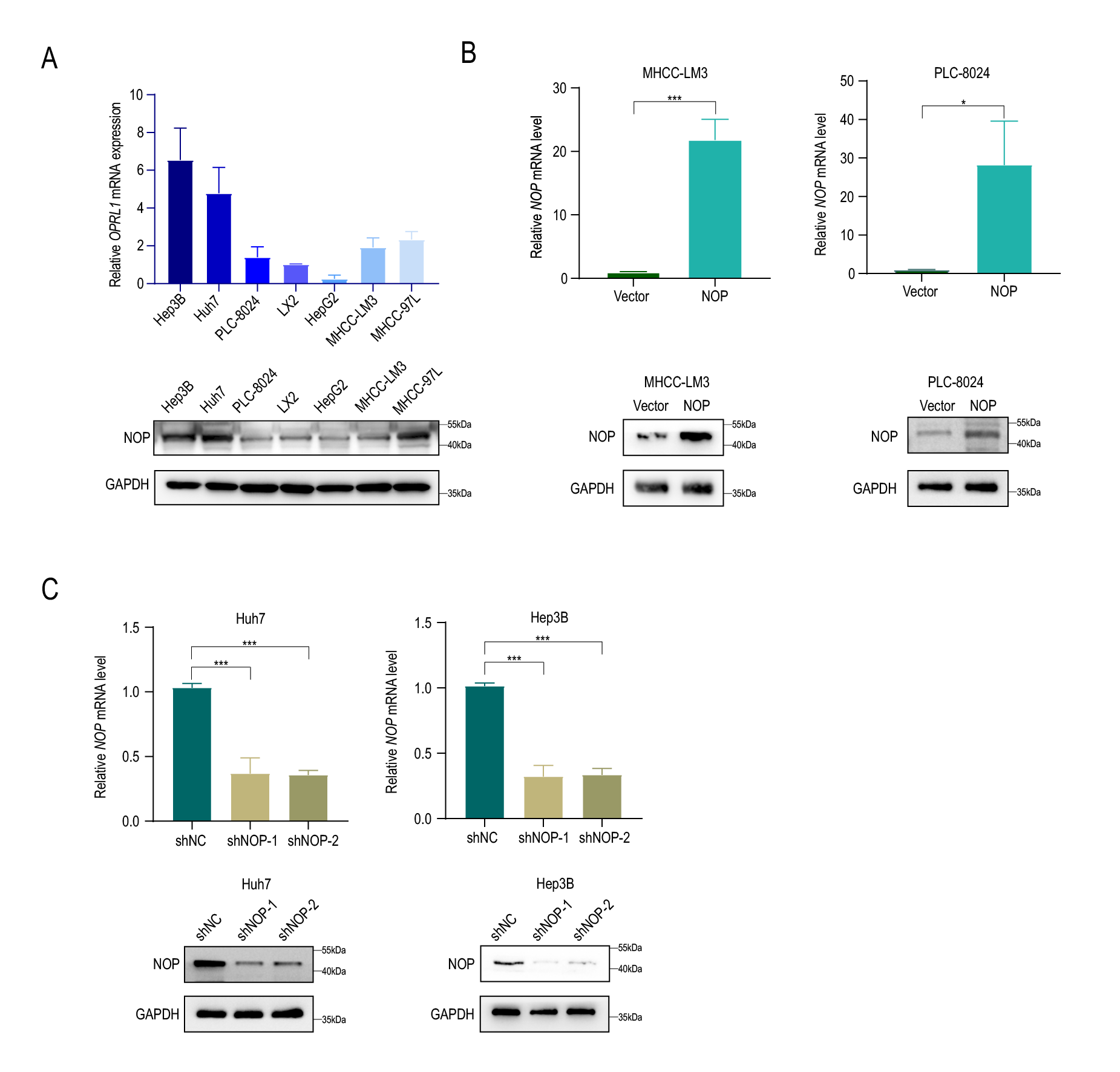

Supplement: Supplementary file 3 — SuppleFig1 [file 41420_2022_978_MOESM3_ESM.tif]

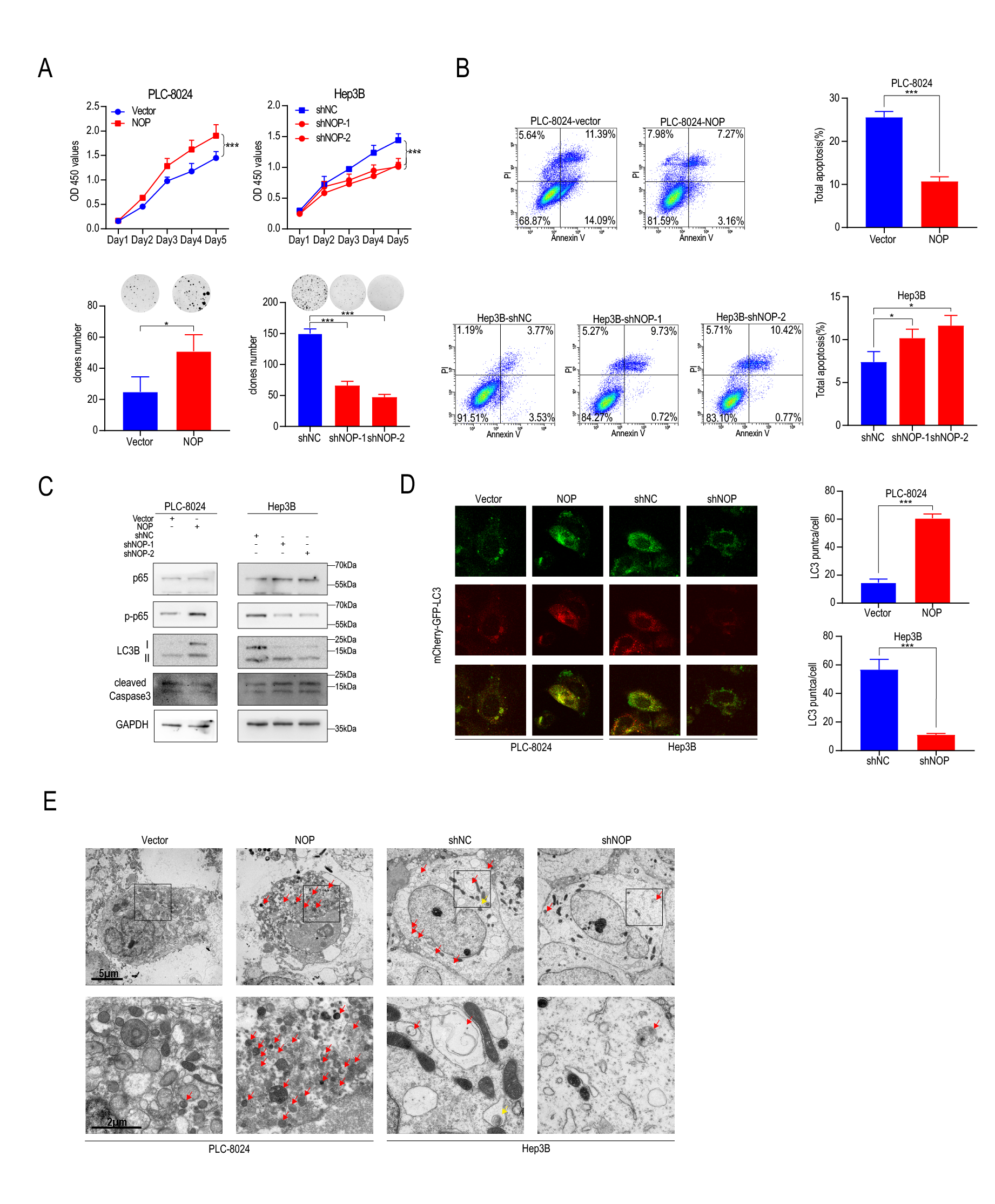

Supplement: Supplementary file 4 — SuppleFig2 [file 41420_2022_978_MOESM4_ESM.tif]

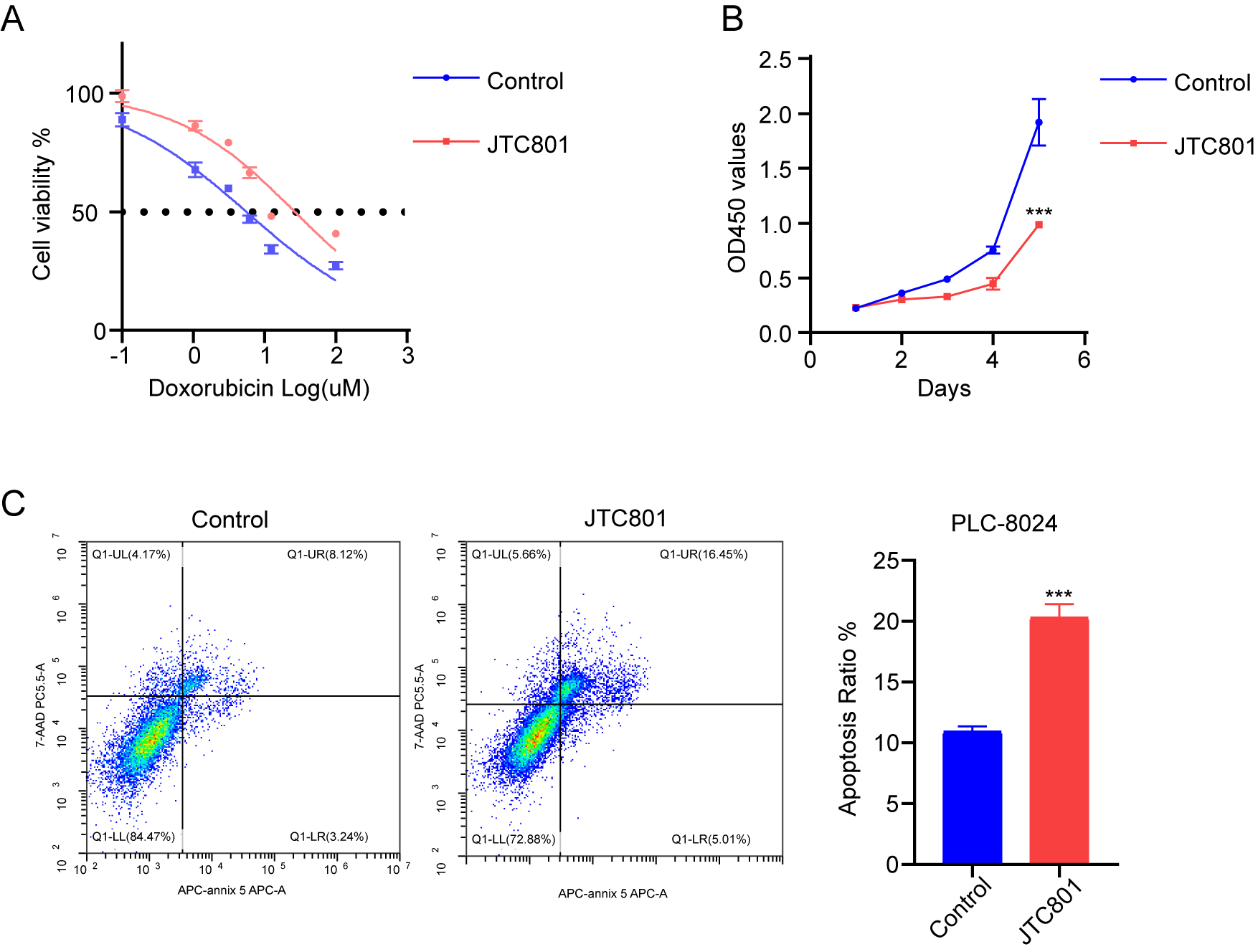

Supplement: Supplementary file 5 — SuppleFig3 [file 41420_2022_978_MOESM5_ESM.tif]
